# Transcriptional signatures of respiratory syncytial virus (RSV) infection using relaxed magnitude altitude score with hub-centric network analysis (RMAS-HCNA)

**DOI:** 10.3389/fgene.2026.1879778

**Published:** 2026-09-08

**Authors:** Mostafa Rezapour, David A. Ornelles, Stephen J. Walker, Colin E. Bishop, Sean V. Murphy, Metin Nafi Gurcan, Patrick M. McNutt, Anthony Atala

**Affiliations:** 1 Wake Forest Institute for Regenerative Medicine, Wake Forest University School of Medicine, Winston-Salem, NC, United States; 2 Department of Microbiology and Immunology, Wake Forest University School of Medicine, Winston-Salem, NC, United States; 3 Center for Artificial Intelligence Research, Wake Forest University School of Medicine, Winston-Salem, NC, United States

**Keywords:** airway organ tissue equivalent, host transcriptional response, human metapneumovirus, influenza A virus, NanoString, nested cross-validation, network-based gene selection, respiratory syncytial virus

## Abstract

**Introduction:**

Respiratory syncytial virus A (RSVA) and respiratory syncytial virus B (RSVB) may induce distinct airway host responses. We compared them with influenza A virus (IAV) and human metapneumovirus (hMPV) in airway organ tissue equivalents (OTEs) and developed the Relaxed Magnitude Altitude Score with Hub-Centric Network Analysis framework (RMAS-HCNA).

**Methods:**

OTEs were infected with RSVA-GFP5 or RSVB-GFP3 at low multiplicity of infection and profiled at 24 and 72 h using the NanoString nCounter Host Response Panel. Previous IAV and hMPV profiles were integrated. Analyses included source-matched differential expression, cross-virus ranking, network-based module selection, nested cross-validation, and Gene Ontology Biological Process enrichment.

**Results:**

RSVA showed the strongest temporal expansion and developed a late response centered on intracellular transport, localization, and organelle organization. RSVB remained the most restricted response, shifting from early inflammatory and signaling-associated activity to a later interferon-stimulated profile. IAV maintained a broad antiviral response, whereas hMPV combined limited induction with extensive late downregulation of stress, catabolic, metabolic, and transport-related processes. A shared antiviral component was present across infections, while downregulated responses were largely virus dependent. Under five-fold outer and four-fold inner nested cross-validation, the selected genes achieved a mean outer-test Macro F1 of 0.859 and balanced accuracy of 0.888, compared with 0.604 and 0.616 for the all-gene comparator.

**Discussion:**

The four viruses shared an antiviral foundation but followed distinct temporal and functional trajectories. The framework identified compact, biologically representative panels that generalized better than the complete measured gene set, although independent genome-wide and *in vivo* validation remains necessary.

## 1 Introduction

Respiratory syncytial virus (RSV) is a major cause of respiratory disease in infants and young children and contributes to severe illness among older adults, particularly those with underlying cardiopulmonary conditions (Hall et al., 2009; Zhou et al., 2012; Falsey and Walsh, 2005; Haber, 2018). RSV is classified into two antigenic groups, RSVA and RSVB, based largely on variation in the G glycoprotein (Kelleher et al., 2025). Although both groups infect the airway epithelium, differences in viral sequence, replication kinetics, and interactions with host cells may produce distinct transcriptional responses. These responses commonly involve interferon signaling, innate viral sensing, inflammation, cellular stress, and metabolic regulation (Barral-Arca et al., 2020; Dapat et al., 2021; Sonawane et al., 2019; Ressel et al., 2024), but their magnitude and timing depend on the viral strain, infection dose, sampling time, cell type, and experimental system.

Three-dimensional airway organ tissue equivalents (OTEs) provide an airway-like environment for studying respiratory infections because they preserve epithelial differentiation, stromal support, extracellular matrix, and an air-liquid interface more effectively than conventional two-dimensional cultures (Leach et al., 2023). Airway OTEs have been used to examine infections caused by influenza A virus (IAV), human metapneumovirus (hMPV), and parainfluenza virus type 3 (Rezapour et al., 2024a; Rezapour et al., 2024b; Rezapour et al., 2024c; Rezapour et al., 2025a).

In the present study, we compared the host transcriptional responses to RSVA and RSVB with those produced by IAV and hMPV at 24 and 72 h post infection (Rezapour et al., 2024a). This design allowed us to distinguish a common antiviral foundation from responses shared by selected viruses or associated with a particular infection and time point. Because the IAV/hMPV and RSV datasets originated from separate experiments, each virus was evaluated against its corresponding source-matched Mock control.

A central challenge in comparative transcriptomic analysis and machine learning classification is identifying compact gene panels that capture statistically supported and biologically meaningful expression differences. These panels should also preserve shared and virus-associated signals while limiting redundant selection of highly correlated genes from the same biological process. Ranking by P -value alone may favor small but highly consistent differences, whereas ranking by fold change alone may emphasize large but variable responses. In addition, selecting genes independently by statistical rank can retain several functionally related or strongly correlated candidates while underrepresenting other biological processes. These challenges are particularly important in experiments with limited sample sizes and low multiplicities of infection, where potentially reproducible signals must be preserved without producing unnecessarily large or redundant feature sets.

To address this challenge, we developed the Relaxed Magnitude Altitude Score with Hub-Centric Network Analysis framework, referred to as RMAS-HCNA. Relaxed Magnitude Altitude Score (RMAS) first ranks significant genes independently within each source-matched virus-time comparison by combining the absolute $\log_{2}$ fold change with the corresponding P -value. Genes receive higher RMAS values when they show both a larger expression difference and stronger statistical evidence.

The virus-specific RMAS results are then integrated through Multiple Gene-Set Ranking (MGSR). MGSR evaluates the infections supporting each gene, its expression direction, and its relative response strength. It subsequently assigns and ranks genes within biologically interpretable cross-virus groups. Thus, MGSR provides the ranked candidate groups that enter the network-selection stage.

For each MGSR group and time point, the top K candidates are entered into a STRING functional association network (Szklarczyk et al., 2025). Hub-Centric Network Analysis (HCNA) identifies connected components and network modules, ranks genes relative to the structure of their own modules, and retains up to q representative genes per module. This module-based selection reduces redundancy because several highly ranked candidates may belong to the same interferon, inflammatory, transport, or stress-associated process and may provide similar information. Limiting the contribution of each module allows the final panel to represent a broader range of biological signals.

The resulting panel provides a compact feature set for machine-learning classification. Selected genes may be used directly as predictor variables when the sample size is sufficient relative to the panel size and model complexity. In the present study, the number of samples was limited, so the standardized selected-gene expression matrix was reduced to the first two principal components (PC1 and PC2) (Groth et al., 2012), which served as predictors for balanced multiclass logistic regression (Nababan et al., 2024). This low-dimensional representation reduced the number of fitted parameters and limited overfitting (Rezapour et al., 2026a) while retaining the dominant joint expression patterns captured by the selected genes.

**FIGURE 1:**
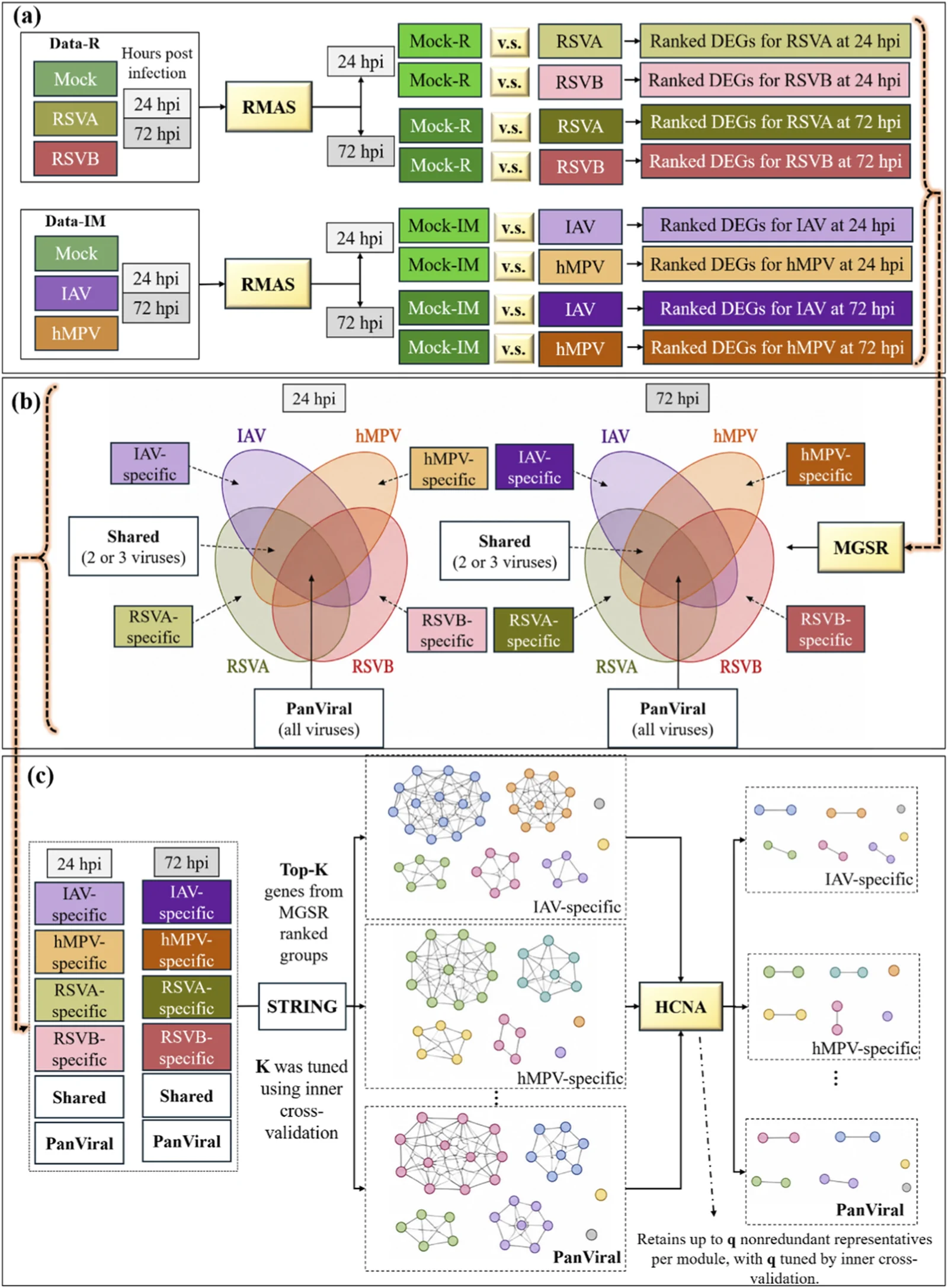
Overview of the RMAS-HCNA workflow within one representative outer-training partition. **(a)** Source-matched differential-expression analysis and RMAS ranking are performed independently for RSVA and RSVB relative to Mock-R and for IAV and hMPV relative to Mock-IM at 24 and 72 h post infection. **(b)** MGSR integrates the four virus-specific RMAS results at each time point and assigns significant genes to PanViral, Shared, IAV-specific, hMPV-specific, RSVA-specific, or RSVB-specific groups according to cross-virus support, expression direction, and relative response strength. **(c)** The top K MGSR-ranked genes from each biological group and time point enter the STRING functional-interaction network. HCNA identifies connected components and network modules, ranks genes within each module using complementary hub and stability measures, and retains up to q nonredundant representatives per module.

The complete workflow was embedded within nested cross-validation (Varma and Simon, 2006) to prevent held-out samples from influencing gene discovery, MGSR ranking, network construction, module selection, data harmonization, dimensionality reduction, hyperparameter optimization, or classifier training. Figure 1 summarizes the procedure within one representative outer-training partition. Biological interpretation was performed separately from classifier training. Genes showing consistent Upregulated or Downregulated responses across the outer-training folds were used for Gene Ontology Biological Process (GO-BP) enrichment analysis (Carbon et al., 2020). This analysis was used to compare conserved, shared, and virus-associated biological processes across RSVA, RSVB, IAV, and hMPV at 24 and 72 h post infection.

## 2 Data

### 2.1 NanoString data from RSV-infected OTEs

The airway OTEs were infected with RSVA-GFP5 (ViraTree, R125) or RSVB-GFP3 (ViraTree, R423), both of which were modified to include the enhanced green fluorescent protein (EGFP) gene that enabled visual tracking of infection and viral propagation. RSVA features a wild-type parent derived from the RSV strain A2, modified to express enhanced GFP *via* a gene cassette inserted between the P and M genes. This DNA fragment underwent multiple cloning steps within the same region in the full-length RSV cDNA plasmid. To stabilize the plasmid for bacterial propagation, silent mutations were introduced into the last three codons of the SH protein’s stop codon, and 112 nucleotides of the noncoding region downstream of the SH gene’s ORF were removed. The EGFP gene was positioned between the P and M genes of strain A2, becoming the fifth gene in the modified virus.

RSVB incorporates the EGFP gene between the NS2 and N genes of strain B1, making it the third gene in the modified virus. The wild-type G gene’s sequence was resistant to cloning into a full-length antigenomic cDNA in *E. coli*, prompting the replacement of the wild-type G gene with a codon-optimized version. ViraTree reported that RSVB-GFP3 replicates to wild-type titers in cell culture. This comparison with wild-type titers was based on the supplier’s characterization and was not independently quantified in the airway OTE system. However, infection progression under the specific 3D OTE culture conditions was experimentally verified by monitoring EGFP fluorescence over time, which showed comparable temporal progression of infection and viral propagation. Because of the cellular heterogeneity of the OTEs, viral yield was not quantified or normalized on a per-cell basis.

To initiate the experiments, the OTEs were acclimatized in a modified PneumaCult ALI Medium at 37 °C in a CO_2_-enriched environment. Each OTE, containing approximately 160,000 epithelial cells, was rinsed with warm Hank’s balanced saline solution to remove any impurities. The viral strains were then diluted in iDMEM to an approximate multiplicity of infection (MOI) of 0.25 as determined by a fluorescent-focus assay using the monkey LLC-MK2 cell line. The infectious titers used to calculate the nominal MOI were those provided by the supplier. This dilution was applied to the apical surface of each OTE to ensure a controlled and effective infection process. After allowing the virus an hour to infect the cells under gentle rocking conditions, the OTEs were kept under standard growth conditions to facilitate ongoing observation of viral dynamics within this intricate model system.

Mock-infected controls (Mock-R) were processed in parallel using identical culture conditions, medium, and handling procedures but without viral exposure. These samples served as essential controls, ensuring that any observed transcriptional or cellular changes could be attributed specifically to RSV infection rather than experimental artifacts.

To characterize host responses to RSV infection at 24- and 72-h post infection (hpi), we employed the NanoString nCounter® Analysis System. Using the nCounter® Host Response Panel, which includes probes for 785 human genes and 12 internal reference genes relevant to infection susceptibility, interferon signaling, and immune cell activation, we extracted total RNA from both infected and mock-infected OTEs. Following hybridization with NanoString probes and digital quantification of target transcripts, this approach enabled a comprehensive assessment of gene expression changes associated with RSVA and RSVB infections. Five biological replicates were included for RSVA, RSVB, and Mock-R at both 24 and 72 h post infection, resulting in 30 RSV-experiment samples.

### 2.2 NanoString data from IAV- and hMPV-infected OTEs

The IAV, hMPV, and their matched Mock controls (Mock-IM) data were generated previously using the same airway OTE platform (Rezapour et al., 2024a). OTEs were infected with IAV strain A/Puerto Rico/8/1934 (H1N1), which contains an EGFP-NS1 fusion, or hMPV strain CAN97-83, which contains EGFP upstream of the N gene. Viruses were applied to the apical surface at a nominal multiplicity of infection of 0.1 and incubated for 1 h under gentle rocking at 37 °C. Mock-IM controls underwent the same procedures without viral exposure. Six biological replicates were available for IAV, hMPV, and Mock-IM at both 24 and 72 h post infection, resulting in 36 samples. For this dataset, expression was also measured using the same NanoString nCounter Host Response Panel.

## 3 Methodology

### 3.1 Analytical objective and RMAS-HCNA framework

The RMAS-HCNA framework consisted of three connected gene-analysis stages. RMAS ranked genes within each source-matched virus comparison according to expression magnitude and statistical evidence. MGSR integrated the four virus-specific rankings at each time point and organized genes according to their cross-virus support, expression direction, and relative response strength. HCNA then selected representative genes from functional network modules. Detailed algorithms for RMAS, MGSR, HCNA, and the nested cross-validation workflow are provided in Supplementary Algorithms S1–S4 in Supplementary Materials.

### 3.2 Source-matched differential-expression analysis

The IAV/hMPV and RSV datasets were generated in separate experiments. Differential-expression analysis was therefore performed within each experimental source and time point. IAV and hMPV were compared with Mock-IM, while RSVA and RSVB were compared with Mock-R. This produced eight virus-time comparisons: IAV *versus* Mock-IM, hMPV *versus* Mock-IM, RSVA *versus* Mock-R, and RSVB *versus* Mock-R at 24 and 72 h.

Each comparison was recalculated within every training partition of the nested cross-validation. Validation and test samples did not contribute to the calculation of group means, $\log_{2}$ fold changes, P -values, RMAS values, or gene ranks. Gene expression between each infected group and its source-matched Mock group was compared using a two-sided Welch t -test (De Winter 2013) with unequal variances.

### 3.3 Relaxed magnitude altitude score (RMAS)

RMAS was applied independently to each of the eight source-matched virus-time comparisons. Ranking by P -value alone may favor small but highly consistent expression differences, while ranking by fold change alone may favor large but variable differences. RMAS combines both quantities and therefore gives the highest priority to genes with a large expression difference and strong statistical evidence.

For gene g , RMAS was defined as

$${\text{RMAS}}_{g}=|\log_{2}\left({\text{FC}}_{g}\right)|\left[-\log_{10}\left(P_{g}\right)\right],$$

where $P_{g}$ is the P -value from the two-sided Welch t -test. Genes with P<0.05 were retained as initial candidates and classified as Upregulated or Downregulated according to the sign of their $\log_{2}$ fold change.

The nominal P -value was used only for the initial candidate stage because the limited sample size and low multiplicity of infection could cause moderate but reproducible responses to be removed by a multiple-testing-only filter. This criterion did not determine the final gene panel. Candidate genes were subsequently evaluated through MGSR integration, module-aware network selection, outer-fold stability assessment, and nested cross-validation. The complete calculation of group means, $\log_{2}$ fold changes, Welch-test P -values, expression directions, RMAS values, and ranks are provided in Supplementary Algorithm S1.

### 3.4 Multiple gene-set ranking (MGSR)

RMAS prioritizes genes within each individual virus-versus-source-matched-Mock comparison, but it does not determine whether a response is conserved across all viruses, shared by a subset of viruses, or associated with one infection. MGSR was therefore used to integrate the RMAS results for IAV, hMPV, RSVA, and RSVB separately at 24 and 72 h.

Because RMAS values and absolute $\log_{2}$ fold changes can have different numerical ranges across virus comparisons, both quantities were converted to percentile ranks within each virus-time comparison. Percentile ranks were calculated across all 773 measured genes before significant genes were assigned to candidate groups. Tied values received the average of their rank positions, and larger RMAS or absolute-fold-change values received larger percentile ranks. This transformation allowed genes to be evaluated relative to the complete measured-gene space of their corresponding comparison and placed the four virus-specific results on a common ranking scale.

Genes significant in all four viruses with the same expression direction were assigned to the PanViral group. Genes significant in at least two viruses but not meeting the PanViral definition were assigned to the Shared group. Shared genes were classified as Upregulated, Downregulated, or Mixed according to the consistency of their directions across the supporting viruses. Genes significant in only one virus were assigned to the corresponding IAV-specific, hMPV-specific, RSVA-specific, or RSVB-specific group.

The group-specific scores incorporated both the breadth and strength of the response. The PanViral score combined the minimum and average RMAS percentile ranks across all four viruses with the average absolute- $\log_{2}$ -fold-change percentile rank. Including the minimum RMAS percentile favored genes with consistently strong responses and prevented an exceptionally strong result in one virus from compensating for weak responses in the others. The Shared score also incorporated the proportion of supporting viruses and calculated its RMAS and fold-change components only across those viruses. Virus-specific scores combined the RMAS and absolute-fold-change percentile ranks from the single supporting virus.

Genes were ranked by decreasing group-specific score within each biological group and time point. These ranked PanViral, Shared, and virus-specific candidate groups formed the inputs to HCNA. The complete percentile-rank calculation, significance indicators, group definitions, scoring functions, direction assignments, and candidate-ranking procedure is provided in Supplementary Algorithm S2.

### 3.5 Hub-centric network analysis (HCNA)

RMAS prioritizes genes within each source-matched virus comparison according to expression magnitude and statistical evidence, while MGSR integrates these results across viruses and organizes genes according to cross-virus support, expression direction, and relative response strength. However, these stages do not directly account for known functional associations among the proteins encoded by the candidate genes. HCNA was therefore used to identify functional network modules and select representative genes from each module. This approach preserved broad biological coverage while limiting the repeated selection of functionally related or highly correlated genes.

For each MGSR biological group and time point, the top K ranked genes were entered into the human STRING v12.0 functional association network using a minimum interaction score of 600 (Szklarczyk et al., 2025). When both Upregulated and Downregulated genes were available but the top K candidates represented only one direction, the lowest-ranked retained gene was replaced with the highest-ranked candidate from the missing direction.

Networks were constructed and analyzed using NetworkX v3.4.2 (Hagberg et al., 2008). Connected components containing more than three genes were divided into modules using weighted Leiden clustering (Traag et al., 2019), implemented with python-igraph v1.0.0 and leidenalg v0.11.0 at a resolution of 1.0. Components containing two or three genes were retained as individual modules, while genes without qualifying STRING associations were preserved as singleton modules. Retaining singleton modules prevented statistically supported MGSR candidates from being excluded solely because they lacked recorded associations above the selected STRING threshold.

Within each detected module M , let g∈M denote a gene. Five complementary network measures were calculated for each gene: weighted degree $d_{M}^{w}\left(g\right)$ , PageRank $PR_{M}\left(g\right)$ , weighted betweenness centrality $BC_{M}\left(g\right)$ , core number $k_{M}\left(g\right)$ , and bootstrap maximum-core stability $s_{M}\left(g\right)$ . These measures represent total association strength, influence through neighboring genes, participation in shortest communication paths, membership in densely connected cores, and stability of core membership under network resampling, respectively. Complete mathematical definitions of the five measures are provided in the Supplementary Materials.

Each measure was converted to an ascending percentile rank within its corresponding module. Let $Q_{M}\left(Z_{M}\left(g\right)\right)$ denote the percentile rank of network measure $Z_{M}\left(g\right)$ among all genes in module M . Larger network-measure values received larger percentile ranks, tied values received the average of their rank positions, and the gene in a singleton module received a percentile rank of 1. The hub score of gene g was defined as

$$H_{M}\left(g\right)=\frac{\;Q_{M}\left(d_{M}^{w}\left(g\right)\right)+Q_{M}\left(PR_{M}\left(g\right)\right)+Q_{M}\left(BC_{M}\left(g\right)\right)+Q_{M}\left(k_{M}\left(g\right)\right)+Q_{M}\left(s_{M}\left(g\right)\right)}{5}.$$

A larger $H_{M}\left(g\right)$ indicated that the gene occupied an important position according to several complementary network properties rather than only one centrality measure. Genes were ranked within each module by decreasing hub score, followed by decreasing MGSR score and bootstrap stability. Detailed definitions of the individual network measures, percentile-rank calculation, bootstrap procedure, and representative-selection rules, and the complete HCNA workflow is presented in Supplementary Algorithm S3.

### 3.6 Nested cross-validation for RMAS-HCNA gene selection and classification

The complete RMAS-HCNA framework was embedded within nested cross-validation to prevent information leakage during gene discovery, network analysis, data harmonization, hyperparameter selection, dimensionality reduction, and classifier training. Performing any of these steps before cross-validation could allow information from held-out samples to influence the selected gene panels and produce overly optimistic estimates of predictive performance.

The IAV/hMPV and RSV datasets were aligned using their 773 shared genes while preserving experimental-source identities. Mock-IM and Mock-R were assigned to a common Mock outcome for five-class classification, but they remained distinct during source-matched differential-expression analysis and batch-effect estimation. Cross-validation folds were stratified by source-specific biological group and time point to preserve the representation of Mock-IM, IAV, hMPV, Mock-R, RSVA, and RSVB samples at 24 and 72 h.

Five outer folds were used to estimate generalization performance. In each outer iteration, one fold was reserved as the outer-test set, while the remaining four folds formed the outer-training set. Four inner folds were generated within the outer-training set and used for gene-selection and classifier hyperparameter optimization.

Within each inner-training partition, RMAS was applied independently to the eight source-matched virus-time comparisons according to Supplementary Algorithm S1. MGSR then integrated the four virus-specific RMAS results at each time point according to Algorithm S2. For each MGSR group and time point, the top K ranked candidates entered HCNA. STRING network construction, module detection, network-measure calculation, bootstrap maximum-core stability, hub-score calculation, and within-module ranking were performed using the inner-training samples only, as described in Supplementary Algorithm S3.

Separate 24- and 72-h training-only parametric empirical-Bayes ComBat models (Johnson et al., 2007) were fitted to $\log_{2}$ (expression +1) values using source-matched Mock samples from the corresponding training partition. Experimental source was treated as the batch variable. The location and scale parameters estimated from the training Mock samples were frozen and applied to the corresponding training and validation or test samples.

Note that ComBat was not used for differential-expression analysis, RMAS, MGSR, STRING network construction, module detection, network-measure calculation, initial hub ranking, or RMAS-anchor selection. Harmonized expression values were used only for the within-module correlation redundancy filter, PCA, and classification.

The inner cross-validation evaluated four hyperparameters: $K\in \left\{5,10,15,20,25,30,35,40\right\},$ where K was the number of MGSR-ranked genes entering STRING from each biological group and time point; $q\in \left\{1,2,\ldots ,10\right\},$ where q was the maximum number of representatives retained from each network module; $A\in \left\{0,2,5\right\},$ where A was the maximum number of direction-aware RMAS anchors selected from each virus-time comparison; and $C\in \left\{0.01,0.1,1,10\right\},$ where C was the inverse regularization-strength parameter of the logistic-regression classifier.

For each combination of K , q , and A , the HCNA representatives and RMAS anchors were combined and deduplicated to form the final candidate panel. Direction-aware anchors were selected from the original RMAS tables to preserve highly ranked genes that might not be retained through network position alone. PCA was then fitted using the standardized training values, and PC1 and PC2 were retained as predictors. A balanced L2-regularized (Bühlmann and Yu, 2003) multiclass logistic-regression model was trained to classify Mock, IAV, hMPV, RSVA, and RSVB samples.

Mean Macro F1 (Opitz, 2024) across the four inner-validation folds was used as the primary hyperparameter-selection measure. Configurations within one standard error of the highest mean Macro F1 were retained. The final configuration was selected from this eligible set by prioritizing lower infected-to-Mock error, higher Mock-separation balanced accuracy, lower Mock-to-infected error, smaller final panel size, and smaller hyperparameter values.

After hyperparameter selection, the complete RMAS-HCNA workflow was repeated using all samples in the corresponding outer-training set. Differential-expression analysis, RMAS, MGSR, HCNA, RMAS-anchor selection, ComBat estimation, scaling, PCA, and classifier fitting were repeated in the same order. The resulting ComBat transformation, scaler, PCA model, and logistic-regression classifier were then applied once to the untouched outer-test samples.

An all-gene comparator was evaluated using the same inner and outer folds. It used all 773 measured genes and followed the same training-only ComBat, scaling, two-component PCA, logistic-regression, and C -tuning procedures. It did not use RMAS, MGSR, HCNA, or RMAS-anchor selection. This design allowed the compact RMAS-HCNA panels and the complete measured-gene space to be compared under otherwise identical validation conditions.

The outer-test predictions from the five folds were pooled so that each sample received exactly one prediction from a model that had not used that sample during gene discovery, network analysis, harmonization, hyperparameter selection, dimensionality reduction, or classifier training. Performance was summarized using Macro F1 and balanced accuracy (Brodersen et al., 2010). Gene-selection stability was calculated as the proportion of outer folds in which each gene was included in the final RMAS-HCNA panel.

The complete fold structure, order of training-only analytical steps, hyperparameter evaluation, one-standard-error selection procedure, outer-test assessment, all-gene comparison, and gene-selection-frequency calculation is provided in Supplementary Algorithm S4.

### 3.7 Gene ontology biological process (GO-BP) enrichment and cross-virus comparison

To characterize the biological processes associated with reproducible host transcriptional responses, GO-BP enrichment analysis (Carbon et al., 2020) was performed using genes that remained significant across the outer cross-validation folds. This analysis was based on the training-only differential-expression results generated independently for IAV, hMPV, RSVA, and RSVB at 24 and 72 h. It was conducted separately from classifier training and did not use the final classification gene panels.

For each virus and time point, the gene-level results from the five outer-training folds were combined. A gene was defined as “stably upregulated” when it had a P -value below 0.05 and a positive $\log_{2}$ fold change in at least four of the five outer folds, with no fold showing significant downregulation. Similarly, a gene was defined as “stably downregulated” when it had a P -value below 0.05 and a negative $\log_{2}$ fold change in at least four of the five folds, with no fold showing significant upregulation. Stable genes were therefore identified separately for each virus, time point, and expression direction, which produced 16 gene sets corresponding to four viruses, two time points, and two directions.

For descriptive ranking, stable genes were ordered first by the number of outer folds in which they were significant and then by their average RMAS value across the significant folds. The average absolute $\log_{2}$ fold change and other fold-specific statistics were retained for interpretation. This ranking was used to summarize the most reproducible genes but did not change the membership of the stable-gene sets used for enrichment.

The GO analysis background was defined as the set of genes measured in every outer-fold differential-expression file, which resulted in a common background of 773 NanoString-measured genes. Gene symbols in the stable-gene sets and the measured background were mapped to Entrez Gene identifiers (Maglott et al., 2011) in R version 4.5.2 under Bioconductor version 3.22 (Gentleman et al., 2004), using AnnotationDbi version 1.72.0 and the human annotation package org.Hs.eg.db version 3.22.0.

GO-BP over-representation analysis was performed independently for each of the 16 stable-gene sets using the enrichGO function from clusterProfiler version 4.18.4 (Wu et al., 2021; Yu et al., 2012). The mapped Entrez identifiers corresponding to the complete NanoString-measured gene background were supplied as the enrichment universe. GO-BP gene sets containing fewer than 10 genes or more than 500 genes were excluded. The enrichGO retrieval thresholds for P -values were set to one to retrieve the complete GO result space before applying the study-specific GO-term significance criterion.

## 4 Results

### 4.1 RMAS identifies reproducible virus- and time-dependent transcriptional responses

**FIGURE 2:**
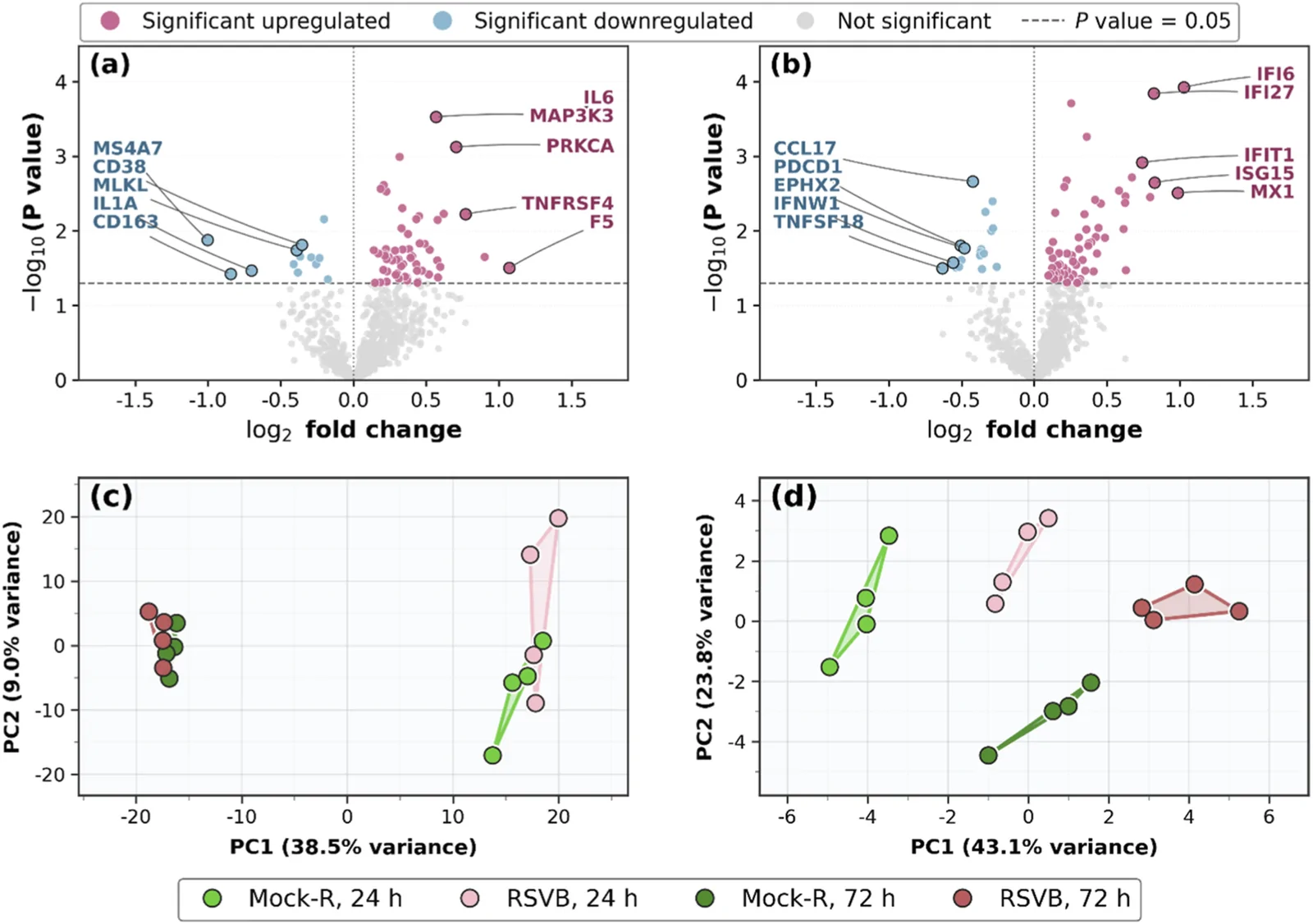
Training-only RMAS analysis of the time-dependent RSVB transcriptional response. Volcano plots show RSVB relative to its source-matched Mock-R control within outer-training fold 1 at **(a)** 24 h and **(b)** 72 h post infection. Significant upregulated genes are shown in pink, significant downregulated genes are shown in blue, and nonsignificant genes are shown in gray. The five highest-ranked upregulated and downregulated genes according to RMAS are labeled. The horizontal dashed line indicates the significance threshold. PCA was performed using the 16 RSVB and Mock-R samples in outer-training fold 1 with **(c)** all 773 measured genes and **(d)** the nonduplicated union of the five highest-ranked upregulated and downregulated genes from each time point.

The training-only differential-expression and RMAS-ranking procedure was first examined using RSVB in outer-training fold 1 (Figure 2). At 24 h, 59 genes were significantly upregulated and 13 were significantly downregulated relative to the source-matched Mock-R samples. The upregulated response was led by *IL6*, *PRKCA*, *MAP3K3*, *TNFRSF4*, and *F5*, whereas *MS4A7*, *CD163*, *IL1A*, *MLKL*, and *CD38* were the highest-ranked downregulated genes. This early profile combined an inflammatory component with changes in intracellular signaling and immune-associated genes.

At 72 h, the overall size of the RSVB response remained comparatively limited, with 60 significantly upregulated genes and 18 significantly downregulated genes, but its leading components differed from those observed at 24 h (Figure 2b). *IFI6*, *IFI27*, *MX1*, *ISG15*, and *IFIT1* became the highest-ranked upregulated genes, whereas *CCL17*, *TNFSF18*, *PDCD1*, *IFNW1*, and *EPHX2* led the downregulated ranking. None of the five leading upregulated or downregulated genes at 24 h remained among the corresponding five genes at 72 h. RSVB therefore progressed from an early response led by inflammatory and signaling-associated genes to a later response centered on coordinated interferon-stimulated effectors. This temporal restructuring occurred without a major increase in the total number of significant genes, which indicates that the later response reflected a change in its transcriptional composition rather than a broad expansion.

The capacity of RMAS to concentrate infection-associated transcriptional structure was examined by comparing PCA based on all measured genes with PCA based on the leading RMAS-ranked genes. When all 773 genes were included, PC1 and PC2 explained 38.5% and 9.0% of the variance, respectively, for a combined 47.5% (Figure 2c). The dominant organization of the projection reflected the difference between 24 and 72 h. Within each time point, however, the RSVB and Mock-R samples remained less clearly separated, particularly at 72 h, where the infected and Mock-R samples occupied closely positioned regions of the PC1-PC2 space.

The five highest-ranked upregulated and downregulated genes from each time point formed a nonduplicated set of 20 RMAS-ranked genes. Restricting PCA to these genes increased the variance explained by PC1 and PC2 to 43.1% and 23.8%, respectively, for a combined 66.9% (Figure 2d). The reduced gene set resolved four distinct condition-time groups. RSVB samples were separated from their source-matched Mock-R samples at both 24 and 72 h, while the early and late samples also remained clearly distinguished. The gain was therefore not limited to a higher percentage of explained variance. RMAS redirected the leading components toward genes that captured both infection status and temporal progression, rather than the broader sources of variation represented by the full NanoString panel. Because gene ranking and PCA were performed within the same outer-training partition, these projections provide a training-only demonstration of the transcriptional structure captured by RMAS rather than an independent assessment of classification performance.

**FIGURE 3:**
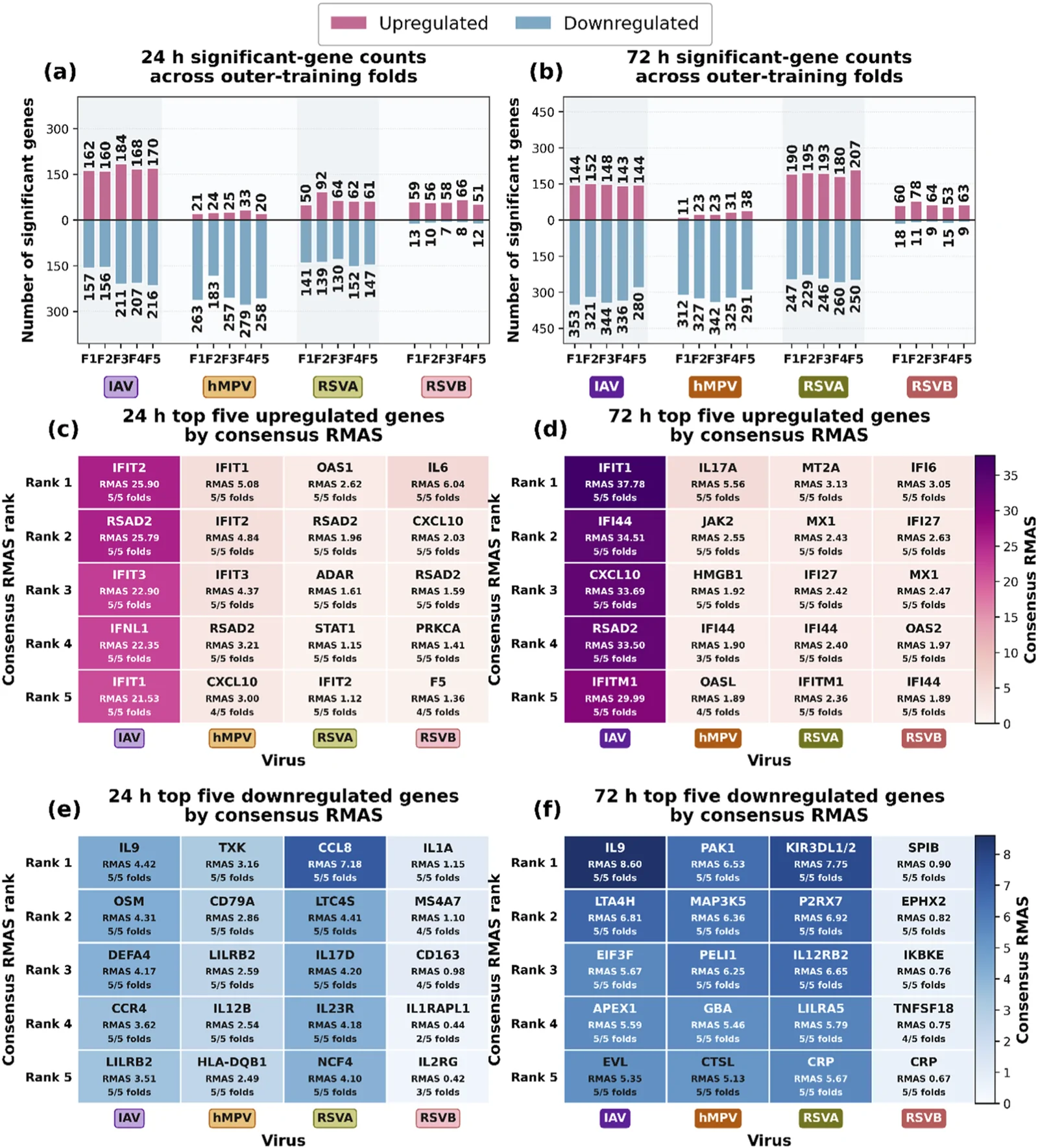
Cross-fold reproducibility and consensus RMAS rankings of virus- and time-dependent transcriptional responses. Numbers of significantly upregulated and downregulated genes in each of the five outer-training folds are shown for IAV, hMPV, RSVA, and RSVB at **(a)** 24 h and **(b)** 72 h post infection. IAV and hMPV were compared with Mock-IM, while RSVA and RSVB were compared with Mock-R. Heatmaps show the five highest-ranked genes according to consensus RMAS for **(c)** upregulated genes at 24 h, **(d)** upregulated genes at 72 h, **(e)** downregulated genes at 24 h, and **(f)** downregulated genes at 72 h. Each heatmap cell reports the gene, consensus RMAS value, and number of outer-training folds in which the gene was significant in the indicated direction. Consensus RMAS was calculated across all five outer-training folds, with the direction-specific RMAS assigned a value of zero in folds where the gene was not significant in that direction. Darker shading indicates a higher consensus RMAS.

Figure 2 presents RSVB in outer-training fold 1 as a representative example of this procedure. The complete training-only volcano plots are provided in Supplementary Figures S1–S8. Figure 3 summarizes these fold-specific results across all five outer-training folds by comparing the numbers of significantly upregulated and downregulated genes and the consensus RMAS rankings for IAV, hMPV, RSVA, and RSVB at 24 and 72 h.

The cross-fold comparison revealed four distinct transcriptional response patterns, which differed in breadth, predominant direction, and temporal progression (Figures 3a,b). At 24 h, IAV produced the broadest and most nearly bidirectional response, with 160–184 significantly upregulated genes and 156 to 216 significantly downregulated genes across the five outer-training folds (Figure 3a). hMPV also produced an extensive response, but it was strongly weighted toward transcriptional decreases: only 20 to 33 genes were upregulated, whereas 183 to 279 were downregulated. RSVA showed an intermediate response that was also biased toward downregulation, with 50–92 upregulated genes and 130 to 152 downregulated genes. RSVB was distinct from the other three viruses because its response was both smaller and inductive, with 51–66 upregulated genes but only 7 to 13 downregulated genes.

The 24-h results therefore separated the viruses into different response architectures. IAV initiated a broad response in both directions, hMPV was characterized primarily by extensive downregulation, RSVA showed a more moderate downregulation-dominant response, and RSVB produced a compact response composed mainly of upregulated genes. These patterns were reproduced across all five outer-training folds, despite changes in the individual samples included in each training partition.

By 72 h, the differences among the viral responses became more notable (Figure 3b). IAV maintained a broad response, but its temporal expansion occurred primarily through downregulation. Its upregulated component ranged from 143 to 152 genes, whereas its downregulated component increased to 280 to 353 genes. hMPV followed a similar directional pattern. The number of upregulated genes remained limited at 11 to 38, while the downregulated response expanded to 291 to 342 genes. Thus, the later IAV and hMPV profiles were both characterized by extensive transcriptional decreases, although IAV retained a substantially larger upregulated component than hMPV.

RSVA followed a different temporal trajectory. Its upregulated response increased from 50 to 92 genes at 24 h to 180 to 207 genes at 72 h, while its downregulated response increased from 130 to 152 genes to 229 to 260 genes. RSVA therefore showed the strongest temporal expansion of the four viruses and developed a broad bidirectional profile at 72 h. RSVB again remained distinct. It retained a compact, upregulation-dominant response, with 53–78 upregulated genes and only 9 to 18 downregulated genes. The four viruses consequently differed not only in the number of significant genes but also in how their responses progressed over time: IAV and hMPV expanded mainly through downregulation, RSVA expanded strongly in both directions, and RSVB remained comparatively restricted.

Cross-fold consensus RMAS rankings showed that these distinct response patterns were imposed on a recurrent antiviral component (Figures 3c,d). At 24 h, the five leading IAV genes were *IFIT2*, *RSAD2*, *IFIT3*, *IFNL1*, and *IFIT1*. Four of these genes, *IFIT1*, *IFIT2*, *IFIT3*, and *RSAD2*, were also present among the five leading hMPV genes, with *CXCL10* completing the hMPV ranking. This overlap showed that IAV and hMPV shared an early IFIT- and *RSAD2*-centered response despite their very different overall distributions of upregulated and downregulated genes.

RSVA retained part of this early antiviral program through *RSAD2* and *IFIT2*, but its remaining leading genes, *OAS1*, *ADAR*, and *STAT1*, distinguished its response from those of IAV and hMPV. RSVB showed the most distinct leading profile at 24 h. Its ranking was led by *IL6*, *CXCL10*, *RSAD2*, *PRKCA*, and *F5*, which was consistent with the inflammatory and signaling-associated profile observed in Figure 2a. *RSAD2* was the only gene ranked among the five leading upregulated genes for all four viruses at 24 h. It therefore represented the most consistent component of the early cross-virus response.

At 72 h, IAV remained centered on an interferon-associated profile led by *IFIT1*, *IFI44*, *CXCL10*, *RSAD2*, and *IFITM1* (Figure 3d). hMPV showed a notable change from its early IFIT-dominated ranking and was instead led by *IL17A*, *JAK2*, and *HMGB1*, with *IFI44* and *OASL* providing its remaining interferon-associated components. RSVA and RSVB became more similar to one another at this time point. Their leading rankings shared *MX1*, *IFI27*, and *IFI44*. RSVA additionally contained *MT2A* and *IFITM1*, whereas RSVB contained *IFI6* and *OAS2*. This convergence was consistent with the late interferon-stimulated RSVB profile shown in Figure 2b. *IFI44* appeared among the five leading upregulated genes for all four viruses at 72 h, although it was detected in fewer outer-training folds for hMPV than for IAV, RSVA, or RSVB. The principal recurrent signal therefore shifted from *RSAD2* and IFIT-family genes at 24 h toward *IFI44* and related interferon-stimulated genes at 72 h.

The downregulated rankings showed considerably less similarity among the four infections (Figures 3e,f). At 24 h, the highest-ranked downregulated genes were *IL9* for IAV, *TXK* for hMPV, *CCL8* for RSVA, and *IL1A* for RSVB. At 72 h, the corresponding leading genes were *IL9*, *PAK1*, *KIR3DL1/2*, and *SPIB*. Across the two time points, few genes appeared among the five leading downregulated genes for more than one virus. This limited overlap contrasted with the repeated presence of IFIT, IFI, OAS, MX, and *RSAD2* family members in the upregulated rankings.

### 4.2 MGSR separates conserved, shared, and virus-specific gene responses

**FIGURE 4:**
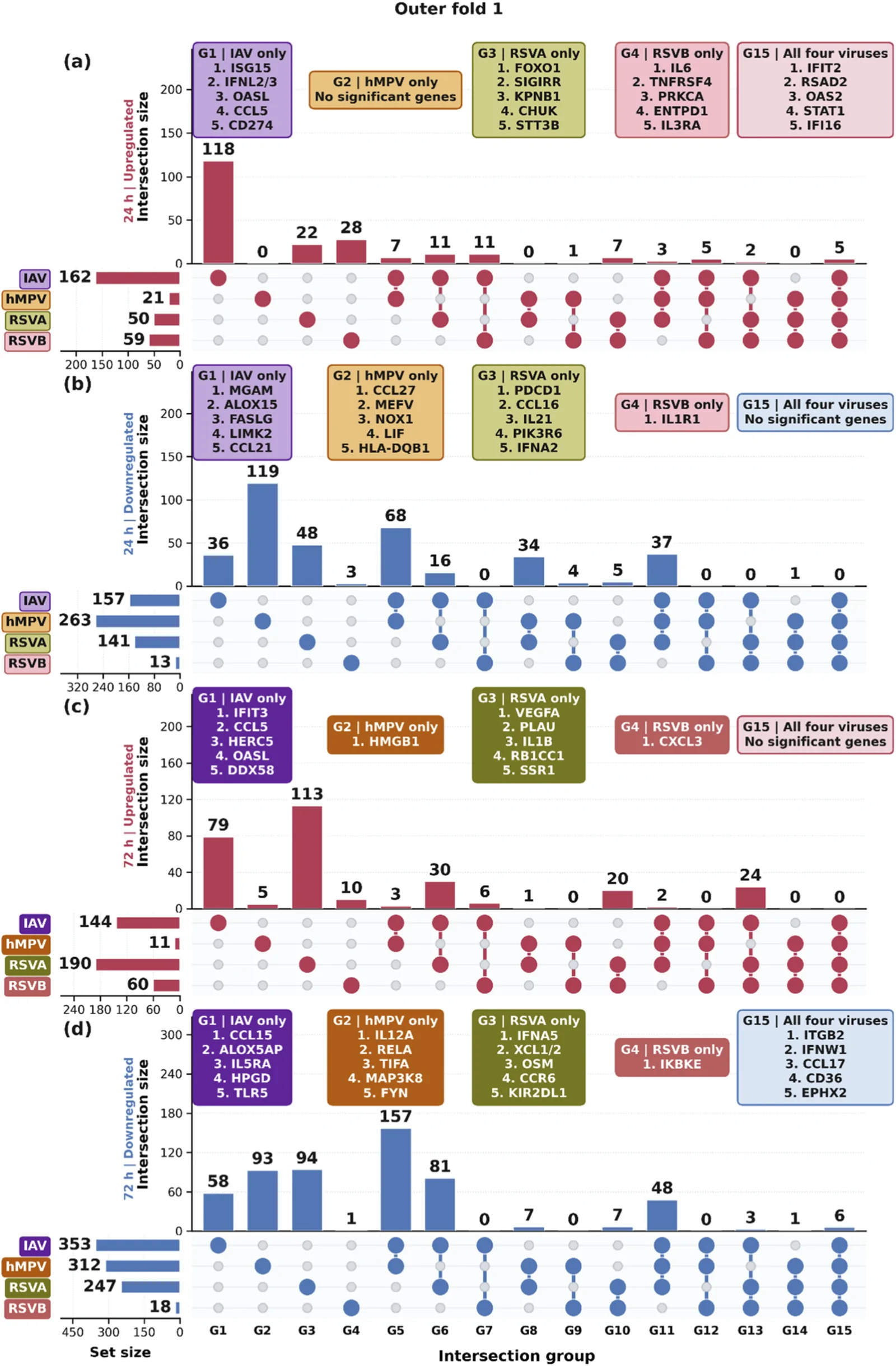
Direction-specific exact intersections of virus-associated genes in outer fold 1. UpSet plots show the exact intersections among genes significantly expressed by IAV, hMPV, RSVA, and RSVB within the outer-training data of outer fold 1. Panels show **(a)** upregulated genes at 24 h, **(b)** downregulated genes at 24 h, **(c)** upregulated genes at 72 h, and **(d)** downregulated genes at 72 h. Horizontal bars show the total gene-set size for each virus. Vertical bars show the number of genes in each exact intersection, while the membership matrix identifies the viruses represented in each group. G1 through G4 denote genes specific to IAV, hMPV, RSVA, and RSVB, respectively, while G15 denotes genes shared by all four viruses. Boxes list up to five leading genes from the corresponding virus-specific or PanViral MGSR candidate ranking.

RMAS (Figures 2, 3) summarized each virus independently, whereas MGSR determined whether the same significant genes were specific to one infection, shared by selected infections, or common to all four viruses within the same time point and expression direction. Outer fold one is shown as a representative example in Figure 4. The corresponding exact-intersection analyses for outer folds two through five are provided in Supplementary Figures S9–S12, respectively.

At 24 h, the upregulated response combined a compact common antiviral component with a much larger IAV-specific program. In outer fold 1, 118 of the 162 IAV-upregulated genes were significant only for IAV (Figure 4a). This group included *ISG15*, *IFNL2/3*, *OASL*, *CCL5*, and *CD274*, which showed that the broad early IAV response extended beyond the genes induced by the other viruses. RSVA and RSVB also contained distinct early components, but these were considerably smaller. Of the 50 RSVA-upregulated genes, 22 were specific to RSVA and were led by *FOXO1*, *SIGIRR*, *KPNB1*, *CHUK*, and *STT3B*. Of the 59 RSVB-upregulated genes, 28 were specific to RSVB and were led by *IL6*, *TNFRSF4*, *PRKCA*, *ENTPD1*, and *IL3RA*. This RSVB-specific group was consistent with the inflammatory and signaling-associated early profile observed in Figure 2.

The limited hMPV-upregulated response showed a different organization. None of the 21 hMPV-upregulated genes in outer fold one was specific to hMPV. Instead, these genes occurred almost entirely in intersections that also included IAV or all four viruses. This pattern was maintained across other folds: the IAV-specific upregulated group contained 114 to 134 genes, compared with 22–50 for RSVA, 20 to 32 for RSVB, and only zero to four for hMPV. Thus, the early hMPV-induced response largely represented a subset of the broader antiviral response observed for IAV and the other infections rather than a distinct hMPV-specific program.

Despite these differences, five genes formed an exact four-virus upregulated intersection in outer fold 1: *IFIT2*, *RSAD2*, *OAS2*, *STAT1*, and *IFI16*. The corresponding common four-virus group contained three to six genes in every outer fold. *RSAD2*, *OAS2*, and *STAT1* retained exact four-virus membership in all five folds, which identifies them as the most reproducible components of the early common response. The 24-h upregulated profile therefore consisted of a small but stable antiviral core surrounded by larger infection-associated components, particularly the broad IAV-specific response.

The 24-h downregulated response did not contain a comparable common four-virus core. In outer fold 1, hMPV contributed the largest virus-specific group, with 119 genes, while 68 genes were jointly downregulated by IAV and hMPV and another 37 were shared by IAV, hMPV, and RSVA. The IAV- and RSVA-specific groups contained 36 and 48 genes, respectively, whereas only three genes were specific to RSVB (Figure 4b). No gene was downregulated across all four viruses in this fold. Across all five folds, the hMPV-specific group contained 56 to 119 genes, the exact IAV-hMPV intersection contained 68 to 97 genes, and the IAV-hMPV-RSVA intersection contained 23 to 48 genes. In contrast, the exact four-virus downregulated intersection contained only zero to three genes and was absent in two folds. These results show that early transcriptional decreases were concentrated primarily in hMPV and in responses shared by hMPV with IAV or RSVA, rather than in a stable response common to all four infections.

The organization of the upregulated response changed substantially by 72 h, primarily because of the expansion of the RSVA-specific component. In outer fold 1, 113 of the 190 RSVA-upregulated genes were significant only for RSVA, compared with 79 of 144 for IAV, five of 11 for hMPV, and 10 of 60 for RSVB (Figure 4c). The RSVA-specific ranking was led by *VEGFA*, *PLAU*, *IL1B*, *RB1CC1*, and *SSR1*, which distinguished the broad late RSVA response from the interferon-centered genes shared among infections. This structure was consistent across other folds (Supplementary Figures S9–S12). The RSVA-specific group contained 106 to 125 genes in every fold and remained larger than the IAV-specific group, which contained 73 to 85 genes. The dominant virus-associated upregulated response therefore shifted from IAV at 24 h to RSVA at 72 h.

Although RSVB retained 53 to 78 upregulated genes at 72 h, only 7 to 12 were specific to RSVB across folds. Larger groups of RSVB-responsive genes were shared with RSVA or with both IAV and RSVA. The exact RSVA-RSVB intersection contained 18 to 30 genes, while the IAV-RSVA-RSVB intersection contained 12 to 27 genes. The late RSVB response was therefore composed primarily of genes that were also induced by RSVA and, in many cases, IAV. This finding is consistent with the shift toward the interferon-stimulated profile shown for RSVB in Figure 2b.

In contrast to the stable early common response, exact four-virus upregulated membership became less consistent at 72 h. No gene belonged to this intersection in outer fold 1, and its size ranged from zero to 10 genes across the five folds. *IRF9* and *IFI44* were the most recurrent late common candidates, but neither retained exact four-virus membership in every fold. Thus, the common early response centered on *RSAD2*, *OAS2*, and *STAT1* became less uniform as the four infections progressed, while RSVA developed a substantially larger virus-associated program.

The 72-h downregulated response showed a different form of convergence and was dominated by the exact IAV-hMPV intersection. This group contained 157 genes in outer fold 1 (Figure 4d) and ranged from 144 to 185 genes across all five folds, which made it the largest downregulated intersection in every fold. Substantial additional components included 93 to 120 hMPV-specific genes, 94 to 141 RSVA-specific genes, and 74 to 87 genes shared by IAV and RSVA. These patterns show that the broad late transcriptional decreases observed in Figure 3 were not distributed uniformly among the viruses. Instead, a major component was shared specifically by IAV and hMPV, while RSVA contributed both a large virus-specific group and a separate overlap with IAV.

RSVB again contributed little to the downregulated response. Only zero to two genes were specific to RSVB at 72 h, and most intersections containing RSVB were small. In outer fold 1, six genes were downregulated by all four viruses, with *ITGB2*, *IFNW1*, *CCL17*, *CD36*, and *EPHX2* representing the five highest-ranked displayed candidates. However, this intersection was absent in three of the remaining four folds and contained only one gene in outer fold 3. The fold-specific six-gene intersection therefore did not represent a stable common downregulated response.

### 4.3 HCNA reduces statistically prioritized genes to module representatives

**FIGURE 5:**
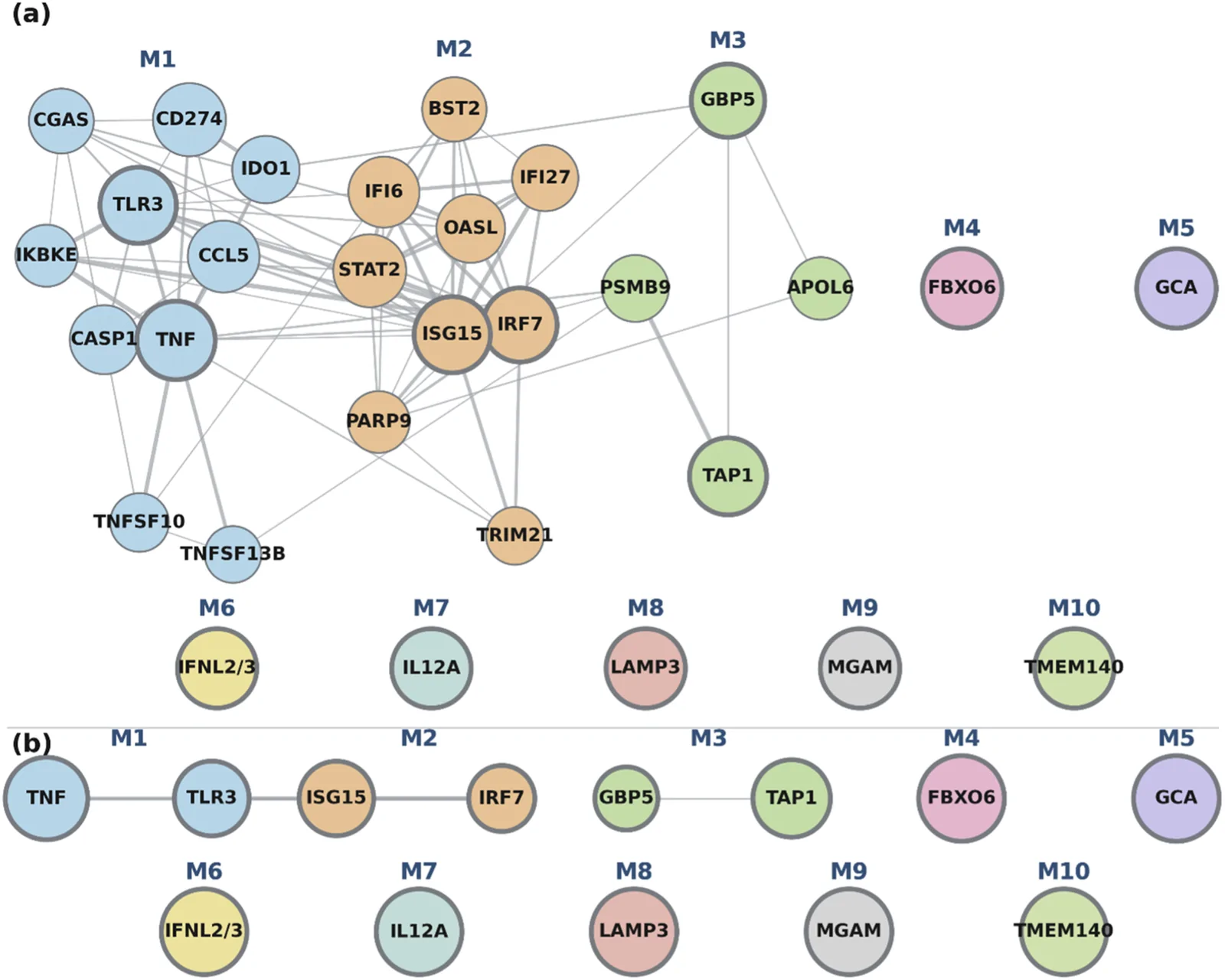
Illustrative HCNA module detection and q -based representative selection for the IAV-specific response. The IAV-specific candidate group at 24 h from outer fold one is shown as an example of the HCNA procedure. Inner cross-validation selected K=30 genes to enter the STRING network and a maximum of q=2 representatives per module. **(a)** STRING-induced network containing the 30 candidates. Node colors identify the 10 detected modules, M1 through M10. Node size is proportional to the within-module hub score, edge width reflects the STRING interaction score, and thick node borders identify genes retained by q selection. The connected component was divided into three multi-gene modules, while genes without qualifying connections were retained as singleton modules. **(b)** Subnetwork containing the 13 HCNA-selected representatives. Up to two nonredundant representatives were retained from each multi-gene module, while each singleton module contributed its only gene.

The HCNA procedure was illustrated using the 24-h IAV-specific group from outer fold 1 (Figure 5). Inner cross-validation selected K = 30 MGSR-ranked genes for STRING analysis (Figure 5a) and allowed up to q = 2 representatives per module (Figure 5b). HCNA organized these candidates into three multi-gene modules and seven singleton modules, ranked genes relative to the structure of their corresponding modules, and reduced the 30-gene candidate set to 13 representatives. This selection preserved every detected module and both expression directions while limiting repeated selection from the larger, densely connected modules.

This example demonstrates how HCNA reduces network redundancy without excluding candidates from smaller or disconnected biological components. The same module detection, within-module hub ranking, expression-correlation control, and representative-selection procedure was applied separately to every MGSR group at 24 and 72 h within each training partition.

### 4.4 Nested cross-validation demonstrates improved generalization

**FIGURE 6:**
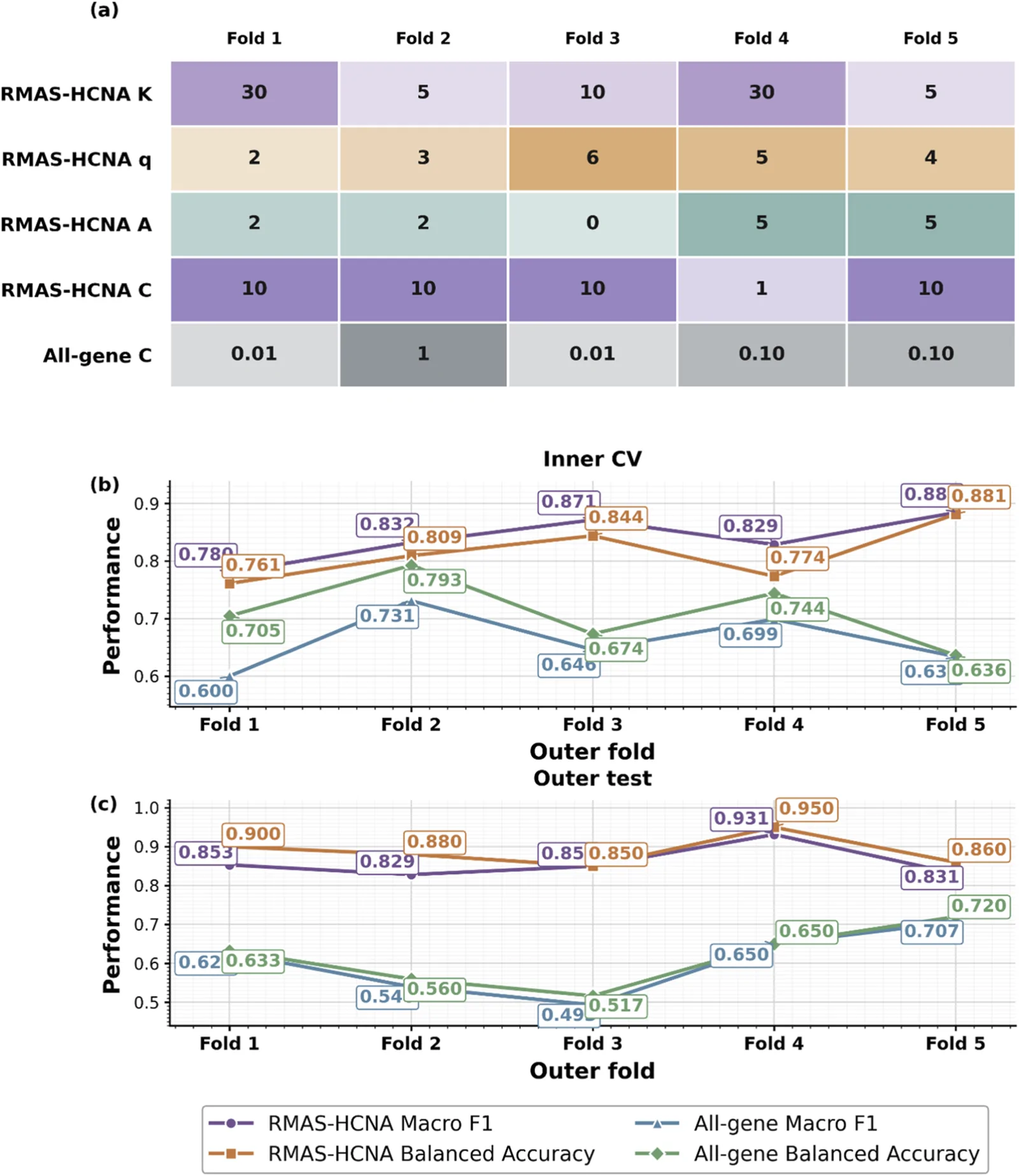
Nested cross-validation hyperparameter selection and predictive performance of RMAS-HCNA *versus* the all-gene baseline. **(a)** Heatmap of the hyperparameters selected within each outer fold. For RMAS-HCNA, these include the number of genes entering STRING $\left(K\right)$ , the maximum number of representatives retained per module $\left(q\right)$ , the number of direction-aware RMAS anchors $\left(A\right)$ , and the logistic-regression regularization parameter $\left(C\right)$ . The selected C values for the all-gene comparator are also shown. **(b)** Inner cross-validation performance for each outer fold, reported as Macro F1 and balanced accuracy for RMAS-HCNA and the all-gene comparator. **(c)** Performance on the untouched outer-test fold, reported using the same metrics. RMAS-HCNA was trained using the fold-specific training-only feature-selection pipeline, whereas the all-gene comparator used the full measured gene set under the same nested cross-validation structure.

Figure 6 evaluates whether the gene panels derived through RMAS, MGSR, and HCNA retained predictive information in samples that were excluded from both feature selection and model optimization. Inner cross-validation selected K : the number of MGSR-ranked genes entering STRING, q : the maximum number of representatives retained per module, the number of direction-aware A : RMAS anchors, and C : the logistic-regression regularization parameter independently within each outer-training partition (Figure 6a). The selected values differed across outer folds, with K ranging from 5 to 30, q from two to 6, and the number of RMAS anchors from 0 to 5. Thus, the final configuration was determined by the available training samples rather than imposed as a single fixed parameter combination across all evaluations.

RMAS-HCNA consistently outperformed the all-gene comparator during inner cross-validation (Figure 6b). Across the five outer folds, its mean inner-validation Macro F1 ranged from 0.780 to 0.884, while balanced accuracy ranged from 0.761 to 0.881. The corresponding all-gene values ranged from 0.600 to 0.731 for Macro F1 and from 0.636 to 0.793 for balanced accuracy. The advantage of RMAS-HCNA became clearer when the selected configurations were applied to the untouched outer-test samples (Figure 6c). RMAS-HCNA achieved outer-test Macro F1 values of 0.853, 0.829, 0.850, 0.931, and 0.831 across folds one through 5, respectively. The corresponding balanced accuracies were 0.900, 0.880, 0.850, 0.950, and 0.860. In contrast, the all-gene model produced Macro F1 values of 0.628, 0.540, 0.493, 0.650, and 0.707 and balanced accuracies of 0.633, 0.560, 0.517, 0.650, and 0.720. RMAS-HCNA therefore outperformed the all-gene comparator in every outer fold for both evaluation measures.

**FIGURE 7:**
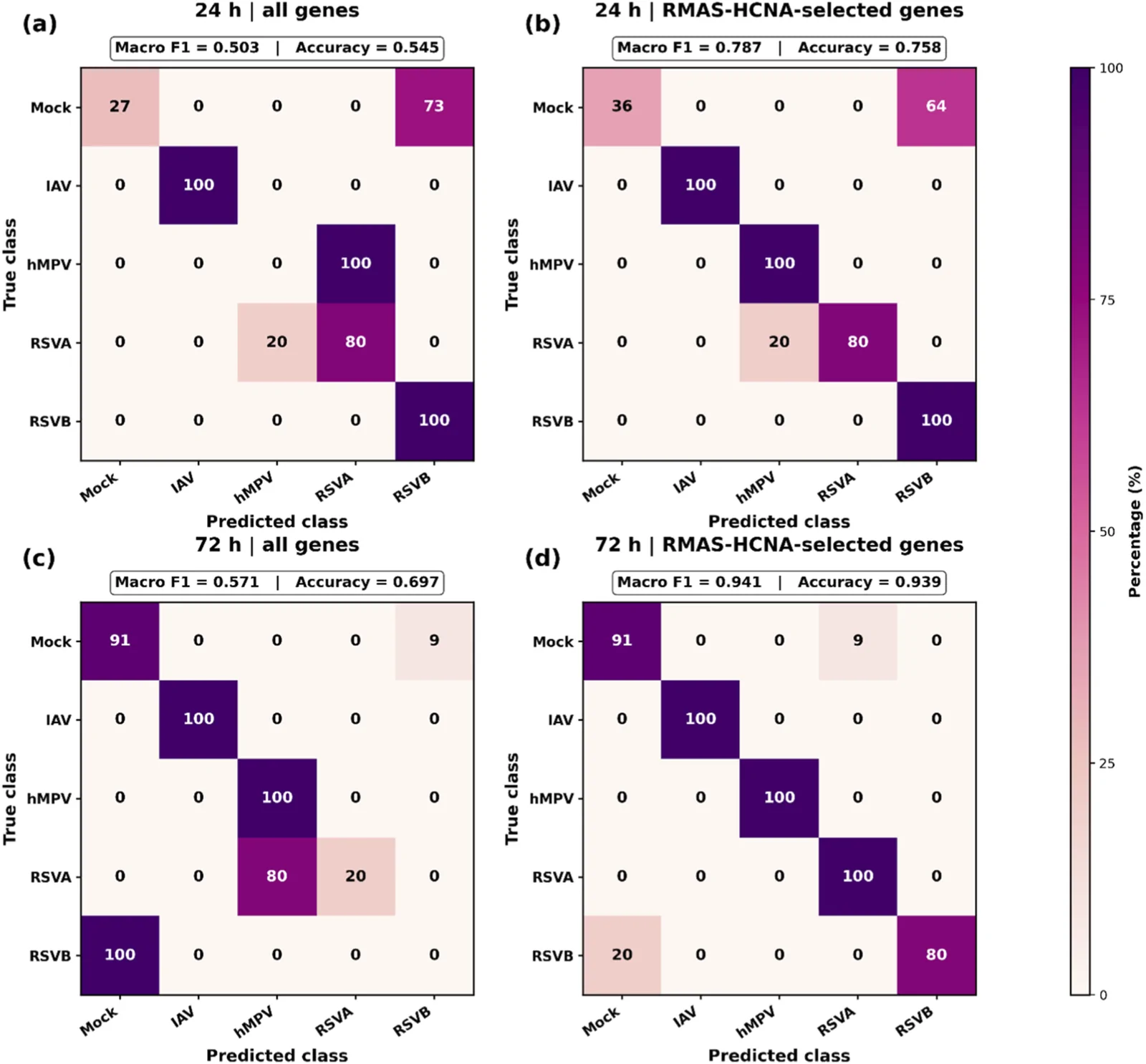
Time-specific out-of-fold classification performance of the all-gene and RMAS-HCNA models. Row-normalized confusion matrices show pooled outer-test predictions separately at 24 and 72 h. Each sample received one prediction from the outer-fold model for which it was held out. Panels show **(a)** the 24-h all-gene model, **(b)** the 24-h RMAS-HCNA-selected gene model, **(c)** the 72-h all-gene model, and **(d)** the 72-h RMAS-HCNA-selected gene model. Values within cells indicate the percentage of samples from each true class assigned to each predicted class. Macro F1 and overall accuracy are displayed above each matrix. Mock-IM and Mock-R were evaluated as a common Mock classification outcome, while their source identities were retained during partitioning and training-only harmonization.

The pooled out-of-fold predictions were next separated by time point to determine which class boundaries accounted for the performance differences (Figure 7). At 24 h, the all-gene model achieved a Macro F1 of 0.503, an accuracy of 0.545, and a balanced accuracy of 0.615 (Figure 7a). IAV and RSVB were classified correctly in all samples, and 80% of RSVA samples were classified correctly. However, every hMPV sample was assigned to RSVA, while only 27% of Mock samples were correctly classified and the remaining 73% were assigned to RSVB.

The RMAS-HCNA-selected gene model increased the 24-h Macro F1 to 0.787, accuracy to 0.758, and balanced accuracy to 0.833 (Figure 7b). All IAV, hMPV, and RSVB samples were classified correctly, while 80% of RSVA samples were correctly assigned. The selected panels therefore resolved the complete hMPV-to-RSVA misclassification observed with the all-gene model. The main remaining difficulty was the separation of Mock from RSVB, with 36% of Mock samples classified correctly and 64% assigned to RSVB.

The distinction between the two approaches was even greater at 72 h. The all-gene model achieved a Macro F1 of 0.571, an accuracy of 0.697, and a balanced accuracy of 0.622 (Figure 7c). Although IAV and hMPV were classified correctly in all samples and Mock recall reached 91%, only 20% of RSVA samples were correctly identified, with the remaining 80% assigned to hMPV. In addition, every RSVB sample was classified as Mock.

With the RMAS-HCNA-selected genes, the 72-h Macro F1 increased to 0.941, accuracy to 0.939, and balanced accuracy to 0.942 (Figure 7d). IAV, hMPV, and RSVA achieved complete recall. Mock recall remained at 91%, with one Mock sample assigned to RSVA, while four of the five RSVB samples were correctly classified and one was assigned to Mock. RMAS-HCNA therefore resolved the major hMPV-RSVA confusion and recovered the distinction between RSVB and Mock that was absent from the 72-h all-gene projection. The stronger 72-h performance was consistent with the broader and more differentiated late transcriptional responses identified in Figure 2–4.

**FIGURE 8:**
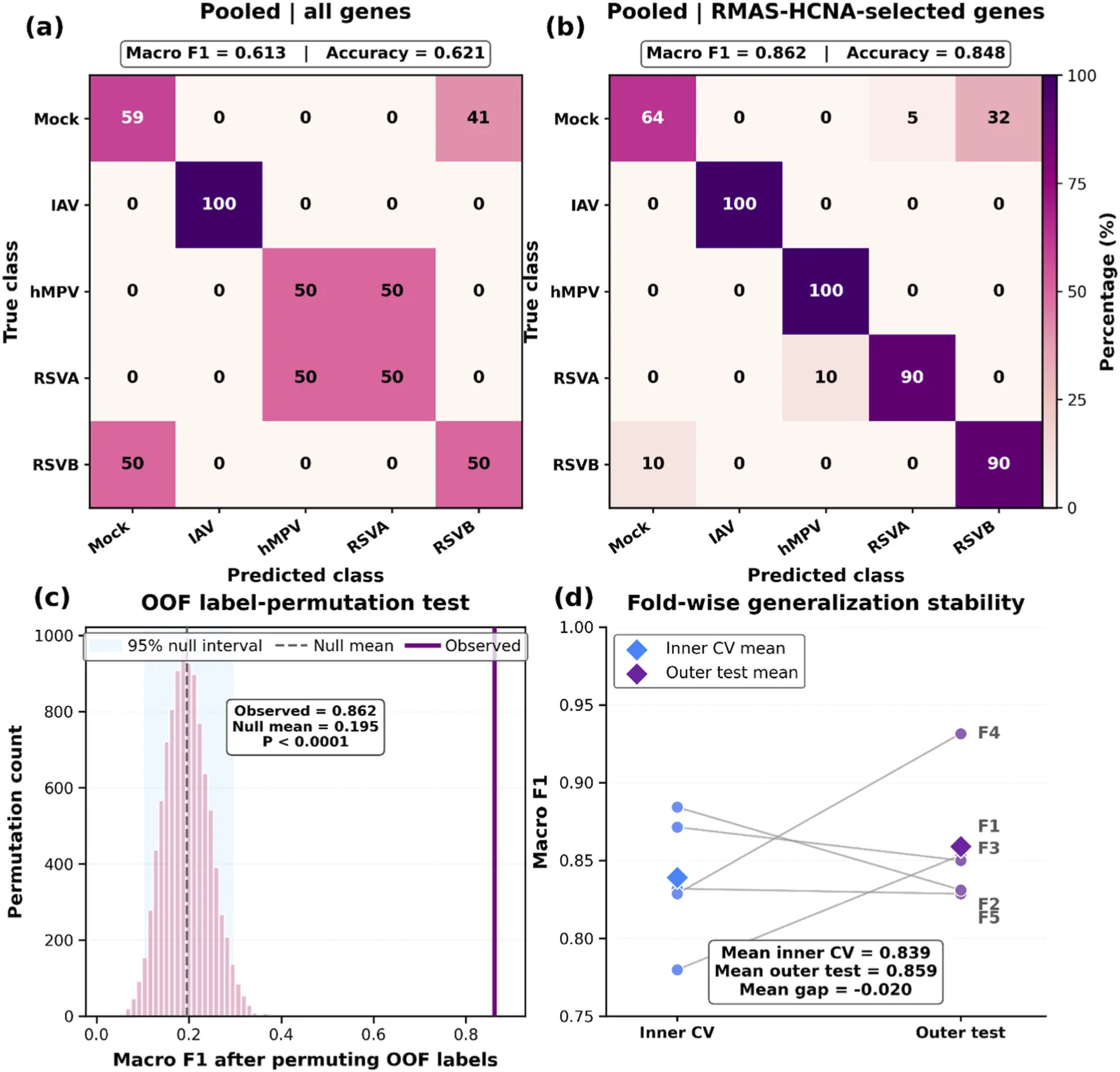
Pooled out-of-fold classification, label-permutation analysis, and fold-wise generalization stability. Row-normalized confusion matrices show predictions pooled across 24 and 72 h for **(a)** the all-gene model and **(b)** the RMAS-HCNA-selected gene model. Each sample received one prediction from its held-out outer fold. Values within cells indicate class-specific prediction percentages, while Macro F1 and overall accuracy are displayed above each matrix. **(c)** Null distribution of Macro F1 values generated from 10,000 permutations of the true-label alignment against the fixed RMAS-HCNA out-of-fold predictions. The dashed line indicates the null mean, the shaded region indicates the 95% null interval, and the solid line indicates the observed Macro F1. **(d)** Comparison of the selected configuration’s mean inner cross-validation Macro F1 with its corresponding outer-test Macro F1 for each outer fold. Gray lines connect values from the same fold. Diamond markers show the means across folds, and the generalization gap is defined as inner cross-validation Macro F1 minus outer-test Macro F1.

When predictions from both time points were pooled, the all-gene model achieved a Macro F1 of 0.613, an accuracy of 0.621, and a balanced accuracy of 0.618 (Figure 8a). IAV was classified correctly in all samples, but recall was only 59% for Mock and 50% for each of hMPV, RSVA, and RSVB. Its main errors consisted of reciprocal confusion between hMPV and RSVA and between Mock and RSVB.

RMAS-HCNA increased the pooled Macro F1 to 0.862, accuracy to 0.848, and balanced accuracy to 0.887 (Figure 8b). This corresponded to 56 correct predictions among the 66 samples, compared with 41 for the all-gene model. IAV and hMPV achieved 100% recall, while RSVA and RSVB each achieved 90% recall. Mock remained the most difficult class, with 64% recall; 32% of Mock samples were assigned to RSVB and 5% were assigned to RSVA. The remaining infection-class errors consisted of one RSVA sample assigned to hMPV and one RSVB sample assigned to Mock. Thus, the primary residual limitation was the separation of Mock and RSVB rather than discrimination among the four viral infections.

Two complementary analyses were used to evaluate whether the high out-of-fold performance could be explained by chance agreement or systematic inner-loop overfitting. First, the pooled RMAS-HCNA Macro F1 of 0.862 was compared with a null distribution generated from 10,000 random permutations of the alignment between the true labels and the fixed out-of-fold predictions (Figure 8c). The null distribution had a mean of 0.195 and a 95% interval of 0.105–0.295, while none of the 10,000 permutations reached the observed value, which produced P<0.0001 . The observed agreement between the predictions and the true classes was therefore substantially greater than expected under randomized label alignment.

Second, Figure 8d directly compared the mean inner-validation Macro F1 used for hyperparameter selection with the corresponding outer-test Macro F1 for each fold. The mean inner-validation value was 0.839, while the mean outer-test value was 0.859, which produced an inner-minus-outer generalization gap of −0.020 . Fold-specific gaps ranged from −0.103 to 0.053, with some outer-test folds performing better than their corresponding inner-validation estimates and others performing slightly worse. There was therefore no systematic decrease from inner validation to outer testing.

Taken together, Figures 8c,d provide complementary evidence against overfitting within the present dataset. Figure 8c shows that the pooled out-of-fold performance was far greater than expected by chance, while Figure 8d shows that performance did not systematically decline when the inner-selected configurations were applied to untouched outer-test samples. Because every outer-test sample was excluded from gene discovery, MGSR and HCNA selection, ComBat estimation, hyperparameter optimization, dimensionality reduction, and classifier fitting, the close agreement between inner-validation and outer-test performance indicates that model selection did not produce an optimistic inner-loop estimate that failed to generalize to unseen samples. However, these analyses cannot establish the complete absence of overfitting or confirm generalization beyond the current OTE datasets; independent validation in a matched external dataset remains necessary.

### 4.5 Twelve recurrent genes capture major biological-group structure

**FIGURE 9:**
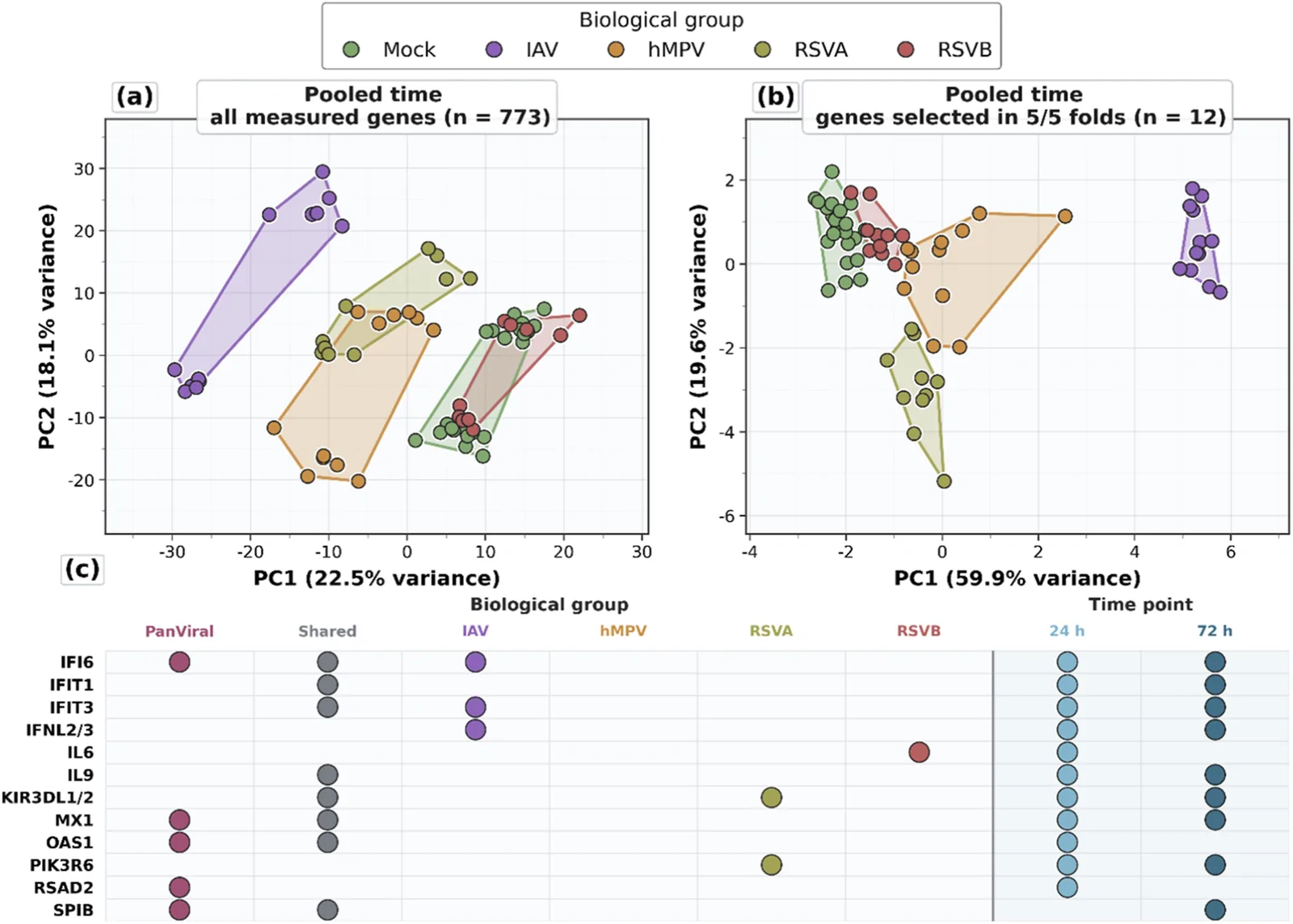
Pooled PCA and biological context of genes selected in all five outer folds. All 66 samples from 24 to 72 h were pooled after time-specific, Mock-anchored harmonization. Mock-IM and Mock-R were displayed as a common Mock biological group. **(a)** PCA using all 773 measured genes. PC1 and PC2 explained 22.5% and 18.1% of the variance, respectively. **(b)** PCA using the 12 genes selected in all five outer folds: IFI6, IFIT1, IFIT3, IFNL2/3, IL6, IL9, KIR3DL1/2, MX1, OAS1, PIK3R6, RSAD2, and SPIB. PC1 and PC2 explained 59.9% and 19.6% of the variance, respectively. Points represent individual samples, colors identify biological groups, and shaded polygons show the convex hull of each group. **(c)** Dot matrix showing the biological groups and time points associated with selection of each of the 12 genes across the five outer folds. A gene may appear under multiple biological groups or time points when it was selected from different contexts across folds. PanViral and Shared assignments reflect MGSR candidate groups, while IAV, hMPV, RSVA, and RSVB indicate virus-specific groups.

To examine the transcriptional structure retained by the most reproducibly selected genes, all 66 samples from 24 to 72 h were pooled after time-specific, Mock-anchored harmonization. PCA based on all 773 measured genes explained 22.5% of the variance through PC1 and 18.1% through PC2, for a combined 40.5% (Figure 9a). IAV occupied a region distinct from the other biological groups, although its broad distribution reflected substantial differences between the two time points. hMPV and RSVA showed considerable overlap, while Mock and RSVB occupied closely related regions. Thus, the dominant variance across the complete gene set did not clearly resolve several of the biological groups.

Twelve genes were included in the final RMAS-HCNA panel in all five outer folds: *IFI6*, *IFIT1*, *IFIT3*, *IFNL2/3*, *IL6*, *IL9*, *KIR3DL1/2*, *MX1*, *OAS1*, *PIK3R6*, *RSAD2*, and *SPIB*. When PCA was restricted to these genes, PC1 explained 59.9% of the variance and PC2 explained 19.6%, for a combined 79.5% (Figure 9b). The recurrent 12-gene panel therefore represented nearly twice as much of the total variation within the first two components as the complete 773-gene set.

The biological-group structure was also more clearly resolved with the 12 genes. IAV formed a compact and distinctly separated cluster along PC1. RSVA separated primarily along PC2 and occupied a region distinct from IAV, Mock, and RSVB. hMPV occupied an intermediate region but was more clearly distinguished from RSVA than in the all-gene projection. Mock and RSVB remained the most closely positioned groups and retained partial overlap, which was consistent with the residual Mock-RSVB confusion observed in the out-of-fold classifications. The 12-gene panel therefore strengthened separation among the principal viral responses while preserving the main class boundary that remained difficult for the classifier.

The cross-fold selection contexts showed that these genes did not arise from one virus, time point, or MGSR group (Figure 9c). Five genes, *IFI6*, *MX1*, *OAS1*, *RSAD2*, and *SPIB*, were selected from a PanViral group in at least one outer fold. Eight genes had Shared-group selection contexts: *IFI6*, *IFIT1*, *IFIT3*, *IL9*, *KIR3DL1/2*, *MX1*, *OAS1*, and *SPIB*. These genes represented recurrent cross-virus signals, with several belonging to the interferon-associated response identified in Figure 2–4.

The panel also retained virus-associated components that could help distinguish infections with overlapping antiviral responses. *IFI6*, *IFIT3*, and *IFNL2/3* were selected from IAV-specific groups, while *KIR3DL1/2* and *PIK3R6* had RSVA-specific selection contexts. *IL6* represented the RSVB-specific component and reflected the early RSVB response observed in Figure 2. No gene was selected from an hMPV-specific group in all five folds. Nevertheless, hMPV remained distinguishable in the PCA and achieved complete recall in the pooled out-of-fold predictions. Its separation therefore depended on the combined expression pattern of shared and other virus-associated genes rather than on a recurrent hMPV-specific marker.

The 12 genes also represented both stages of infection. Eight genes, *IFI6*, *IFIT1*, *IFIT3*, *IFNL2/3*, *IL9*, *KIR3DL1/2*, *MX1*, and *PIK3R6*, were selected in association with both 24- and 72-h responses. *IL6*, *OAS1*, and *RSAD2* were associated only with 24-h selection contexts, while *SPIB* was associated only with 72 h. The panel therefore combined genes that recurred across time with genes that preserved information specific to the early or late response.

### 4.6 Stable transcriptional responses reveal shared antiviral activity and distinct virus-specific functional trajectories

**FIGURE 10:**
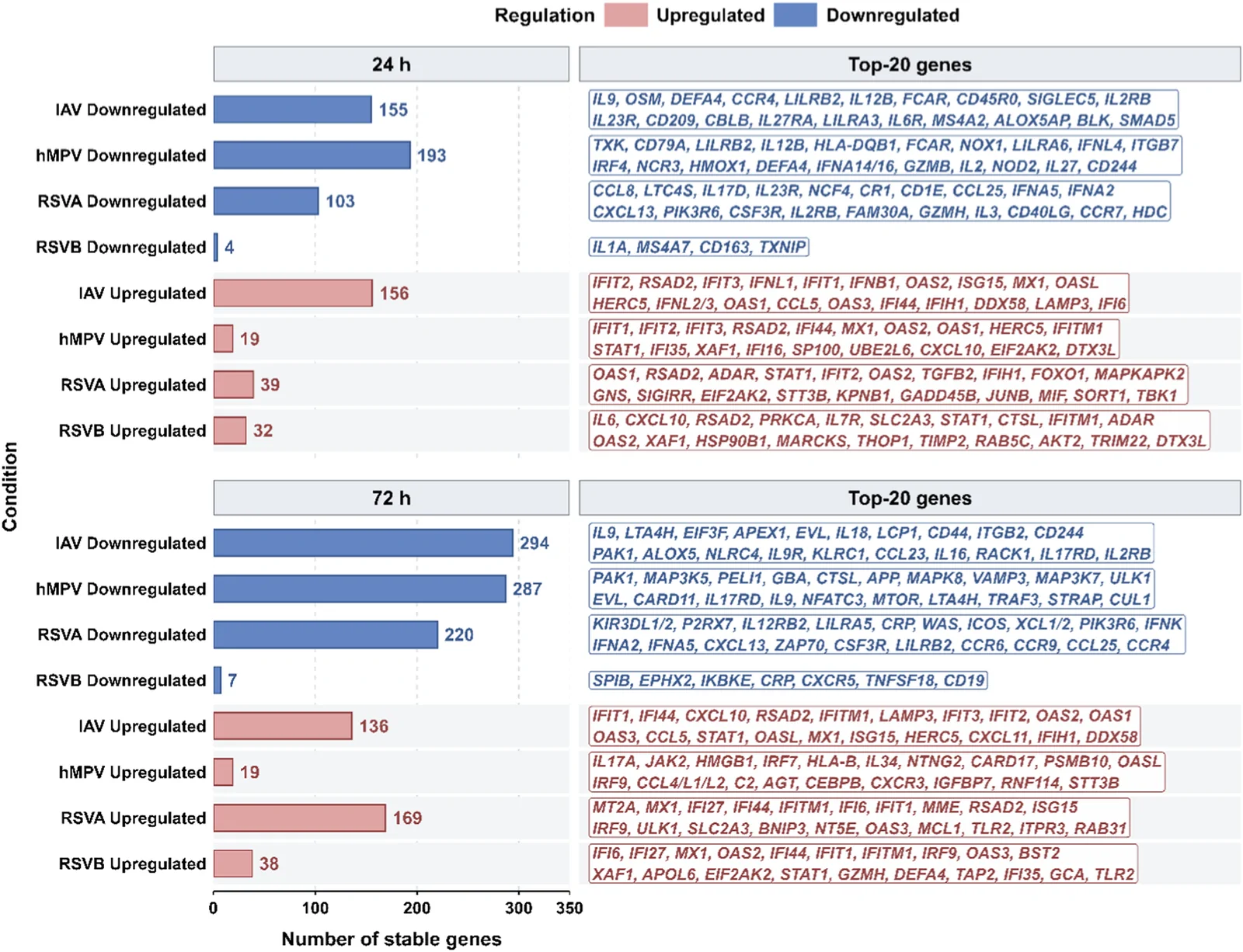
Stable gene counts and leading stable genes across viruses and time points. Bars show the numbers of stably upregulated and downregulated genes for IAV, hMPV, RSVA, and RSVB at 24 and 72 h. The adjacent panels list the 20 highest-ranked stable genes for each virus, time point, and direction. Genes were ranked first by outer-fold support and then by average RMAS across the folds in which they were significant. Bar labels show the number of stable genes in each group.

To identify biological responses that were reproducible across changes in outer-training composition, genes were considered stable when they were significant in the same direction in at least four of the five outer folds, with no significant response in the opposite direction. The numbers and leading examples of these stable genes differed substantially among viruses and time points (Figure 10).

At 24 h, IAV produced the broadest and most directionally balanced stable response, with 156 upregulated and 155 downregulated genes. In contrast, hMPV was strongly dominated by downregulation, with only 19 stably upregulated genes compared with 193 stably downregulated genes. RSVA also showed a downregulation-dominant early response, with 39 upregulated and 103 downregulated genes. RSVB produced the most restricted response, with 32 upregulated genes and only four downregulated genes. The total stable response therefore ranged from 311 genes for IAV to 36 genes for RSVB, with hMPV and RSVA occupying intermediate positions at 212 and 142 genes, respectively.

The leading upregulated genes revealed a recurrent early antiviral component despite these differences in response breadth. The 24-h IAV and hMPV rankings shared *IFIT1*, *IFIT2*, *IFIT3*, and *RSAD2*. RSVA also included *RSAD2* and *IFIT2*, together with *OAS1*, *ADAR*, and *STAT1*. RSVB was more distinct because its ranking was led by *IL6*, *CXCL10*, *RSAD2*, *PRKCA*, and *IL7R*. Thus, IAV, hMPV, and RSVA showed different versions of an interferon-associated response, whereas RSVB combined a smaller antiviral component with an inflammatory and signaling-associated profile.

By 72 h, the stable responses of IAV, hMPV, and particularly RSVA had expanded, but they followed different directional patterns. IAV contained 136 upregulated and 294 downregulated genes, which increased its total stable response from 311 to 430 genes. The number of hMPV-upregulated genes remained unchanged at 19, whereas its downregulated component increased from 193 to 287 genes. RSVA showed the strongest temporal expansion. Its upregulated set increased from 39 to 169 genes, while its downregulated set increased from 103 to 220 genes, which raised its total stable response from 142 to 389 genes. RSVB remained comparatively restricted, with 38 upregulated and seven downregulated genes.

The leading 72-h genes also showed that the viruses followed different temporal trajectories. IAV retained a strong antiviral profile led by *IFIT1*, *IFI44*, *CXCL10*, *RSAD2*, and *IFITM1*. The hMPV ranking shifted away from the early IFIT-centered response toward *IL17A*, *JAK2*, *HMGB1*, *IRF7*, and *HLA-B*. RSVA and RSVB became more similar to one another because both rankings included *MX1*, *IFI27*, and *IFI44*. RSVA additionally contained *MT2A* and *IFITM1*, whereas RSVB contained *IFI6* and *OAS2*. In contrast to the recurrent overlap among upregulated genes, the leading downregulated genes remained largely distinct among viruses at both time points.

**FIGURE 11:**
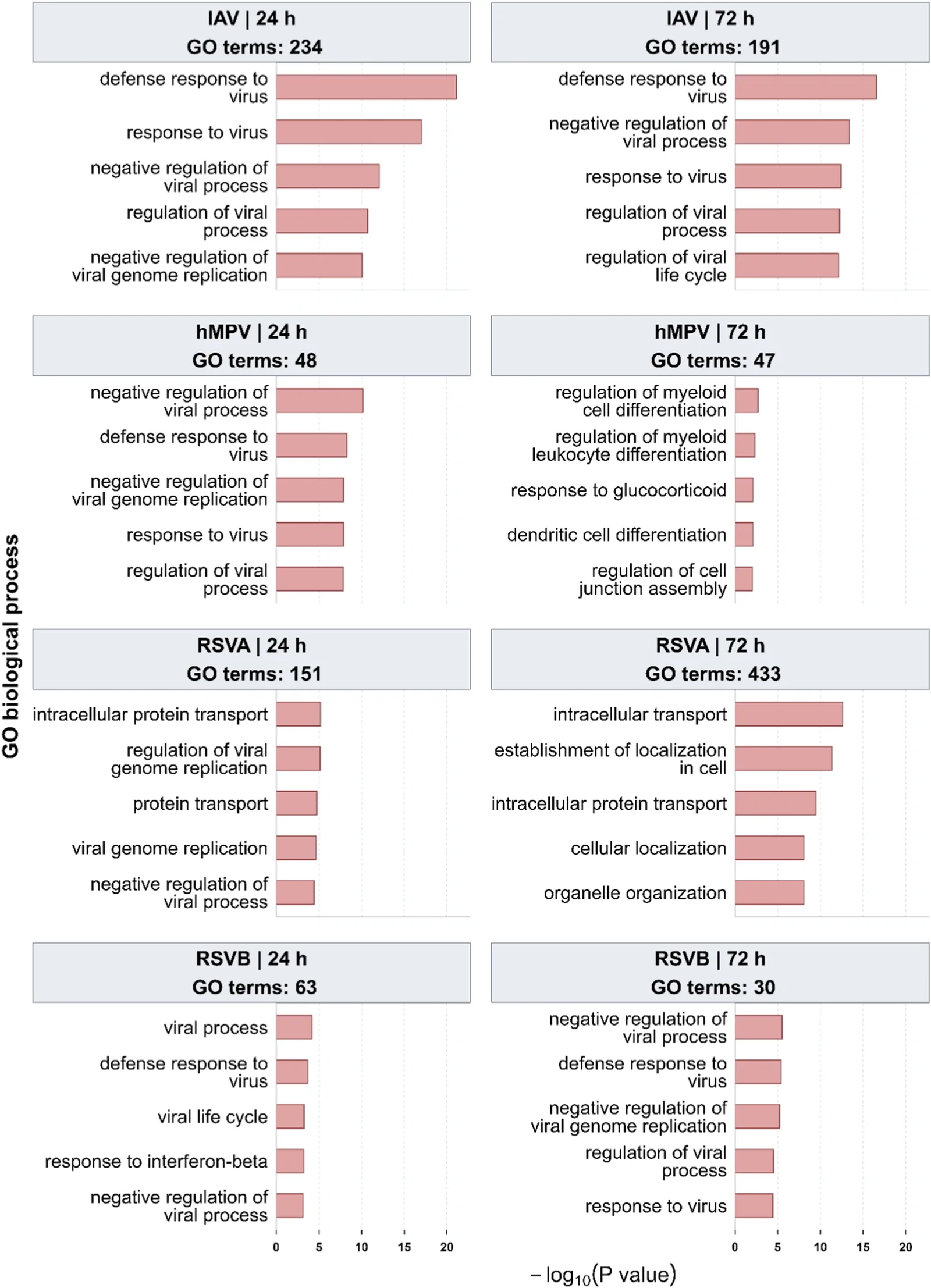
Leading GO Biological Process terms enriched among stably upregulated genes. The five highest-ranked significant GO Biological Process terms are shown for IAV, hMPV, RSVA, and RSVB at 24 and 72 h. The ribbon above each panel reports the total number of significant GO terms for that virus and time point. Bar length represents $-\log_{10}\left(P‐value\right)$ . Terms were ranked by significance, followed by adjusted significance, fold enrichment, overlap-gene count, and GO identifier.

GO Biological Process enrichment among the stable upregulated genes extended these gene-level observations by identifying the biological functions associated with each response (Figure 11). At 24 h, IAV had 234 significant GO terms, RSVA had 151, RSVB had 63, and hMPV had 48. The leading IAV and hMPV terms were closely related and were centered on defense response to virus, response to virus, regulation of viral processes, and restriction of viral genome replication. Thus, the small hMPV-upregulated set and the much larger IAV-upregulated set converged on a similar core antiviral function despite their difference in size.

RSVB also showed an antiviral early profile, which included viral process, defense response to virus, viral life cycle, response to interferon-beta, and negative regulation of viral processes. RSVA shared some of this antiviral organization, but its leading terms placed greater emphasis on intracellular protein transport and protein transport, together with regulation of viral genome replication. The early upregulated responses therefore shared virus-response functions, but RSVA showed an additional transport-associated component and RSVB retained a more restricted set of viral and interferon-related processes.

These functional patterns diverged further at 72 h. IAV retained a broad antiviral profile, with 191 significant terms led by defense response to virus, response to virus, and regulation of viral processes. RSVB also remained mainly antiviral, but the number of significant terms decreased from 63 to 30. Its leading terms included negative regulation of viral processes, defense response to virus, and regulation of viral genome replication. IAV and RSVB therefore preserved a similar general antiviral organization, although the IAV enrichment was substantially broader.

The hMPV-upregulated profile followed a different late trajectory. The number of significant terms remained nearly unchanged at 47, but the leading functions shifted from direct antiviral responses to regulation of myeloid cell differentiation, regulation of myeloid leukocyte differentiation, response to glucocorticoid, dendritic cell differentiation, and regulation of cell-junction assembly. RSVA showed the largest functional expansion. Its number of enriched terms increased from 151 at 24 h to 433 at 72 h, and the leading processes were intracellular transport, establishment of localization in cell, intracellular protein transport, cellular localization, and organelle organization. The late RSVA response therefore expanded beyond its early antiviral and transport-associated profile into a broad program of intracellular trafficking, localization, and cellular organization.

Enrichment among the stable downregulated genes provided a complementary view of the four responses (Supplementary Figure S13). At 24 h, IAV, hMPV, and RSVA had similar numbers of significant terms, with 58, 43, and 53, respectively, whereas RSVB had only five. However, the dominant functions were different. The IAV terms included granulocyte-macrophage colony-stimulating factor production, antigen receptor-mediated signaling, and glycerophospholipid metabolism. The hMPV profile was centered on actomyosin structure, cellular-component size, actin-filament organization, and supramolecular-fiber organization. RSVA was distinguished by natural killer cell activation, humoral immune response, cell killing, and leukocyte-mediated cytotoxicity. The limited RSVB terms involved acute-phase response, cell division, acute inflammatory response, and response to metal ions.

At 72 h, the functional breadth of the downregulated responses increased for IAV, hMPV, and RSVA. IAV increased from 58 to 147 significant terms, with leading functions related to organophosphate metabolism, cellular localization, vesicle-mediated transport, and cell-projection assembly. RSVA increased from 53 to 80 terms and retained an immune-centered profile that included cellular defense response, cell killing, immune effector processes, regulation of cytosolic calcium concentration, and chemokine-mediated signaling.

The largest late expansion occurred in hMPV, for which the number of significant downregulated terms increased from 43 to 310. The leading terms included macroautophagy, cellular response to stress, protein localization to organelles, intracellular transport, and catabolic processes. This result identified broad suppression of stress-response, trafficking, and degradative functions as a major feature of the late hMPV response. RSVB remained limited to six terms, which were associated primarily with lipid and nutrient storage, lipid localization, and B-cell activation. Thus, the upregulated analyses identified a partially shared antiviral foundation, whereas the downregulated analyses revealed more distinct virus-associated functions.

To distinguish general enrichment patterns from processes detected for only one infection, exact virus-unique GO terms were identified separately within each time point and expression direction. An exact unique term was significant for one virus but not for the other three viruses within the corresponding comparison. The 24-h exact unique analyses are presented for upregulated and downregulated genes in Supplementary Figures S14 and S15, respectively.

Among upregulated genes at 24 h, IAV had 139 exact unique terms, RSVA had 79, RSVB had 22, and hMPV had none (Supplementary Figure S14). The IAV-unique profile extended beyond the antiviral terms shared with other viruses and included regulation of cytokine-mediated signaling, necroptotic process, response to cytokine, response to peptide, and positive regulation of innate immune response. These processes were supported by broad and recurrent gene groups that included *ISG15*, *OAS1*, *OAS3*, *CCL5*, *IRF7*, *ZBP1*, *TNF*, *MLKL*, *TLR3*, *IFNL1*, *IFIT1*, and *IFNB1*.

The 24-h RSVA-unique response was organized around intracellular transport and RNA-related functions. Its leading terms were intracellular protein transport, intracellular transport, nucleocytoplasmic transport, mRNA metabolic process, and nuclear transport. Recurrent supporting genes included *ADAR*, *KPNB1*, *TBK1*, *MAPK14*, *TXN*, *TRAM1*, and *BCL3*. RSVB-unique terms included response to endoplasmic-reticulum stress, adenylate cyclase-activating G protein-coupled receptor signaling, regulation of translation, positive regulation of type 2 immune response, and CD4-positive alpha-beta T-cell cytokine production. These smaller term sets were supported by genes such as *IL6*, *CXCL10*, *PRKCA*, *RSAD2*, *AKT2*, and *APP*. The absence of an exact hMPV-unique upregulated term indicated that its limited early induced response was functionally contained within processes also enriched for at least one of the other viruses.

The exact unique downregulated profiles at 24 h were more evenly distributed, with 54 terms for IAV, 37 for hMPV, 50 for RSVA, and five for RSVB (Supplementary Figure S15). IAV-unique terms involved granulocyte-macrophage colony-stimulating factor production, antigen receptor-mediated signaling, and glycerophospholipid metabolism. The hMPV-unique profile was centered on actomyosin organization, regulation of cellular-component size, actin-filament organization, supramolecular-fiber organization, and cell-matrix adhesion. RSVA-unique terms emphasized natural killer cell activation, humoral immune response, cell killing, and leukocyte-mediated cytotoxicity. The five RSVB-unique terms were supported primarily by *IL1A*, *CD163*, and *TXNIP*, which was consistent with the small number of stable downregulated RSVB genes.

**FIGURE 12:**
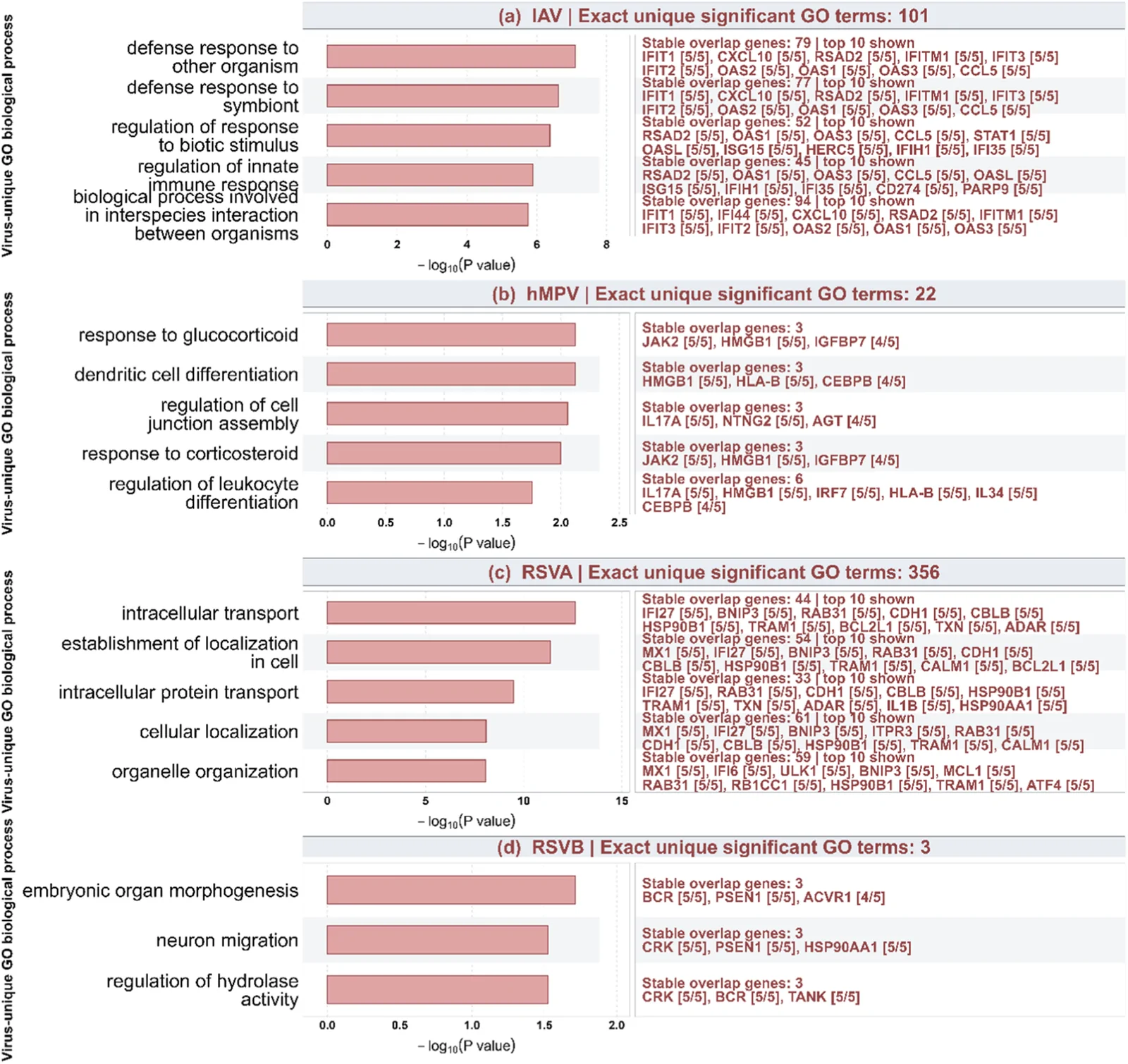
Exact virus-unique GO Biological Process terms among stably upregulated genes at 72 h. Panels show **(a)** IAV, **(b)** hMPV, **(c)** RSVA, and **(d)** RSVB. An exact virus-unique term was significant for only one virus within the 72-h upregulated comparison. The panel ribbon reports the total number of exact unique terms for each virus, and bars show the five highest-ranked available terms. Text on the right reports the total number of stable overlap genes and lists up to the 10 highest-ranked genes. Values in brackets indicate the number of outer folds supporting each gene.

The strongest virus-specific divergence among upregulated biological processes occurred at 72 h (Figure 12). RSVA had 356 exact unique terms, compared with 101 for IAV, 22 for hMPV, and only three for RSVB. IAV retained a broad virus-defense program, with leading unique terms that included defense response to other organisms, defense response to symbionts, regulation of response to biotic stimulus, regulation of innate immune response, and biological processes involved in interspecies interactions. These terms were supported by recurrent antiviral genes, including *IFIT1*, *CXCL10*, *RSAD2*, *IFITM1*, *IFIT3*, *IFIT2*, *OAS1*, *OAS2*, *OAS3*, *MX1*, and *ISG15*.

The 22 hMPV-unique terms represented a smaller but distinct late response. The leading processes included response to glucocorticoid, response to corticosteroid, dendritic cell differentiation, regulation of cell-junction assembly, and regulation of leukocyte differentiation. These terms were supported by relatively small stable-gene groups that included *JAK2*, *HMGB1*, *IGFBP7*, *HLA-B*, *CEBPB*, *IL17A*, *IRF7*, and *IL34*. This functional profile was consistent with the shift in the leading hMPV genes from an early IFIT-centered response toward differentiation, stress-related, and cellular-organization signals.

The RSVA-unique profile was both the largest and the most functionally expansive at 72 h. Its leading terms were intracellular transport, establishment of localization in cell, intracellular protein transport, cellular localization, and organelle organization. These processes were supported by broad, overlapping stable-gene sets that included *IFI27*, *MX1*, *BNIP3*, *RAB31*, *CDH1*, *CBLB*, *HSP90B1*, *TRAM1*, *BCL2L1*, *TXN*, *ADAR*, *IL1B*, and *RB1CC1*. The increase from 79 exact unique upregulated terms at 24 h to 356 at 72 h showed that the late RSVA response developed a broad functional identity centered on intracellular trafficking, localization, and organelle organization.

RSVB followed the opposite pattern. Its exact unique upregulated terms decreased from 22 at 24 h to only three at 72 h: embryonic organ morphogenesis, neuron migration, and regulation of hydrolase activity. Each term was supported by only three stable genes. The limited number and small supporting gene sets indicated that most of the late RSVB upregulated functions were also observed for at least one other virus rather than forming an extensive RSVB-specific program.

The exact unique downregulated profiles also diverged further at 72 h (Supplementary Figure S16). IAV had 76 unique terms, hMPV had 243, RSVA had 75, and RSVB retained five. The IAV profile shifted toward regulation of cell-projection assembly, protein-complex assembly, cellular-component biogenesis, and cytoskeleton organization. The hMPV profile showed the largest expansion and was dominated by cellular response to stress, protein localization to organelles, catabolic processes, intracellular protein transport, and primary metabolism. RSVA remained centered on immune-associated functions, including cellular defense response, regulation of cytosolic calcium concentration, immune effector processes, chemokine-mediated signaling, and response to chemokines. The five RSVB-unique terms involved lipid storage, nutrient storage, regulation of lipid localization, lipid localization, and B-cell differentiation.

## 5 Discussion

This study shows that the airway response to respiratory-virus infection is organized around a common antiviral foundation, but the breadth, timing, direction, and functional composition of that response differ substantially among IAV, hMPV, RSVA, and RSVB. The shared component was most evident among upregulated genes associated with interferon signaling and antiviral defense. However, this common response accounted for only part of the transcriptional structure. Each virus also produced a distinct pattern of gene induction and repression that became increasingly apparent over time. Taken together, the gene-level, cross-virus, network, classification, and Gene Ontology analyses describe four related but clearly distinguishable airway responses rather than variations of one uniform antiviral program.

The most consistent similarity among the four infections was a compact interferon-associated response. At the earlier time point 24 hpi, this shared foundation was centered on *RSAD2*, *OAS2*, *STAT1*, and IFIT-family genes. At the later time point 72 hpi, the recurrent response shifted toward *IFI44*, *IFI27*, *MX1*, and other interferon-stimulated effectors. This temporal change indicates that the common host response was itself dynamic. The early response emphasized viral sensing, interferon signaling, and initial antiviral activation, whereas the later response placed greater emphasis on downstream effector genes. The identification of a common antiviral component across all four infections supports the presence of a conserved airway defense program, but the small size of the exact four-virus intersections also shows that this program represented only a limited part of the complete response.

The asymmetry between upregulated and downregulated genes was central to the biological comparison. Upregulated responses repeatedly converged on a relatively small set of interferon-associated genes, while downregulated responses were much more distinct among viruses. This pattern was evident both in the leading gene rankings and in the exact cross-virus intersections. A reproducible four-virus upregulated group was present early, whereas no similarly stable common downregulated group emerged. Thus, the four viruses appeared to recruit a partially conserved antiviral response while producing different patterns of transcriptional reduction. These reductions distinguished the infections through their associations with immune regulation, cellular structure, stress responses, metabolism, transport, and intracellular organization.

IAV produced the broadest early response and maintained a strong antiviral identity throughout the experiment. Its upregulated profile remained centered on IFIT, IFI, OAS, MX, *RSAD2*, and chemokine-associated genes, which indicates that antiviral signaling persisted as the infection progressed. At the same time, the response became increasingly weighted toward downregulation. The later IAV profile therefore combined sustained antiviral induction with broader reductions involving metabolic processes, vesicle-mediated transport, cellular localization, cytoskeletal organization, and cell-projection assembly. This combination suggests that the IAV response extended beyond antiviral defense into wider cellular reorganization.

The large IAV-specific upregulated component further distinguished this infection from the other viruses. Although IAV shared a common interferon foundation with hMPV, RSVA, and RSVB, many induced genes reached significance only for IAV. The corresponding biological processes included cytokine-related signaling, innate immune regulation, responses to biological stimuli, and defense-associated functions. IAV therefore generated both the shared antiviral response and an extensive surrounding program that remained identifiable as IAV associated. This breadth also explains why IAV was the most consistently separated group in the multivariate and classification analyses.

hMPV followed a very different transcriptional trajectory. Its upregulated component remained small at both time points, yet the leading early genes overlapped extensively with the IAV antiviral response. Nearly all early hMPV-upregulated genes were also supported by one or more of the other viruses, and no exact hMPV-unique upregulated biological process was detected at 24 h. The early hMPV response therefore represented a limited version of the common antiviral program rather than a broad hMPV-specific induction pattern.

The defining feature of hMPV was the extent and functional organization of its downregulated response. At 24 h, these decreases were associated with actomyosin organization, actin-filament structure, cellular-component size, supramolecular fibers, and cell-matrix relationships. By 72 h, the downregulated program had expanded considerably and included stress responses, macroautophagy, intracellular transport, protein localization to organelles, catabolic processes, and primary metabolism. This progression suggests that the later hMPV response involved widespread changes in cellular maintenance, trafficking, degradation, and stress-associated functions.

The hMPV-upregulated response also changed in composition over time. The early IFIT-centered profile gave way to genes and biological processes related to differentiation, glucocorticoid and corticosteroid responses, leukocyte-associated regulation, and cell-junction organization. These annotations should be interpreted as transcriptional programs represented within the epithelial-fibroblast OTE rather than direct evidence of conventional immune-cell activity. Nonetheless, the shift shows that the limited hMPV-induced response moved away from a predominantly antiviral structure and toward a different set of regulatory and cellular-organization processes.

The large downregulated intersection between IAV and hMPV indicates that these viruses shared a substantial component of their transcriptional decreases, particularly at 72 h. However, this similarity did not make their overall responses equivalent. IAV retained a much larger upregulated antiviral program, whereas hMPV remained dominated by downregulation. Their common decreases therefore existed within two different response architectures: broad bidirectional activity for IAV and limited induction with extensive repression for hMPV.

RSVA showed the strongest temporal expansion of all four infections. Its early response was moderate and weighted toward downregulation, but by 72 h it had developed broad upregulated and downregulated components. This expansion was accompanied by a major change in functional organization. Transport- and localization-associated processes were already present at 24 h, but they became the dominant feature of the later response. Intracellular transport, protein transport, establishment of localization, cellular localization, nucleocytoplasmic transport, and organelle organization formed a consistent functional theme.

The exact virus-unique analysis reinforced the importance of this RSVA-associated pattern. RSVA developed the largest collection of unique upregulated biological processes at 72 h, and the leading terms repeatedly returned to intracellular trafficking, localization, and cellular organization. These terms were supported by overlapping groups of genes that included antiviral effectors, signaling components, stress-associated genes, trafficking factors, and regulators of cellular structure. The large number of related GO terms should be interpreted as one broad, interconnected biological theme rather than many independent mechanisms. The results indicate that the late RSVA response extended well beyond interferon activation and involved coordinated remodeling of intracellular organization and transport.

The RSVA downregulated response provided a complementary biological signal. Its leading processes were associated with cellular defense, immune-effector activity, cell killing, cytotoxic functions, chemokine signaling, and regulation of cytosolic calcium. Together, the upregulated and downregulated results indicate that RSVA combined expanding intracellular transport and localization activity with reduced expression of selected immune-effector and signaling programs.

RSVB remained the most restricted response across both time points. However, the limited response changed substantially in composition. The early profile was characterized by *IL6*, *CXCL10*, *PRKCA*, and other inflammatory or signaling-associated genes. By 72 h, the leading genes had shifted toward *IFI6*, *IFI27*, *MX1*, *OAS2*, *IFI44*, *ISG15*, and *IFIT1*. The major temporal feature of RSVB was therefore not a large increase in the number of responsive genes, but a transition from an early inflammatory and signaling-associated state to a later interferon-stimulated state.

The late RSVB response also became more similar to portions of the RSVA and IAV responses. Many RSVB-upregulated genes at 72 h belonged to groups shared with RSVA or with both RSVA and IAV, whereas relatively few genes remained specific to RSVB. Its GO profile was likewise centered on antiviral and interferon-associated functions, with very few exact unique processes at the later time point. RSVB therefore retained a recognizable antiviral response but developed less functional breadth and less virus-specific organization than RSVA.

The comparison between RSVA and RSVB is one of the main biological contributions of this study. The tested RSVA construct developed a broad late response involving both interferon-associated genes and extensive transport, localization, and organelle-organization processes. In contrast, the tested RSVB construct retained a smaller response and changed primarily through replacement of its leading early genes with later interferon-stimulated effectors. Thus, the two RSV groups differed not only in response magnitude but also in temporal behavior and functional organization. RSVA expanded, whereas RSVB reorganized within a comparatively narrow transcriptional space.

These RSV differences also help explain the predictive patterns. RSVA became increasingly distinguishable at 72 h as its transcriptional response expanded and acquired a broad functional identity. RSVB remained the class most closely positioned to Mock because its response involved fewer genes, fewer downregulated genes, and fewer unique biological processes. The remaining Mock-RSVB classification errors were therefore consistent with the underlying biology rather than representing an isolated modeling failure.

The exact unique GO analysis further separated common biology from infection-associated organization. IAV retained a broad defense- and cytokine-centered identity. hMPV developed a late profile associated with stress, catabolism, metabolism, differentiation, and cell-junction organization. RSVA became defined by intracellular transport and localization, while RSVB produced only a small number of unique processes. Among downregulated genes, hMPV was distinguished by extensive stress and degradative functions, RSVA by immune-effector and chemokine-associated functions, and IAV by cellular assembly, transport, and metabolic processes. These patterns demonstrate that each virus produced a distinct combination of activated and reduced biological programs.

The analytical framework captured these relationships at several complementary levels. RMAS prioritized genes that combined expression magnitude with statistical support, which concentrated infection- and time-associated structure that was less apparent when all measured genes were considered together. MGSR then retained the biological context of each response by separating genes into common, shared, and virus-associated groups. This step was important because a gene’s relevance depended not only on the strength of its response but also on whether that response was conserved across infections or restricted to one comparison.

HCNA added a network-based reduction step that prevented densely connected modules from dominating the final panels. Rather than selecting many genes from the same coordinated antiviral process, HCNA allowed different modules, smaller components, and singleton candidates to contribute representatives. Direction-aware RMAS anchors preserved strongly responsive genes that might not occupy central positions within the STRING network. The resulting panels therefore combined statistical evidence, cross-virus context, network representation, and reduced expression redundancy.

The classification results show that this structured reduction preserved biologically meaningful distinctions more effectively than the complete gene set. RMAS-HCNA achieved a mean outer-test Macro F1 of 0.859 and balanced accuracy of 0.888, compared with 0.604 and 0.616 for the all-gene comparator. Because both approaches used the same cross-validation folds, training-only harmonization, scaling, two-component PCA representation, logistic-regression classifier, and regularization-tuning procedure, the difference reflects the value of the selected feature space before dimensionality reduction.

Overall, the study identifies a shared interferon-associated airway response within four distinct temporal and functional trajectories. IAV maintained a broad antiviral program while developing extensive later transcriptional decreases. hMPV combined limited induction with widespread reductions in structural, stress-associated, trafficking, catabolic, and metabolic functions. RSVA showed the strongest temporal expansion and developed a broad late response centered on intracellular transport, localization, and organelle organization. RSVB remained the most restricted response but transitioned from early inflammatory and signaling-associated activity to a later interferon-stimulated state. RMAS-HCNA integrated these common and virus-associated signals into compact panels that preserved the major biological distinctions more effectively than the complete measured gene space.

### 5.1 Limitations and future directions

Several limitations define the scope of the conclusions. First, the NanoString nCounter Host Response Panel measured 773 targeted genes and therefore could not capture transcriptional programs outside this predefined host-response space. Future studies should apply genome-wide bulk RNA sequencing to independent airway OTE experiments and matched *in vivo* respiratory samples. These data could be analyzed using GLMQL-MAS (Rezapour et al., 2024d), which has supported gene selection in other viral RNA-seq applications, including Ebola (Rezapour et al., 2026b), Sars-Cov-2 (Rezapour et al., 2025b), Mpox (Rezapour et al., 2024e), Dengue and Chikungunya (Rezapour et al., 2026c), followed by MGSR and HCNA. This extension would determine whether the recurrent genes, cross-virus patterns, and biological processes identified here remain reproducible in a genome-wide setting.

Second, a sufficiently matched external dataset was not available for independent validation. An appropriate validation dataset would need to include IAV, hMPV, RSVA, RSVB, and source-matched controls under comparable multiplicities of infection, sampling times, tissue contexts, and measurement procedures. The nested cross-validation design provided a leakage-free internal assessment, but it cannot establish generalization to new donors, experimental batches, viral strains, laboratories, or measurement platforms. Future work should therefore generate an independent cohort in which all four viruses are evaluated concurrently under standardized experimental conditions.

Third, the study was limited to airway OTE measurements collected at 24 and 72 h post infection. These time points do not define the earliest detectable host response and do not capture intermediate transcriptional kinetics. In addition, NanoString measurements cannot distinguish the contributions of infected epithelial cells, uninfected bystander cells, fibroblasts, or changes in their relative transcriptional activity. Earlier and intermediate sampling, together with single-cell or spatially resolved profiling, would clarify the cellular origin and temporal development of the shared and virus-associated responses.

Finally, the study examined one recombinant EGFP-expressing construct from each RSV antigenic group. The observed differences between RSVA and RSVB may therefore reflect antigenic group, parental strain, codon optimization, replication kinetics, or other construct-specific characteristics. These findings should not be generalized to all RSV strains. Future studies should include multiple clinical strains from each antigenic group, quantitative measurements of infectious-virus production and replication kinetics, independent airway OTE experiments, and *in vivo* or clinical validation.

## 6 Conclusion

This study demonstrates that RSVA, RSVB, IAV, and hMPV activate a shared interferon-associated antiviral foundation while producing distinct transcriptional and functional trajectories in human airway OTEs. IAV maintained a broad antiviral response across both time points. hMPV combined limited gene induction with extensive downregulation of structural, stress-associated, catabolic, metabolic, and transport-related processes. RSVA showed the strongest temporal expansion and developed a broad late response centered on intracellular transport, cellular localization, and organelle organization. In contrast, RSVB remained the most restricted response and progressed from an early inflammatory and signaling-associated profile to a later interferon-stimulated state. These findings show that RSVA and RSVB differ not only in response magnitude but also in temporal development and biological organization.

The RMAS-HCNA framework integrated statistical evidence, cross-virus response patterns, network-module structure, and expression-redundancy control to identify compact and biologically representative gene panels. Within nested cross-validation, these panels distinguished Mock, IAV, hMPV, RSVA, and RSVB samples more effectively than the complete measured gene set and showed no evidence of substantial overfitting within the current datasets. Together, the biological and predictive findings support the value of structured gene selection for resolving closely related respiratory-virus responses. Independent validation using genome-wide expression data, additional viral strains, new OTE experiments, and *in vivo* samples remains necessary to establish the broader generalizability of these findings.

## Data Availability

Data generated in this study are available on Zenodo at https://doi.org/10.5281/zenodo.21970345.
