# Supplementary material for: Transcriptional signatures of respiratory syncytial virus (RSV) infection using relaxed magnitude altitude score with hub-centric network analysis (RMAS-HCNA)
Source: Front Genet. 2026 Sep 8;17:1879778. doi: 10.3389/fgene.2026.1879778 (PMC13593431; doi:10.3389/fgene.2026.1879778)
Supplement: Supplementary file 1 [file DataSheet1.pdf]

Supplementary Materials for

Transcriptional Signatures of Respiratory Syncytial Virus (RSV) Infection Using Relaxed Magnitude Altitude Score with Hub-Centric Network Analysis (RMAS-HCNA)

Mostafa Rezapour, PhD[1*]; David A. Ornelles, PhD[2]; Stephen J. Walker, PhD[1]; Colin E. Bishop, PhD[1]; Sean V. Murphy, PhD[1]; Metin Nafi Gurcan, PhD[3]; Patrick M. McNutt, PhD[1]; Anthony Atala, MD[1]

[1] Wake Forest Institute for Regenerative Medicine, Wake Forest University School of Medicine, Winston-Salem, NC, USA
[2] Department of Microbiology and Immunology, Wake Forest University School of Medicine, Winston-Salem, NC, USA
[3]Center for Artificial Intelligence Research, Wake Forest University School of Medicine, Winston-Salem, NC, USA

*** Correspondence:** Mostafa Rezapour; Mostafa.Rezapour@wfusm.edu

1. Algorithms

The following supplementary algorithms provide the complete procedural specification of the RMAS-HCNA framework. **Algorithm S1** describes source-matched differential-expression analysis and RMAS ranking. **Algorithm S2** integrates virus-specific RMAS results through MGSR. **Algorithm S3** describes network construction, module detection, hub ranking, and representative-gene selection through HCNA. **Algorithm S4** presents the complete nested cross-validation workflow used for hyperparameter selection, classification, outer-test evaluation, and gene-selection stability. Within the nested cross-validation, each algorithm was applied using training samples only.

1.1. Algorithm S1. Relaxed Magnitude Altitude Score (RMAS)

Algorithm S1 formalizes the gene-level differential-expression and RMAS-ranking procedure applied independently to each source-matched virus-time comparison. For each gene, the algorithm calculates the mean expression in the Mock and infected groups, determines the log_2 fold change using a pseudocount, and obtains a raw P-value using a two-sided Welch t-test with unequal variances. Genes meeting the prespecified significance criterion are classified as Upregulated or Downregulated according to the sign of the log_2 fold change.

**Algorithm S1. Relaxed Magnitude Altitude Score (RMAS)**
**Input:** Analysis-ready NanoString expression values for a Control group and a Treated group; common gene set G; pseudocount $\delta = 1$; significance threshold $\alpha = 0.05$. Here, $x_{C,g,i}$ and $x_{T,g,i}$ are the NanoString expression values of gene g in Control sample i and Treated sample i, respectively; n_C and n_T are the numbers of Control and Treated samples; $\mu_{C,g}$ and $\mu_{T,g}$ are the corresponding mean expression values; P_g is the raw Welch t-test P-value; Significant genes are retained using $P_g < 0.05$.<br><br>**Output:** Ranked table R containing the significant genes, mean expression in each group, log_2 fold change, P-value, expression direction, RMAS value, and RMAS rank.

1. Initialize the results table R.
2. **FOR** each gene $g \in G$ **DO**

 2.1. Calculate the mean expression of gene g in the Control group: $\mu_{C,g} = \frac{1}{n_C}\sum_{i=1}^{n_C} x_{C,g,i}$.

 2.2. Calculate the mean expression of gene g in the Treated group: $\mu_{T,g} = \frac{1}{n_T}\sum_{i=1}^{n_T} x_{T,g,i}$.

 2.3. Calculate the $\log_2$ fold change: $\log_2 FC_g = \log_2\left(\frac{\mu_{T,g}+\delta}{\mu_{C,g}+\delta}\right)$.

 2.4. Compare the Control and Treated expression values of gene g using a two-sided Welch t-test with unequal variances to obtain the raw P-value P_g.

 2.5. **IF** P_g is missing or nonfinite **THEN**

  Set $P_g = 1$.

 END IF

 2.6. Calculate the Relaxed Magnitude Altitude Score: $RMAS_g = \mid \log_2 FC_g \mid [-\log_{10}(P_g)]$

 2.7. **IF** $\log_2 FC_g \geq 0$ **THEN**

  Set $Direction_g$ = Upregulated.

 ELSE

  Set $Direction_g$ = Downregulated.

 END IF

 2.8. Store g, $\mu_{C,g}$, $\mu_{T,g}$, $\log_2 FC_g$, P_g, $Direction_g$, and $RMAS_g$ in R.

END FOR

3. Retain genes satisfying:

$$P_g < \alpha.$$

4. Rank the retained genes by decreasing $RMAS_g$.
5. Assign $RMAS\ Rank_g = 1$ to the highest-ranked gene and assign sequential ranks to the remaining genes.

Return the ranked significant-gene table R.

1.2. Algorithm S2. Multiple Gene-Set Ranking (MGSR)

Algorithm S2 formalizes the integration of the virus-specific RMAS results produced by **Algorithm S1**. RMAS ranks genes independently within each source-matched virus-time comparison, but it does not determine whether a transcriptional response is conserved across all four viruses, shared by a subset of viruses, or associated with only one infection. MGSR addresses this limitation by comparing the IAV, hMPV, RSVA, and RSVB results separately at 24 and 72 hours and organizing significant genes according to their cross-virus support, expression direction, and relative response strength.

Because RMAS values and absolute log_2 fold changes may have different numerical ranges across virus-time comparisons, both quantities were transformed to percentile ranks before candidate-group assignment. For a numerical quantity $Z_{v,t}(g)$, let $G_{v,t}$ denote the set of all genes measured for virus v at time t, and let

$$n_{v,t} = \mid G_{v,t} \mid.$$

The ascending average rank of gene g within the corresponding virus-time comparison was defined as

$$AR_{v,t}(Z(g)) = 1 + \sum_{\substack{h \in G_{v,t} \\ h \neq g}} \mathbf{1}\left[Z_{v,t}(h) < Z_{v,t}(g)\right] + \frac{1}{2}\sum_{\substack{h \in G_{v,t} \\ h \neq g}} \mathbf{1}\left[Z_{v,t}(h) = Z_{v,t}(g)\right].$$

The corresponding percentile rank was defined as

$$PR_{v,t}(Z(g)) = \frac{AR_{v,t}(Z(g))}{n_{v,t}}.$$

Here, $\mathbf{1}[\cdot]$ equals 1 when the stated condition is true and 0 otherwise. Tied values receive the average of their rank positions, and larger values receive larger percentile ranks. This transformation was applied separately to RMAS and absolute log_2 fold change within each virus-time comparison. Accordingly,

$$r_{v,t}(g) = PR_{v,t}\big(RMAS_{v,t}(g)\big)$$

denotes the RMAS percentile rank, while

$$f_{v,t}(g) = PR_{v,t}\big(|\log_2 FC_{v,t}(g)|\big)$$

denotes the absolute-log_2-fold-change percentile rank.

For every gene, MGSR recorded the number and identity of viruses in which the gene met the significance criterion of $P < 0.05$, together with its expression direction in the supporting comparisons. Genes significant in all four viruses with a consistent direction were assigned to the PanViral group. Genes significant in at least two viruses but not meeting the PanViral definition were assigned to the Shared group. Shared genes were classified as Upregulated or Downregulated when their directions were consistent across the supporting viruses and as Mixed when both directions were present. Genes significant in only one virus were assigned to the corresponding IAV-specific, hMPV-specific, RSVA-specific, or RSVB-specific group.

The group-specific scoring functions incorporated both the breadth and strength of the response. The PanViral score combined the minimum and average RMAS percentile ranks across all four viruses with the average absolute-log_2-fold-change percentile rank. Including the minimum RMAS percentile favored genes with consistently strong evidence and prevented an exceptionally strong response in one virus from compensating for weaker responses in the others.

The Shared score incorporated the proportion of supporting viruses and calculated the RMAS and absolute-fold-change components only across those viruses. Virus-specific scores combined the RMAS and absolute-log_2-fold-change percentile ranks from the single supporting virus. Genes were ranked by decreasing group-specific score within each biological group and time point.

MGSR therefore preserved both the breadth and relative strength of the transcriptional response. Its outputs were ranked PanViral, Shared, IAV-specific, hMPV-specific, RSVA-specific, and RSVB-specific candidate-gene tables at 24 and 72 hours. These ranked groups provided the inputs to HCNA in **Algorithm S3**. The complete significance indicators, group-assignment rules, scoring functions, direction classifications, and candidate-ranking procedure are provided below in **Algorithm S2**.

**Algorithm S2. Multiple Gene-Set Ranking (MGSR)**
**Input:** RMAS results from **Algorithm S1** for IAV versus Mock-IM, hMPV versus Mock-IM, RSVA versus Mock-R, and RSVB versus Mock-R at 24 and 72 h; virus set $V = \{IAV, hMPV, RSVA, RSVB\}$; significance threshold $\alpha = 0.05$. Here, $P_{v,t}(g)$ is the raw P-value of gene g for virus v at time t; $r_{v,t}(g)$ is its RMAS percentile rank; $f_{v,t}(g)$ is its absolute-log_2-fold-change percentile rank; and $m_t(g)$ is the number of viruses for which the gene is significant. Percentile ranks are calculated across all measured genes within each virus-time comparison before the significant genes are assigned to candidate groups.<br><br>**Output:** Ranked PanViral, Shared, IAV-specific, hMPV-specific, RSVA-specific, and RSVB-specific gene tables for each time point.<br><br>**1.** Initialize the candidate-gene tables.

2. **FOR** each time point $t \in \{24,72\}$ **DO**

2.1. Obtain the four time-specific RMAS tables produced by **Algorithm S1**.

2.2. **FOR** each virus $v \in V$ **DO**

FOR each measured gene $g \in G_{v,t}$ **DO**

- Define the significance indicator:

$$I_{v,t}(g) = \begin{cases} 1, & P_{v,t}(g) < \alpha \\ 0, & P_{v,t}(g) \geq \alpha \end{cases}$$

- Convert the RMAS value into a percentile rank:

$$r_{v,t}(g) = PR_{v,t}\big(RMAS_{v,t}(g)\big)$$

- Convert the absolute $\log_2$fold change into a percentile rank:

$$f_{v,t}(g) = PR_{v,t}\big(|\log_2 FC_{v,t}(g)|\big)$$

END FOR

END FOR

2.3. Merge the four virus-specific tables by gene symbol.

2.4. **FOR** each gene g **DO**

2.4.1. Calculate the number of viruses for which gene g is significant:

$$m_t(g) = \sum_{v \in V} I_{v,t}(g).$$

2.4.2. Define the set of viruses supporting gene g:

$$V_t(g) = \{v \in V : I_{v,t}(g) = 1\}.$$

2.4.3. Record the four-virus UpSet membership pattern:

$$U_t(g) = \big(I_{IAV,t}(g), I_{hMPV,t}(g), I_{RSVA,t}(g), I_{RSVB,t}(g)\big).$$

2.4.4. Determine whether the expression direction of gene g is identical across all viruses in $V_t(g)$.

2.4.5.

IF $m_t(g) = 4$ and the direction is identical across all four viruses **THEN**

- Assign gene g to the PanViral group.
- Calculate the PanViral score:

$$S_{Pan,t}(g) = \min_{v \in V} r_{v,t}(g) + \frac{1}{4}\sum_{v \in V} r_{v,t}(g) + \frac{1}{4}\sum_{v \in V} f_{v,t}(g).$$

- Assign the common Upregulated or Downregulated direction.

ELSE IF $m_t(g) \geq 2$ **THEN**

- Assign gene g to the Shared non-pan-viral group.
- Calculate the Shared score using only the supporting viruses:

$$S_{Shared,t}(g) = \frac{m_t(g)}{4} + \min_{v \in V_t(g)} r_{v,t}(g) + \frac{1}{m_t(g)}\sum_{v \in V_t(g)} r_{v,t}(g) + \frac{1}{m_t(g)}\sum_{v \in V_t(g)} f_{v,t}(g).$$

IF the direction is identical across all supporting viruses **THEN**

Assign the common Upregulated or Downregulated direction.

ELSE

Assign the direction as Mixed.

END IF

ELSE IF $m_t(g) = 1$ **THEN**

- Let v be the only virus for which gene g is significant.
- Assign gene g to the corresponding IAV-specific, hMPV-specific, RSVA-specific, or RSVB-specific group.
- Calculate the virus-specific score: $S_{v,t}(g) = r_{v,t}(g) + f_{v,t}(g)$.
- Assign the Upregulated or Downregulated direction observed for virus v.

ELSE

Exclude gene g from all candidate groups.
END IF
END FOR
2.5. **FOR** each group $b \in \{PanViral, Shared, IAV, hMPV, RSVA, RSVB\}$ **DO**
- Rank genes by decreasing group-specific score.
- Assign Candidate Rank 1 to the highest-ranked gene and sequential ranks to the remaining genes.

END FOR
END FOR
3. Return the six ranked candidate-gene tables for 24 h and the six ranked candidate-gene tables for 72h.

1.3. Algorithm S3. Hub-Centric Network Analysis (HCNA)

Algorithm S3 formalizes the network-based reduction of the MGSR-ranked candidate genes. RMAS ranks genes within each source-matched virus comparison, while MGSR integrates those rankings across viruses and organizes genes according to cross-virus support, expression direction, and relative response strength. HCNA adds a functional-network layer to this process by identifying network modules and selecting representative genes from each module. This approach preserves broad biological coverage while limiting redundant selection of functionally related or highly correlated genes.

For each MGSR biological group and time point, the top K ranked genes were entered into the human STRING v12.0 functional association network using a minimum interaction score of 600. When both Upregulated and Downregulated genes were available but the top K candidates represented only one direction, the lowest-ranked retained gene was replaced with the highest-ranked candidate from the missing direction.

Networks were constructed and analyzed using NetworkX v3.4.2. Connected components containing more than three genes were divided using weighted Leiden clustering, implemented with python-igraph v1.0.0 and leidenalg v0.11.0 at a resolution of 1.0. Components containing two or three genes were retained as individual modules. Genes without qualifying STRING associations were preserved as singleton modules. This prevented strongly supported MGSR candidates from being excluded solely because they lacked an association above the selected STRING threshold.

Let $M = (V_M, E_M)$ denote a detected module, where V_M and E_M are its gene and edge sets, respectively. Let $g \in V_M$ denote a gene, $n_M = | V_M |$ denote the number of genes in the module, $N_M(g)$ denote the neighbors of g, and w_{gu} denote the STRING association score between genes g and u. Five complementary network measures were calculated for each gene: weighted degree, PageRank, weighted betweenness centrality, core number, and bootstrap maximum-core stability.

The weighted degree of gene g was defined as the sum of the STRING association scores of its incident edges:

$$d_M^w(g) = \sum_{u \in N_M(g)} w_{gu}.$$

Weighted PageRank measured the importance of a gene according to both the strengths of its associations and the PageRank values of its neighboring genes:

$$PR_M(g) = \frac{1-\alpha}{n_M} + \alpha \sum_{u \in N_M(g)} PR_M(u) \frac{w_{ug}}{\sum_{v \in N_M(u)} w_{uv}},$$

where $\alpha = 0.85$ was the PageRank damping factor, which balances propagation through weighted network connections with a small restart component. For a singleton module, $PR_M(g)$ was set to 1. For weighted betweenness centrality, STRING association strengths were converted to path distances:

$$\ell_{uv} = \frac{1}{\max(w_{uv}, \varepsilon)},$$

where ε prevented division by zero. Weighted betweenness centrality was then defined as

$$BC_M(g) = \frac{2}{(n_M - 1)(n_M - 2)} \sum_{\substack{s<t \\ s,t \in V_M \setminus \{g\}}} \frac{\sigma_{st}(g)}{\sigma_{st}},$$

where σ_{st} is the number of shortest paths between genes $s$and t, and $\sigma_{st}(g)$ is the number of those paths that pass through gene g. For modules containing fewer than three genes, $BC_M(g)$ was set to 0. The core number of gene g was defined as $k_M(g) = \max\{k: g \in C_k(M)\}$, where $C_k(M)$ is the maximal subgraph of module M in which every gene has an unweighted degree of at least k. For a singleton module, $k_M(g)$ was set to 0.

Bootstrap maximum-core stability was estimated by resampling the module edges with replacement B times. For bootstrap iteration b, a simple bootstrap network $M^{(b)} = \left(V_M, E_M^{(b)}\right)$ was constructed using all genes from the original module. The bootstrap edge set $E_M^{(b)}$ was generated by sampling $| E_M |$ edges with replacement from E_M. Repeated draws of the same edge did not create parallel edges in the resulting simple graph.

Let $V_{M,+}^{(b)} = \left\{g \in V_M : \deg_{M^{(b)}}(g) > 0\right\}$ denote the nonisolated genes in bootstrap network b. Unweighted core numbers $k_M^{(b)}(g)$ were calculated for these genes, and the maximum bootstrap core number was

$$k_{\max} = \max_{u \in V_{M,+}^{(b)}} k_M^{(b)}(u).$$

The bootstrap maximum-core stability of gene g was calculated as

$$s_M(g) = \frac{1}{B} \sum_{b=1}^{B} \mathbf{1}\left[g \in V_{M,+}^{(b)} \text{ and } k_M^{(b)}(g) = k_{\max}\right].$$

Here, $\mathbf{1}[\cdot]$ equals 1 when the stated condition is true and 0 otherwise. Thus, $s_M(g)$ represents the proportion of bootstrap networks in which gene g belonged to the maximum core. For a singleton module, $s_M(g)$ was set to 1.

The five measures capture complementary aspects of within-module importance: total association strength, influence through neighboring genes, participation in shortest communication paths, membership in densely connected cores, and stability of core membership under network resampling. Because modules may differ in size and density, each network measure was converted to an ascending percentile rank within its corresponding module. For any network measure $Z_M(g)$, the ascending average rank was defined as

$$AR_M(Z_M(g)) = 1 + \sum_{\substack{h \in V_M \\ h \neq g}} \mathbf{1}\left[Z_M(h) < Z_M(g)\right] + \frac{1}{2} \sum_{\substack{h \in V_M \\ h \neq g}} \mathbf{1}\left[Z_M(h) = Z_M(g)\right].$$

The corresponding within-module percentile rank was

$$Q_M(Z_M(g)) = \frac{AR_M(Z_M(g))}{n_M}.$$

Tied values received the average of their rank positions, and larger network-measure values received larger percentile ranks. For a singleton module, the only gene received a percentile rank of 1 for every measure. The five within-module percentile ranks were averaged to calculate the hub score:

$$H_M(g) = \frac{Q_M(d_M^w(g)) + Q_M(PR_M(g)) + Q_M(BC_M(g)) + Q_M(k_M(g)) + Q_M(s_M(g))}{5}.$$

Genes were ranked within each module by decreasing hub score, followed by decreasing MGSR score, decreasing bootstrap stability, and gene symbol. The highest hub-ranked gene was retained as the first module representative. When more than one representative was permitted, additional genes were selected according to hub rank and expression redundancy. Let S_M denote the set of representatives already selected from module M. For each remaining gene g, the maximum absolute Spearman correlation with the selected representatives was calculated using time-matched training expression values:

$$r_M(g; S_M) = \max_{h \in S_M} |\, \rho_{\mathrm{Sp}}(X_g, X_h) \,|.$$

Genes satisfying

$$r_M(g; S_M) < \tau,$$

where $\tau = 0.85$, were preferred according to their hub ranks. This stringent threshold treated only genes with highly similar monotonic expression profiles as redundant, while preserving moderately correlated genes that could represent distinct biological information. When every remaining gene had a maximum absolute correlation of at least 0.85, the gene with the lowest maximum correlation was selected, followed by hub rank. Up to $q$representatives were retained from each module.

Representatives selected across all modules, MGSR groups, and time points were combined and deduplicated. When the same gene was selected from multiple sources, the occurrence with the highest hub score was retained, followed by the highest MGSR score and bootstrap stability. All contributing modules, biological groups, expression directions, and time points were recorded.

Algorithm S3 provides the complete sequence for selecting the top K MGSR candidates, constructing the STRING networks, identifying modules, calculating network measures and hub scores, retaining up to q nonredundant representatives per module, and producing the final deduplicated HCNA panel.

**Algorithm S3. Hub-Centric Network Analysis (HCNA)**
**Input:** MGSR-ranked gene tables for each biological group and time point; number of genes entering STRING, K; number of representatives per module q; time-matched training expression values; STRING interaction-score threshold 600; absolute Spearman correlation threshold $\tau = 0.85$; Leiden resolution 1.0; and number of bootstrap iterations $B = 1000$.<br><br>**Output:** Detected network modules, hub-ranked genes within each module, and a deduplicated HCNA panel containing up to q representative genes per module.<br>**1.** Initialize the network, module, and selected-gene tables.<br>**2.** **FOR** each time point and MGSR biological group **DO**<br>2.1. Retain the top $\min(K, n)$ MGSR-ranked genes, where n is the number of available genes.<br>2.2. Construct the STRING-induced network from the retained genes.<br>• Preserve genes without STRING interactions as isolated nodes.<br>• Treat an isolated gene as a singleton module.<br>• Treat a connected component containing two or three genes as one module.<br>• Apply weighted Leiden clustering to each connected component containing more than three genes.<br>2.3. **FOR** each detected module M **DO**

- For each gene $g \in M$,
  - Calculate $d_M^w(g), PR_M(g), BC_M(g), k_M(g)$, and $s_M(g)$.
  - Calculate the hub score:

$$H_M(g) = \frac{Q_M(d_M^w(g)) + Q_M(PR_M(g)) + Q_M(BC_M(g)) + Q_M(k_M(g)) + Q_M(s_M(g))}{5}$$

where, $Q_M(\cdot)$ denotes the ascending percentile rank of a network measure among all genes in module M. Therefore, $H_M(g)$ is the mean percentile rank of the five network measures, where a larger value indicates stronger hub characteristics. Rank genes by decreasing hub score, followed by decreasing MGSR score, and decreasing network stability.

- Select the highest-ranked gene as the first module representative.
- **WHILE** fewer than $\min(q, \mid M \mid)$ genes have been selected **DO**
  - For each remaining gene, calculate its maximum absolute Spearman correlation with the previously selected genes using the time-matched training samples.
  - **IF** one or more genes have a maximum absolute correlation below τ **THEN**
    - Select the eligible gene with the highest hub rank, followed by the lowest maximum correlation.

    ELSE
    - Select the gene with the lowest maximum correlation, followed by the highest hub rank.

    END IF

  END WHILE

END FOR

END FOR

3. Combine the representatives selected from all modules, biological groups, and time points.
4. **FOR** each gene selected from more than one source **DO**
   - Retain the occurrence with the highest hub score, followed by the highest MGSR score and highest network stability.
   - Record all contributing modules, biological groups, time points, and expression directions.

   END FOR
5. Rank the deduplicated genes by decreasing hub score, followed by decreasing MGSR score.
6. Assign HCNA Rank 1 to the highest-ranked gene and sequential ranks to the remaining genes.
7. Return the module assignments, within-module hub rankings, selected module representatives, and final HCNA gene panel.

1.4. Algorithm S4. Nested Cross-Validation for RMAS-HCNA Gene Selection and Classification

Algorithm S4 describes the complete leakage-free evaluation of the RMAS-HCNA framework. Five outer folds were used to estimate generalization performance, while four inner folds within each outer-training partition were used to select the number of MGSR-ranked genes entering STRING, K; the maximum number of representatives retained per module, q; the number of direction-aware RMAS anchors, A; and the logistic-regression regularization parameter, C.

**Algorithm S4. Nested Cross-Validation for RMAS-HCNA Gene Selection and Classification**
**Input:** Dataset A containing IAV, hMPV, and Mock-IM NanoString samples at 24 and 72 h; Dataset B containing RSVA, RSVB, and Mock-R NanoString samples at 24 and 72 h; shared gene set; five outer folds; four inner folds; $K \in \{5,10,15,20,25,30,35,40\}$; $q \in \{1,2,\ldots,10\}$; $A \in \{0,2,5\}$; $C \in$

{0.01,0.1,1,10}; two principal components; and the STRING, Leiden, bootstrap, and correlation settings defined in HCNA.

Output: Outer-test class predictions and probabilities; fold-specific selected genes and hyperparameters; pooled classification metrics and confusion matrices; and gene-selection frequencies.

1. Align Dataset A and Dataset B by the shared genes while preserving their source identities. Assign Mock-IM and Mock-R to the classification class Mock and define the cross-validation strata by source group and time point.
2. Generate five stratified outer folds.
3. **FOR** each outer fold o **DO**

   3.1. Set one outer fold aside as $Test_o$, use the remaining four folds as $Train_o$, and generate four stratified inner folds from $Train_o$.

   3.2. **FOR** each inner fold i **DO**

   - Set one inner fold aside as $Valid_{o,i}$ and use the remaining three folds as $Train_{o,i}$.
   - **Apply RMAS:** Using only $Train_{o,i}$, apply RMAS independently to IAV versus Mock-IM, hMPV versus Mock-IM, RSVA versus Mock-R, and RSVB versus Mock-R at 24 and 72 h. This produces eight training-specific RMAS tables.
   - **Apply MGSR:** Apply MGSR to the four RMAS tables at each time point. This produces ranked PanViral, Shared, IAV-specific, hMPV-specific, RSVA-specific, and RSVB-specific gene tables at 24 and 72 h.
   - **FOR** each $K \in \{5,10,15,20,25,30,35,40\}$ **DO**
     - Retain the top K MGSR-ranked genes from each biological group and time point.
     - **Begin HCNA:** Construct the STRING networks, identify network modules, calculate the network measures and bootstrap stability, calculate hub scores, and rank genes within each module.

     END FOR
   - Fit separate 24-h and 72-h Mock-anchored ComBat models using only $Train_{o,i}$. Apply the fitted parameters to $Train_{o,i}$ and $Valid_{o,i}$.
   - **FOR** each combination of K and $q \in \{1,2,\ldots,10\}$ **DO**
     - **Complete HCNA:** Select up to q representatives from each module. Use the ComBat-corrected, time-matched training expression only for the Spearman-correlation redundancy filter.
     - Combine and deduplicate the selected module representatives to form the HCNA panel.
     - **FOR** each $A \in \{0, 2, 5\}$ **DO**
       - Return to the original RMAS tables and select up to A direction-aware RMAS anchor genes from each virus-time comparison.
       - Combine and deduplicate the RMAS anchor genes.
       - Form the final gene panel by combining the HCNA panel and the RMAS anchor panel.
       - **IF** the final panel contains at least two genes **THEN**
         - Fit a *StandardScaler* using $Train_{o,i}$ only.
         - Fit principal component analysis using $Train_{o,i}$ only and retain PC1 and PC2.
         - Transform $Train_{o,i}$ and $Valid_{o,i}$ using the fitted scaler and principal component analysis model.
         - **FOR** each $C \in \{0.01, 0.1, 1, 10\}$ **DO**
           - Fit balanced L2-regularized multiclass logistic regression using PC1 and PC2 from $Train_{o,i}$.

- Predict Mock, IAV, hMPV, RSVA, and RSVB classes and probabilities for $Valid_{o,i}$.
- Record macro F1, balanced accuracy, accuracy, classification errors, and final panel size.

END FOR

END IF

END FOR

END FOR

- Fit the all-gene comparator using the same ComBat, scaling, PC1-PC2, logistic regression, and C-tuning process without RMAS, MGSR, HCNA, or RMAS-anchor selection.

END FOR

3.3. Aggregate the results for each (K, q, A, C) configuration across the four inner folds.

3.4. Retain configurations within one standard error of the highest mean macro F1.

3.5. Select the final configuration by prioritizing lower infected-to-Mock error, higher Mock-separation balanced accuracy, lower Mock-to-infected error, smaller final panel size, and smaller hyperparameter values.

3.6. Using all samples in $Train_o$, repeat the complete training process in the same order.

3.7. Predict the classes and class probabilities of the held-out $Test_o$samples.

3.8. Fit and evaluate the all-gene comparator on the same outer fold.

3.9. Save the selected genes, selected hyperparameters, fitted models, predictions, and probabilities.

END FOR

4. Pool the held-out predictions from the five outer folds so that each sample has exactly one prediction from a model that did not use that sample during RMAS, MGSR, HCNA, ComBat estimation, hyperparameter tuning, PCA, or classifier training.

5. Calculate pooled macro F1, balanced accuracy, accuracy, classification errors, and confusion matrices for the selected-gene and all-gene models.

6. Calculate each gene's selection frequency as the proportion of outer folds in which it was included in the final panel.

7. Return the pooled predictions, performance metrics, fold-specific selected panels, selected hyperparameters, and gene-selection frequencies.

2. Figures

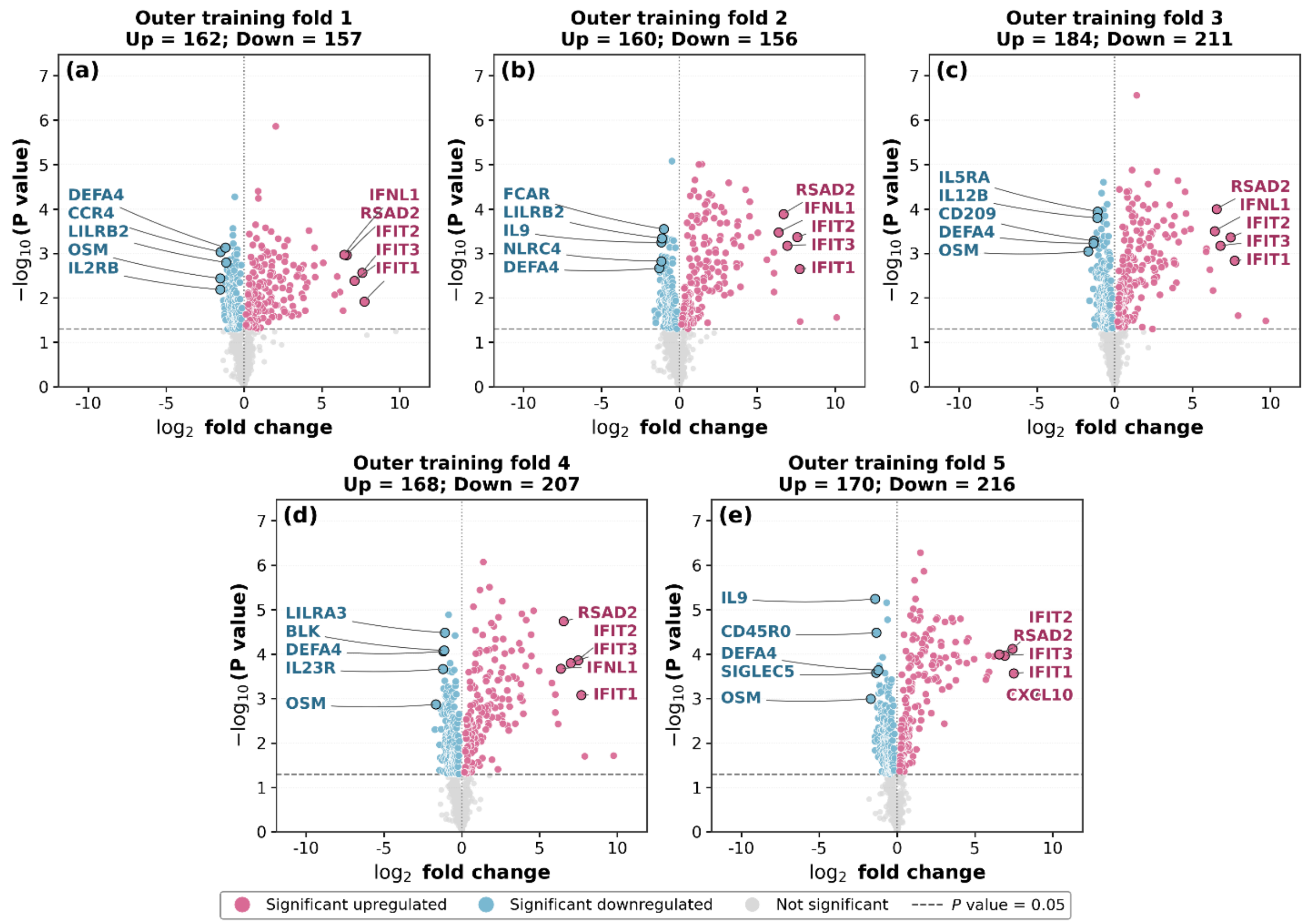

Figure S1. IAV versus Mock-IM at 24 h. *Volcano plots show IAV relative to source-matched Mock-IM within outer-training folds 1 through 5. The numbers of significant upregulated and downregulated genes are shown above each panel, and the five highest-ranked genes in each direction are labeled.*

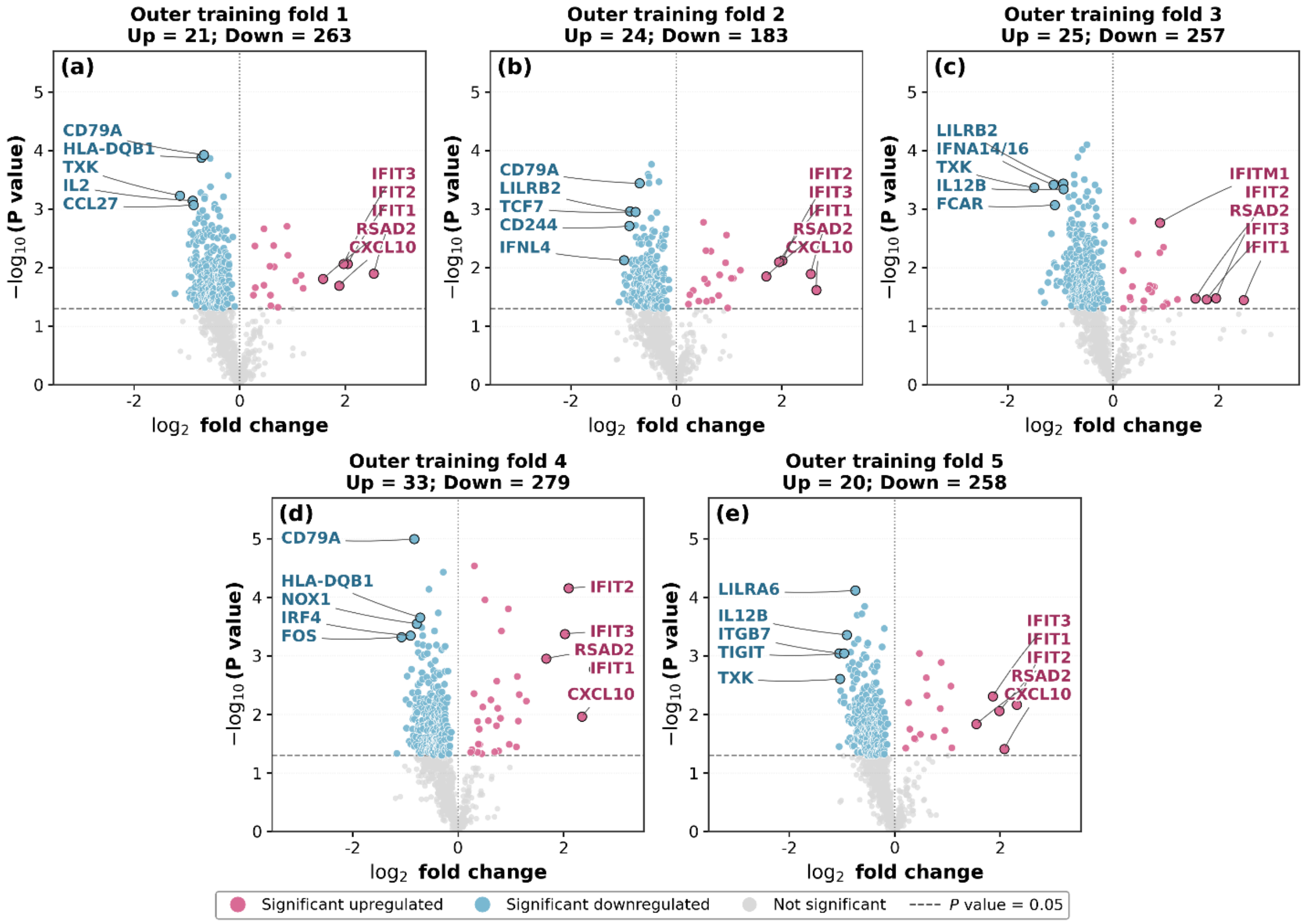

Figure S2. hMPV versus Mock-IM at 24 h. *Volcano plots show hMPV relative to source-matched Mock-IM within outer-training folds 1 through 5.*

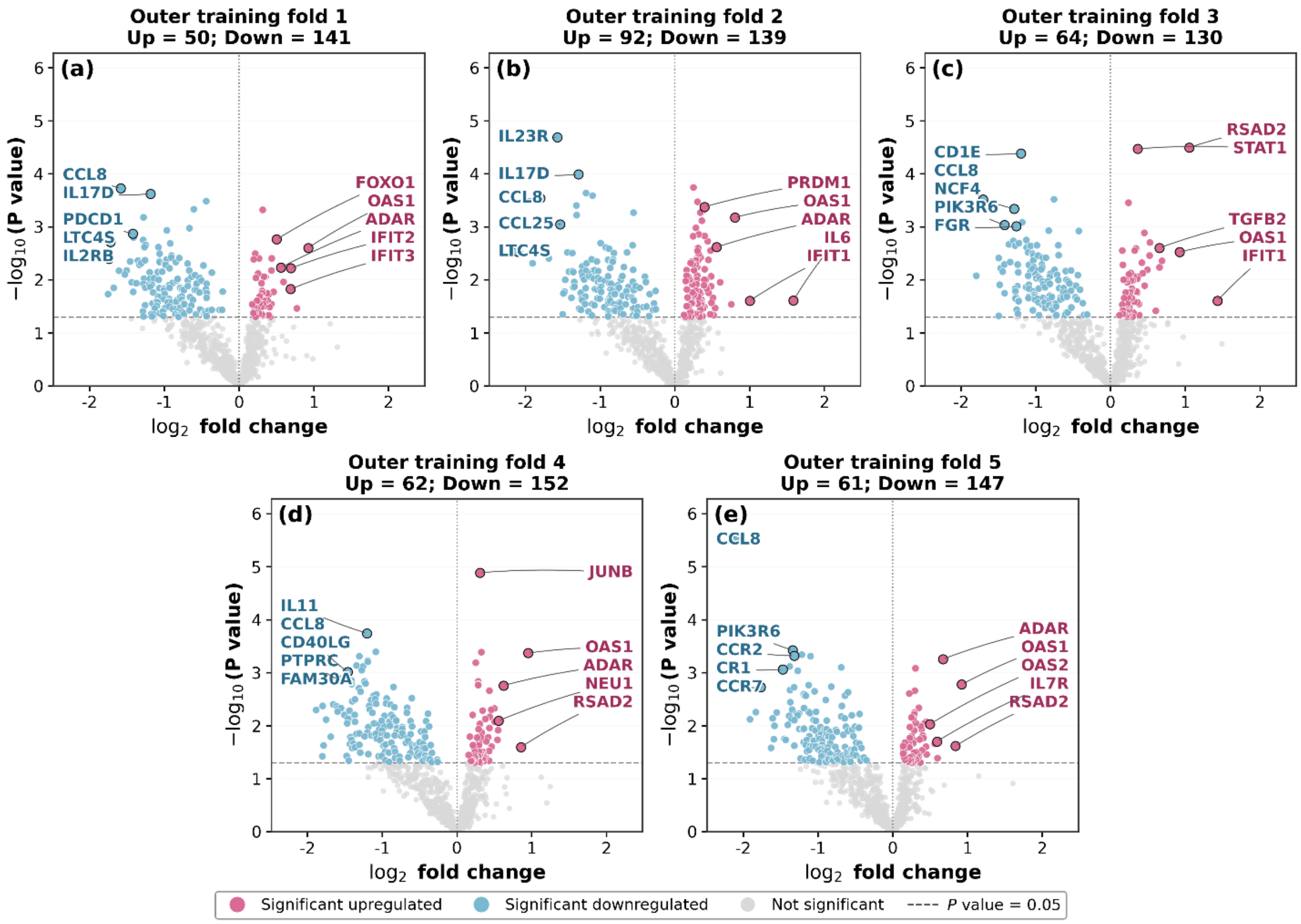

Figure S3. RSVA versus Mock-R at 24 h. *Volcano plots show RSVA relative to source-matched Mock-R within outer-training folds 1 through 5.*

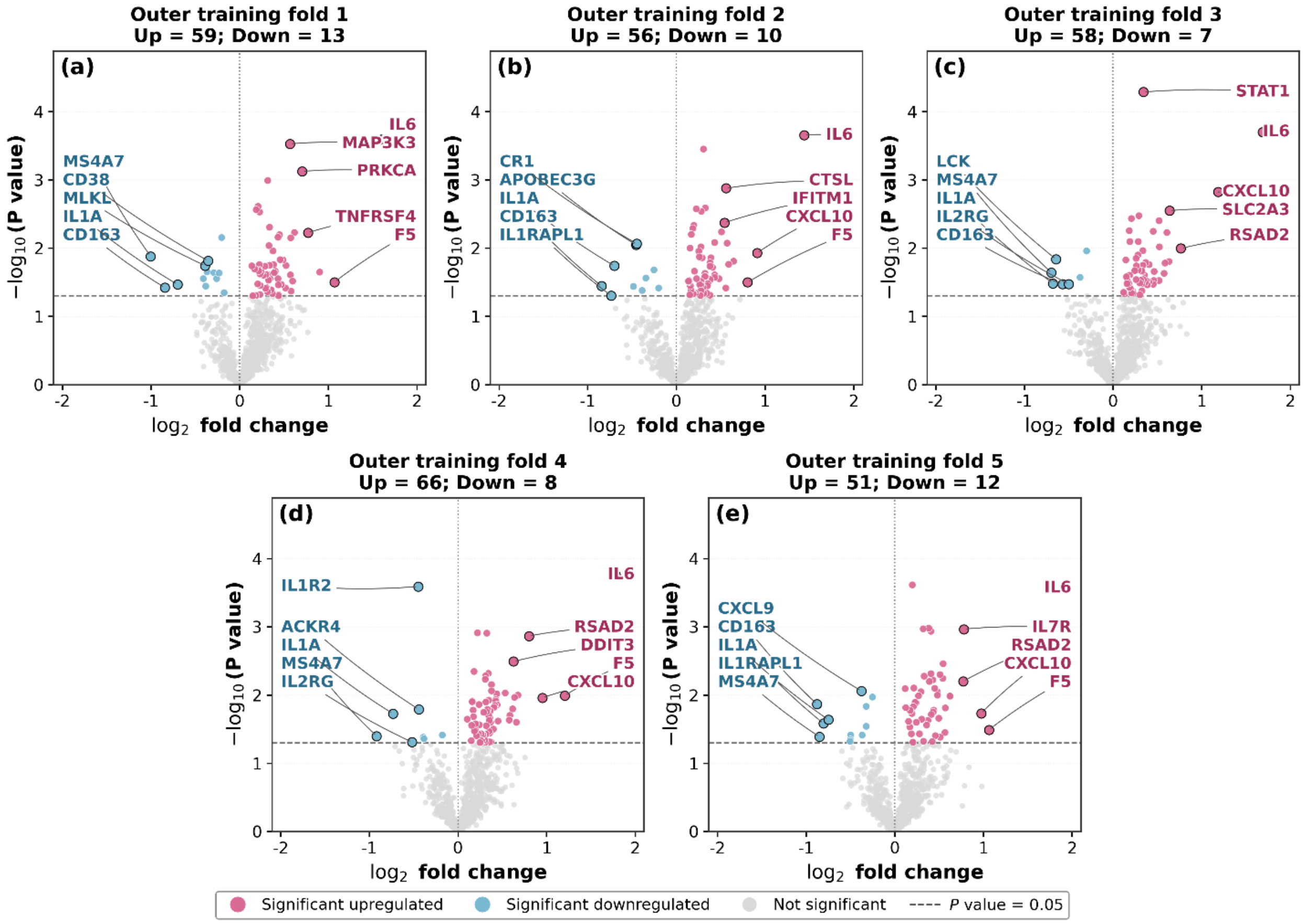

Figure S4. RSVB versus Mock-R at 24 h. *Volcano plots show RSVB relative to source-matched Mock-R within outer-training folds 1 through 5.*

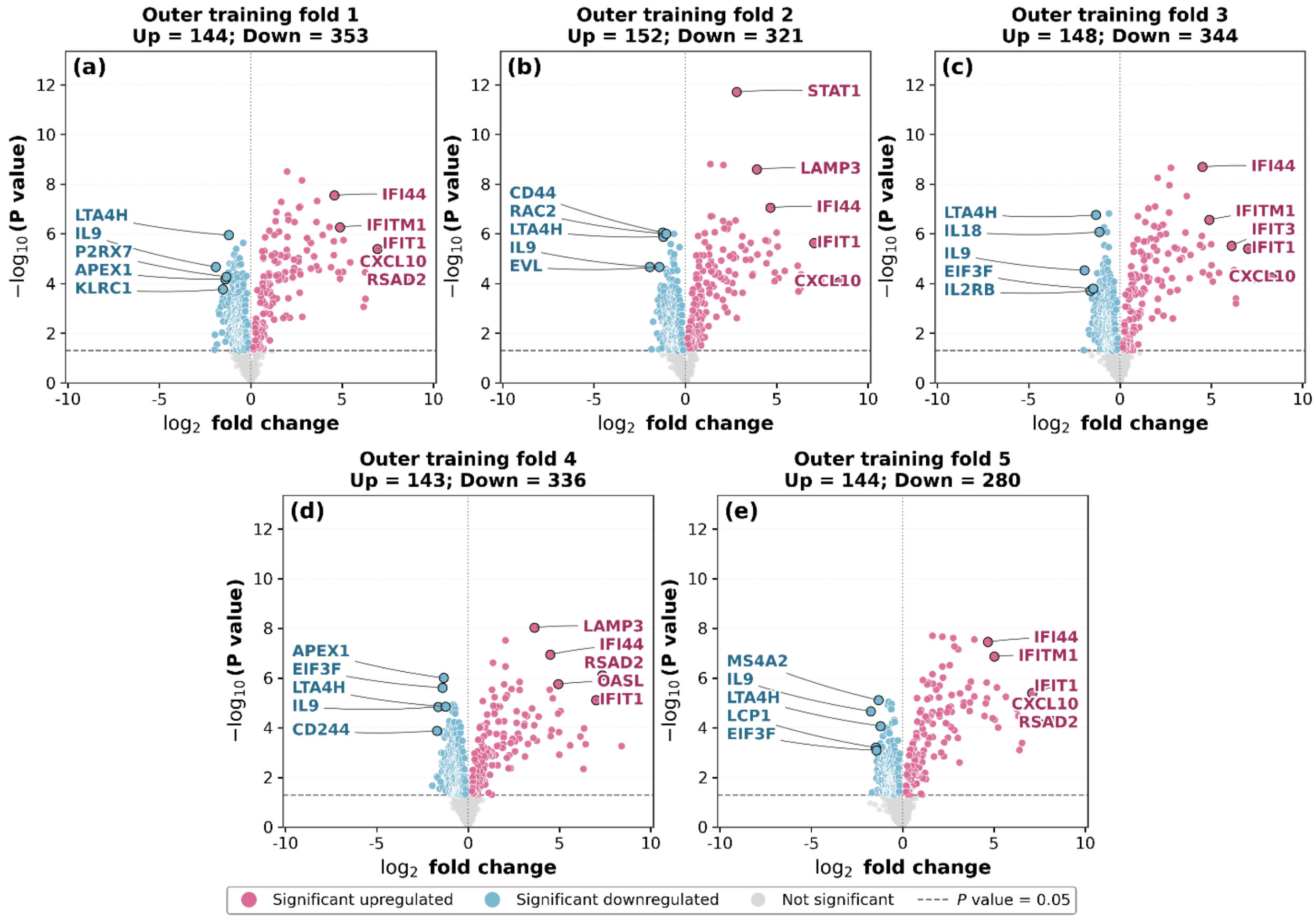

Figure S5. IAV versus Mock-IM at 72 h. *Volcano plots show IAV relative to source-matched Mock-IM within outer-training folds 1 through 5.*

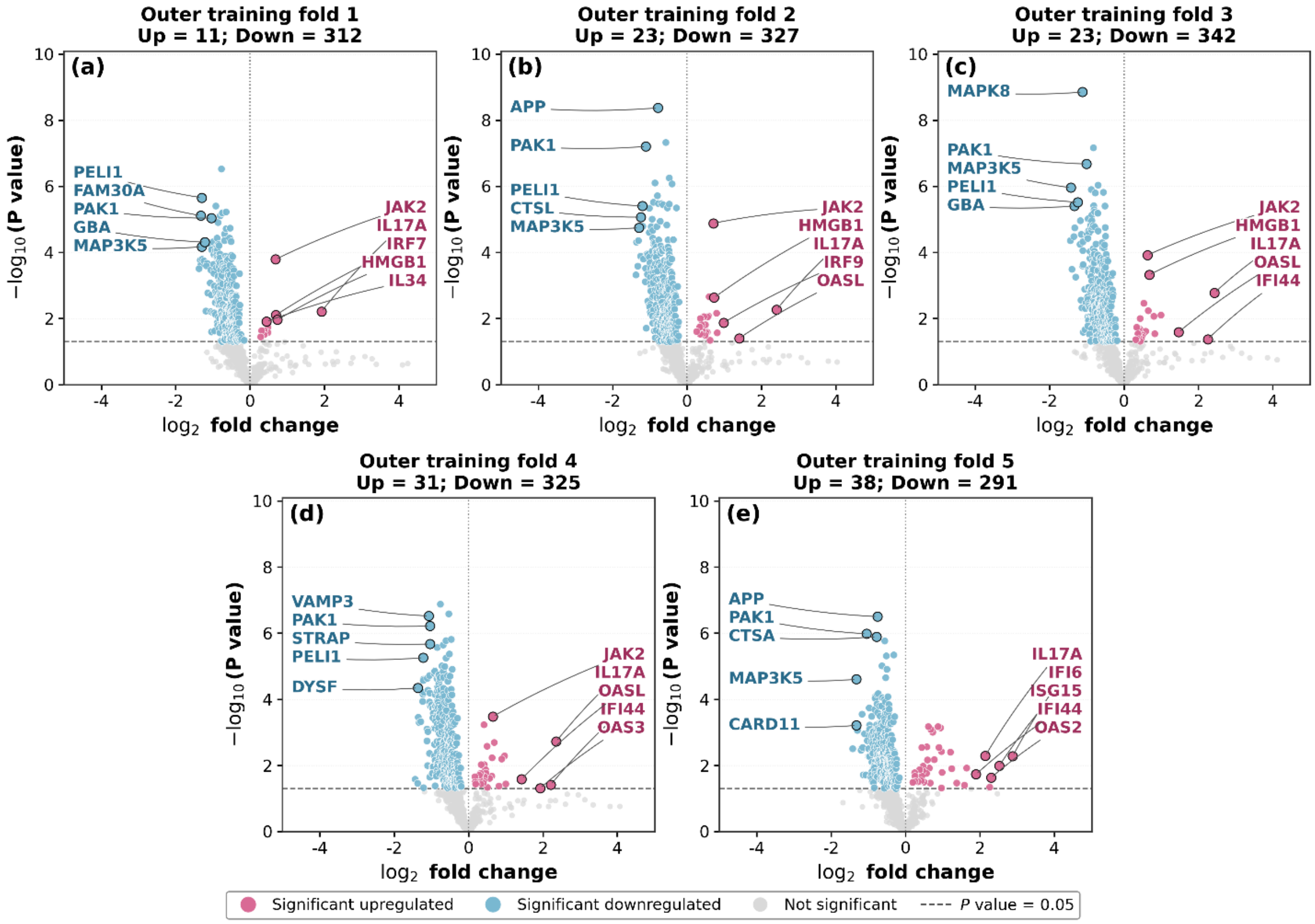

Figure S6. hMPV versus Mock-IM at 72 h. *Volcano plots show hMPV relative to source-matched Mock-IM within outer-training folds 1 through 5.*

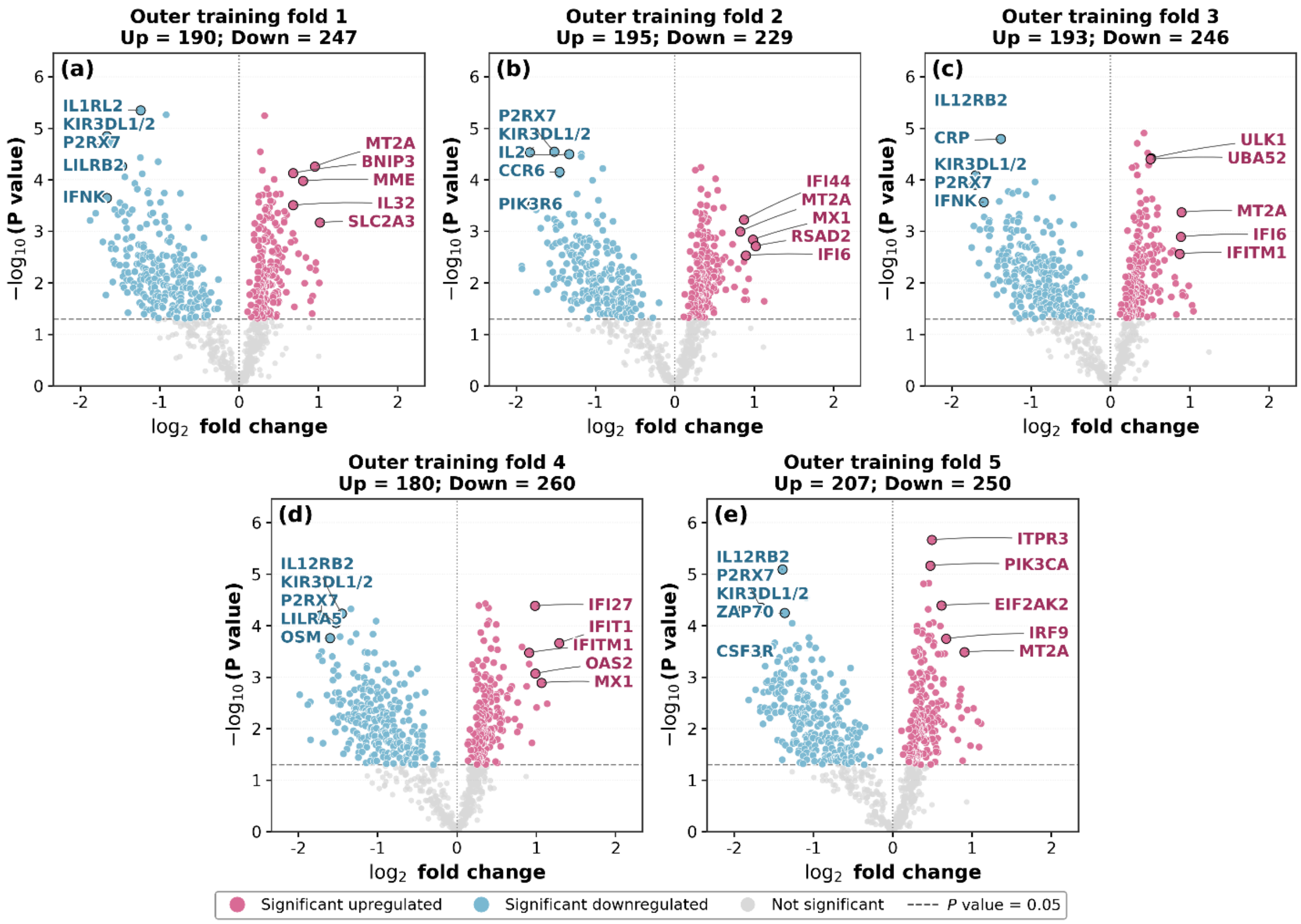

Figure S7. RSVA versus Mock-R at 72 h. *Volcano plots show RSVA relative to source-matched Mock-R within outer-training folds 1 through 5.*

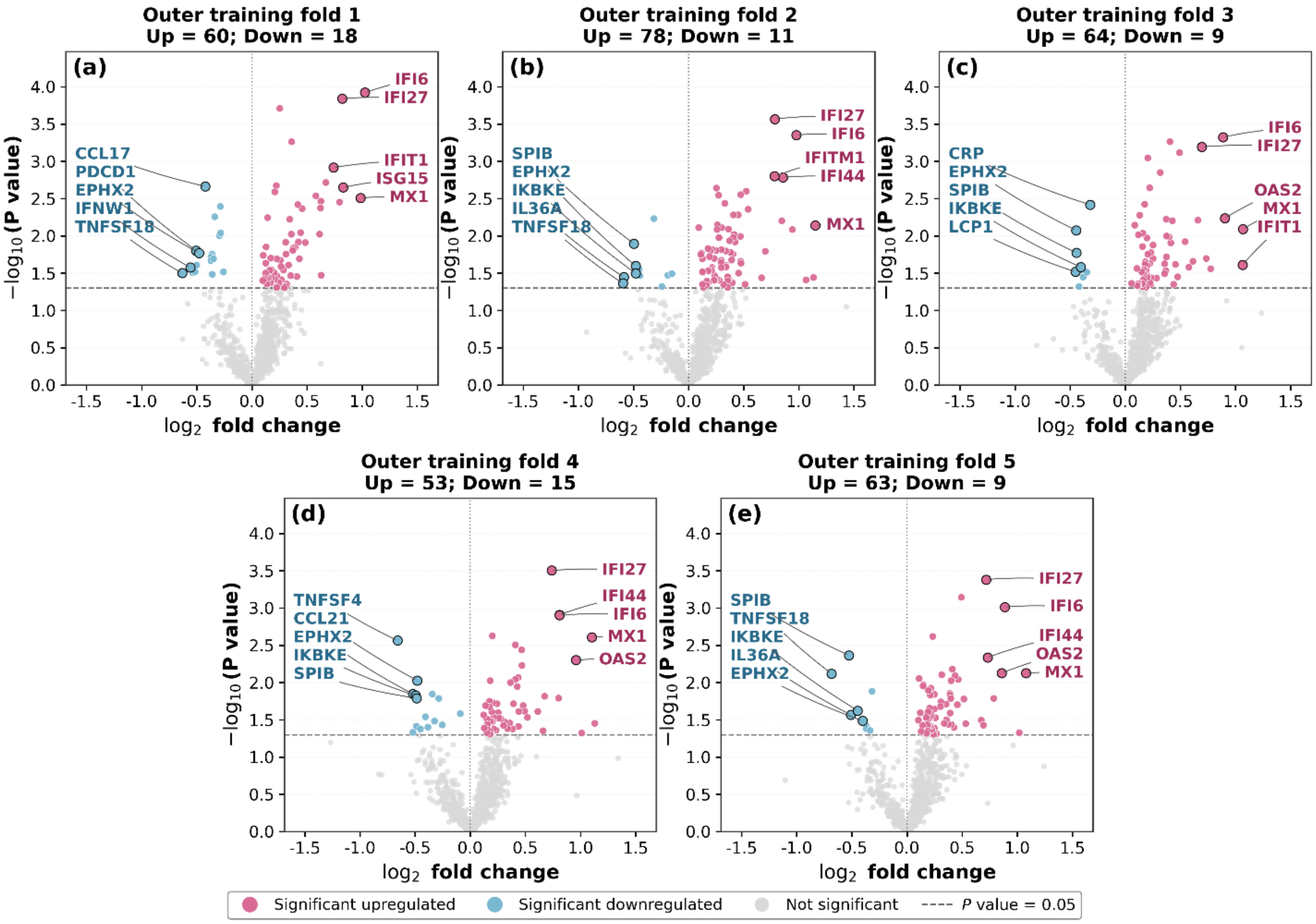

Figure S8. RSVB versus Mock-R at 72 h. *Volcano plots show RSVB relative to source-matched Mock-R within outer-training folds 1 through 5.*

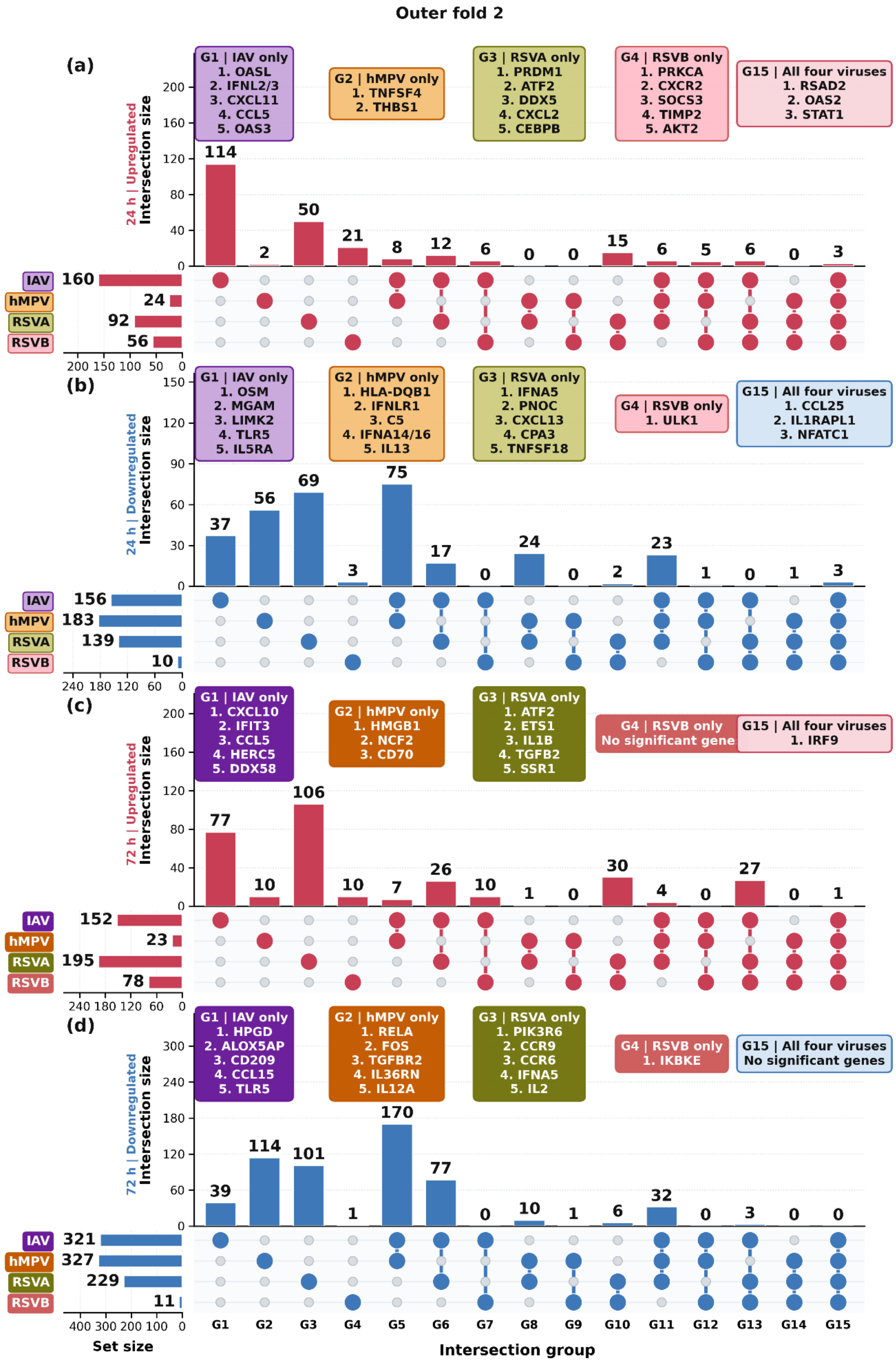

Figure S9. Direction-specific exact intersections of virus-associated genes in outer fold 2. *UpSet plots show exact intersections among genes significantly altered by IAV, hMPV, RSVA, and RSVB within the outer-training data of outer fold 2. Panels show upregulated and downregulated genes at 24 and 72 hours. Horizontal bars represent virus-specific set sizes, vertical bars represent exact intersection sizes, and the membership matrix identifies the viruses included in each intersection. Boxes list up to five leading genes from the virus-specific and PanViral MGSR candidate rankings.*

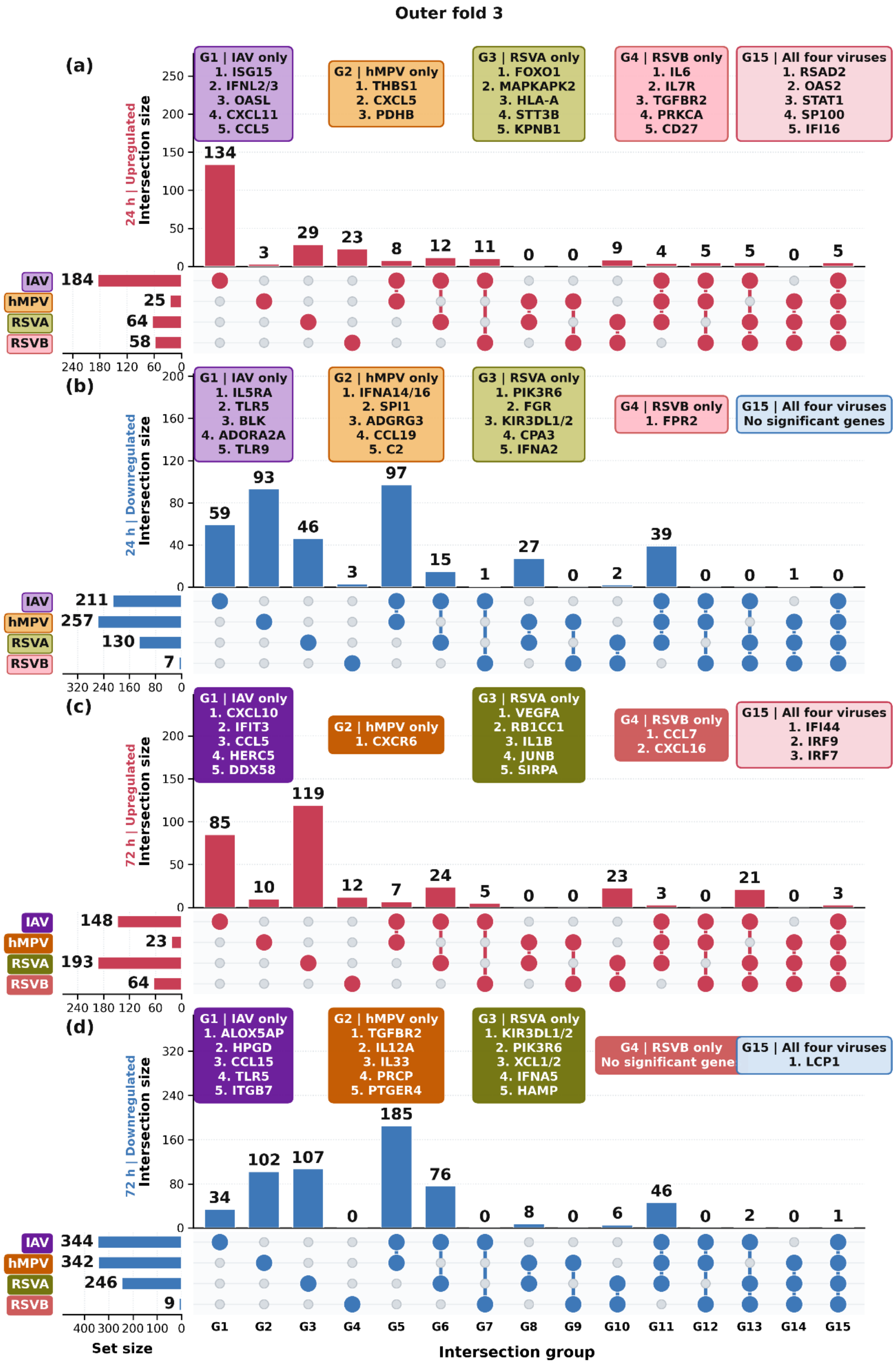

Figure S10. Direction-specific exact intersections of virus-associated genes in outer fold 3. *UpSet plots show exact intersections among genes significantly altered by IAV, hMPV, RSVA, and RSVB within the outer-training data of outer fold 3. Panels show upregulated and downregulated genes at 24 and 72 hours. Horizontal bars represent virus-specific set sizes, vertical bars represent exact intersection sizes, and the membership matrix identifies the viruses included in each intersection. Boxes list up to five leading genes from the virus-specific and PanViral MGSR candidate rankings.*

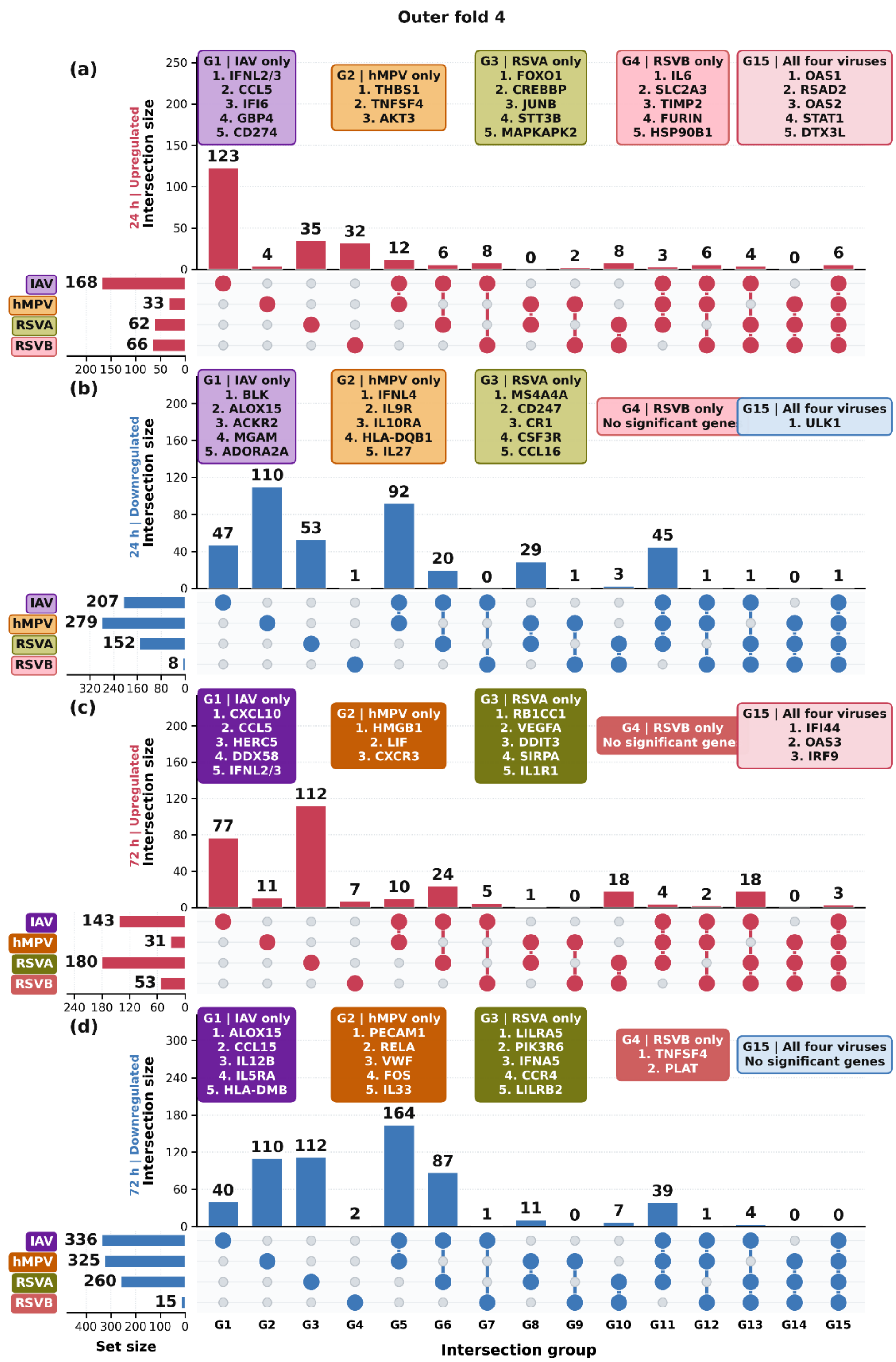

Figure S11. Direction-specific exact intersections of virus-associated genes in outer fold 4. *UpSet plots show exact intersections among genes significantly altered by IAV, hMPV, RSVA, and RSVB within the outer-training data of outer fold 4. Panels show upregulated and downregulated genes at 24 and 72 hours. Horizontal bars represent virus-specific set sizes, vertical bars represent exact intersection sizes, and the membership matrix identifies the viruses included in each intersection. Boxes list up to five leading genes from the virus-specific and PanViral MGSR candidate rankings.*

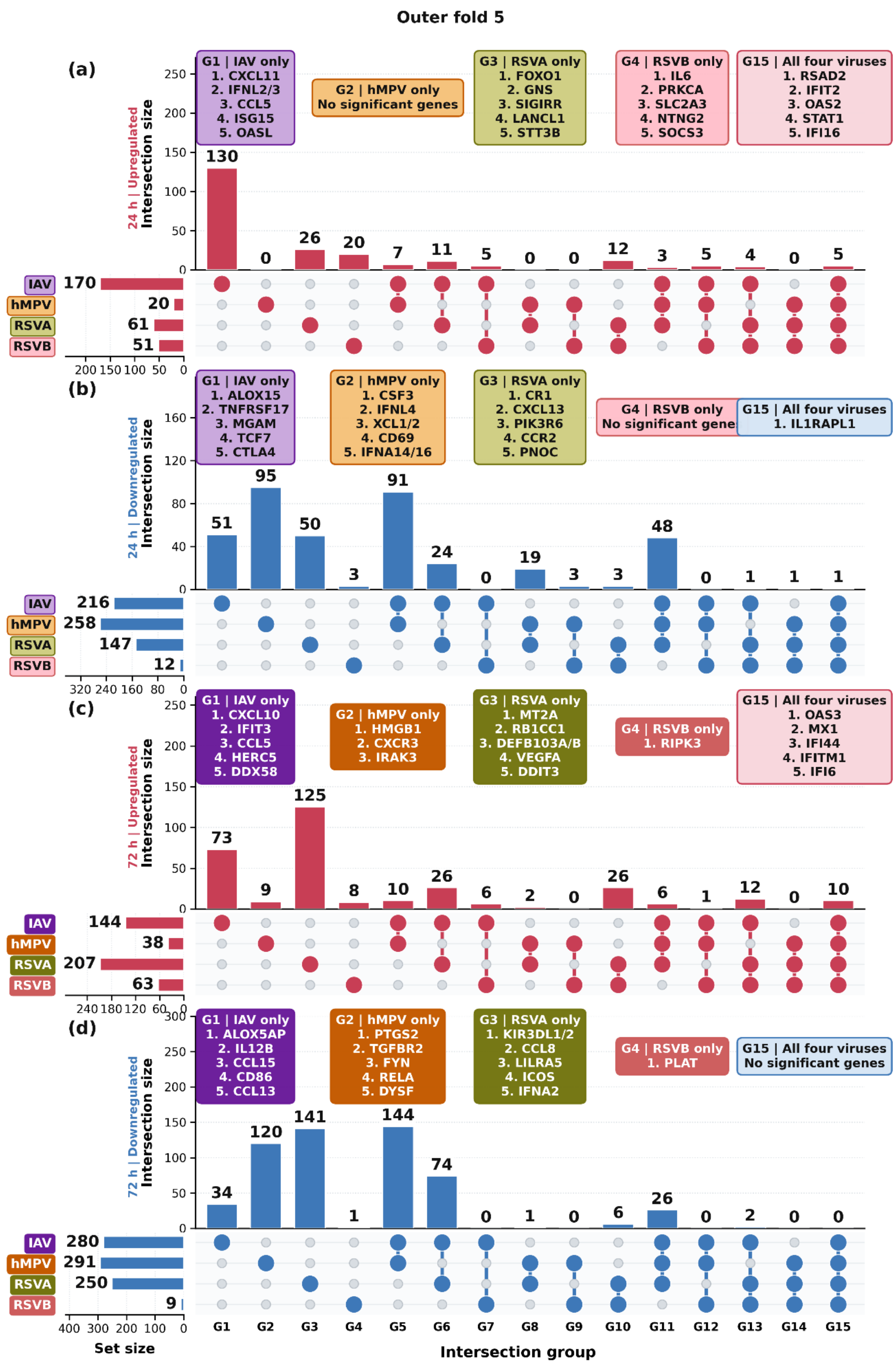

Figure S12. Direction-specific exact intersections of virus-associated genes in outer fold 5. *UpSet plots show exact intersections among genes significantly altered by IAV, hMPV, RSVA, and RSVB within the outer-training data of outer fold 5. Panels show upregulated and downregulated genes at 24 and 72 hours. Horizontal bars represent virus-specific set sizes, vertical bars represent exact intersection sizes, and the membership matrix identifies the viruses included in each intersection. Boxes list up to five leading genes from the virus-specific and PanViral MGSR candidate rankings.*

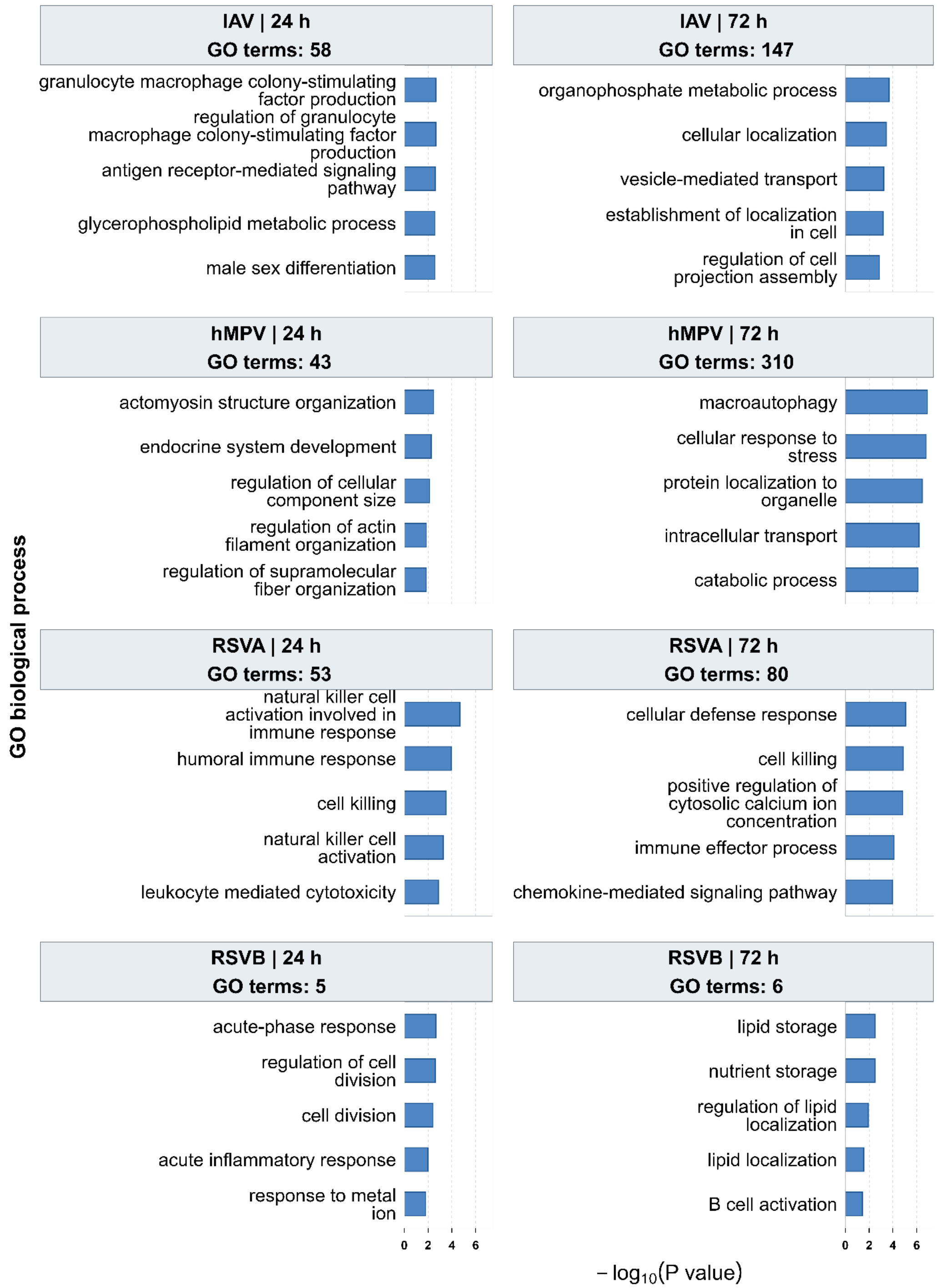

Figure S13. Leading GO Biological Process terms enriched among stably downregulated genes. *The five highest-ranked significant GO Biological Process terms are shown for IAV, hMPV, RSVA, and RSVB at 24 and 72 hours. The ribbon above each panel reports the total number of significant GO terms for the corresponding group. Bar length represents* $-log_{10}(P\ value)$.

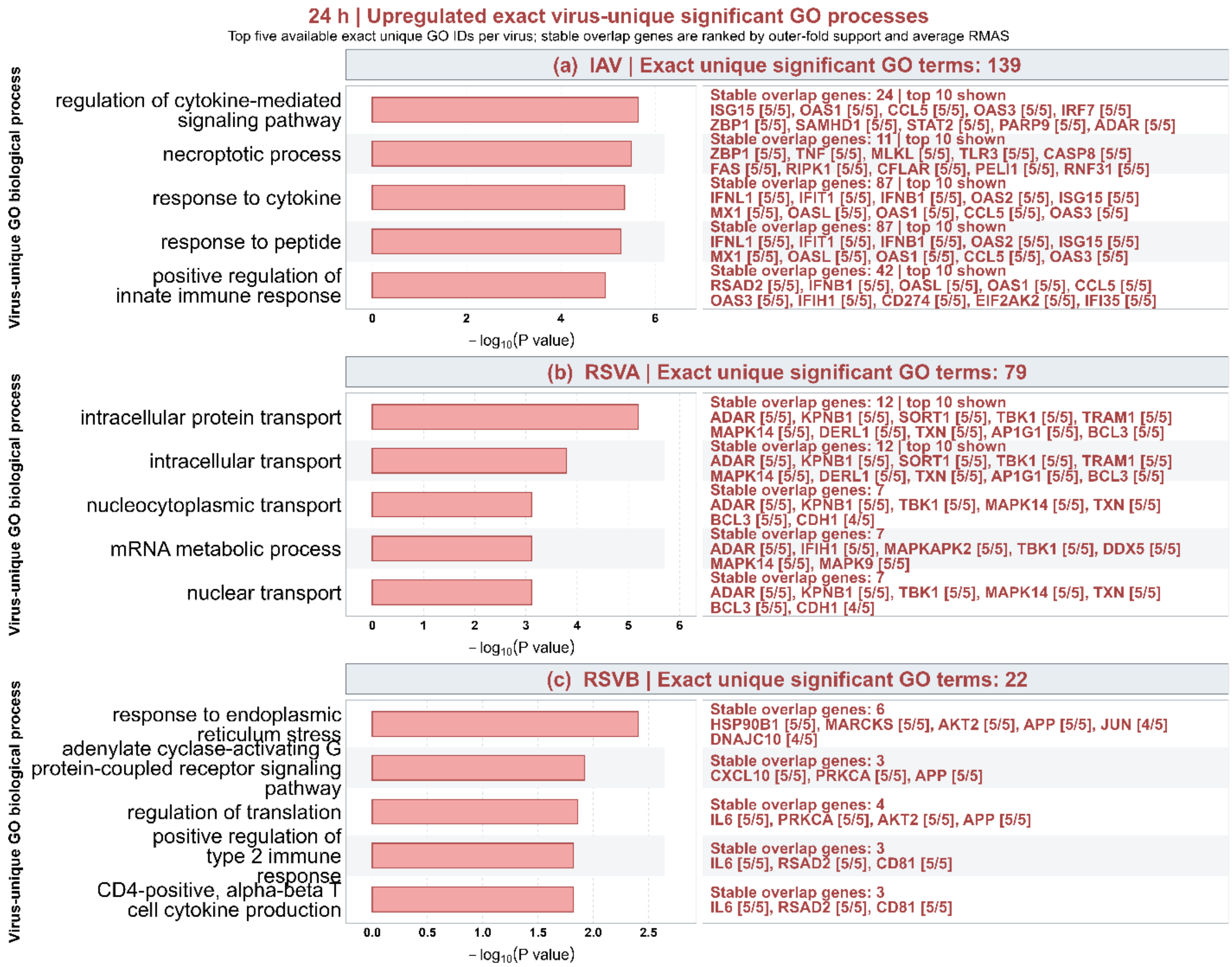

Figure S14. Exact virus-unique GO Biological Process terms among stably upregulated genes at 24 hours. *Panels show (a) IAV, (b) RSVA, and (c) RSVB. hMPV is omitted because no exact virus-unique upregulated GO term was identified at 24 hours. The ribbon reports the total number of exact unique terms, bars show the five highest-ranked terms, and the adjacent annotations list stable overlap genes and outer-fold support.*

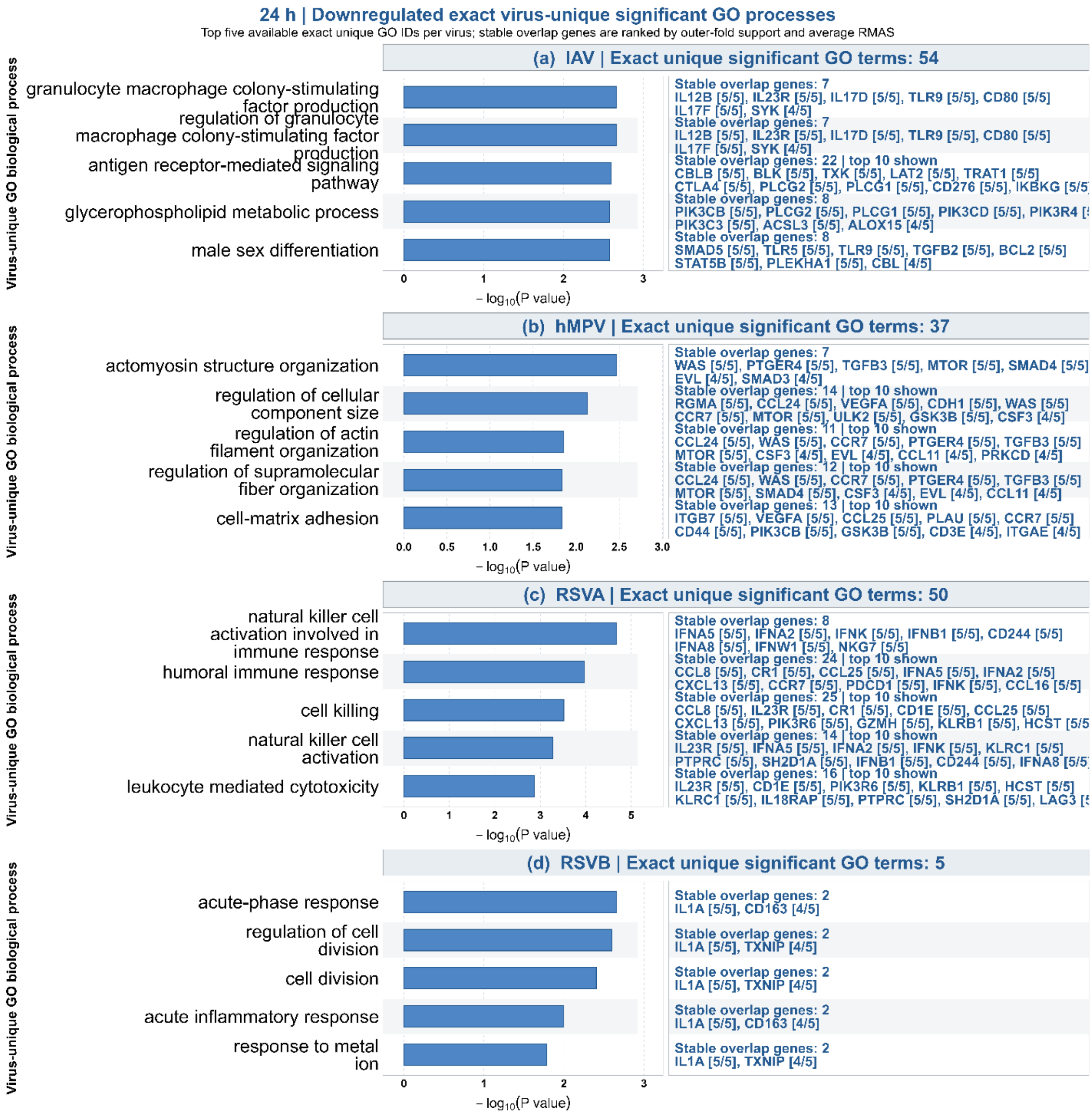

Figure S15. Exact virus-unique GO Biological Process terms among stably downregulated genes at 24 hours. *Panels show (a) IAV, (b) hMPV, (c) RSVA, and (d) RSVB. Exact virus-unique terms were identified within the 24-hour downregulated comparison. The ribbon reports the total number of exact unique terms, while the bars show the five highest-ranked terms. The right panel lists up to 10 stable overlap genes and their outer-fold support.*

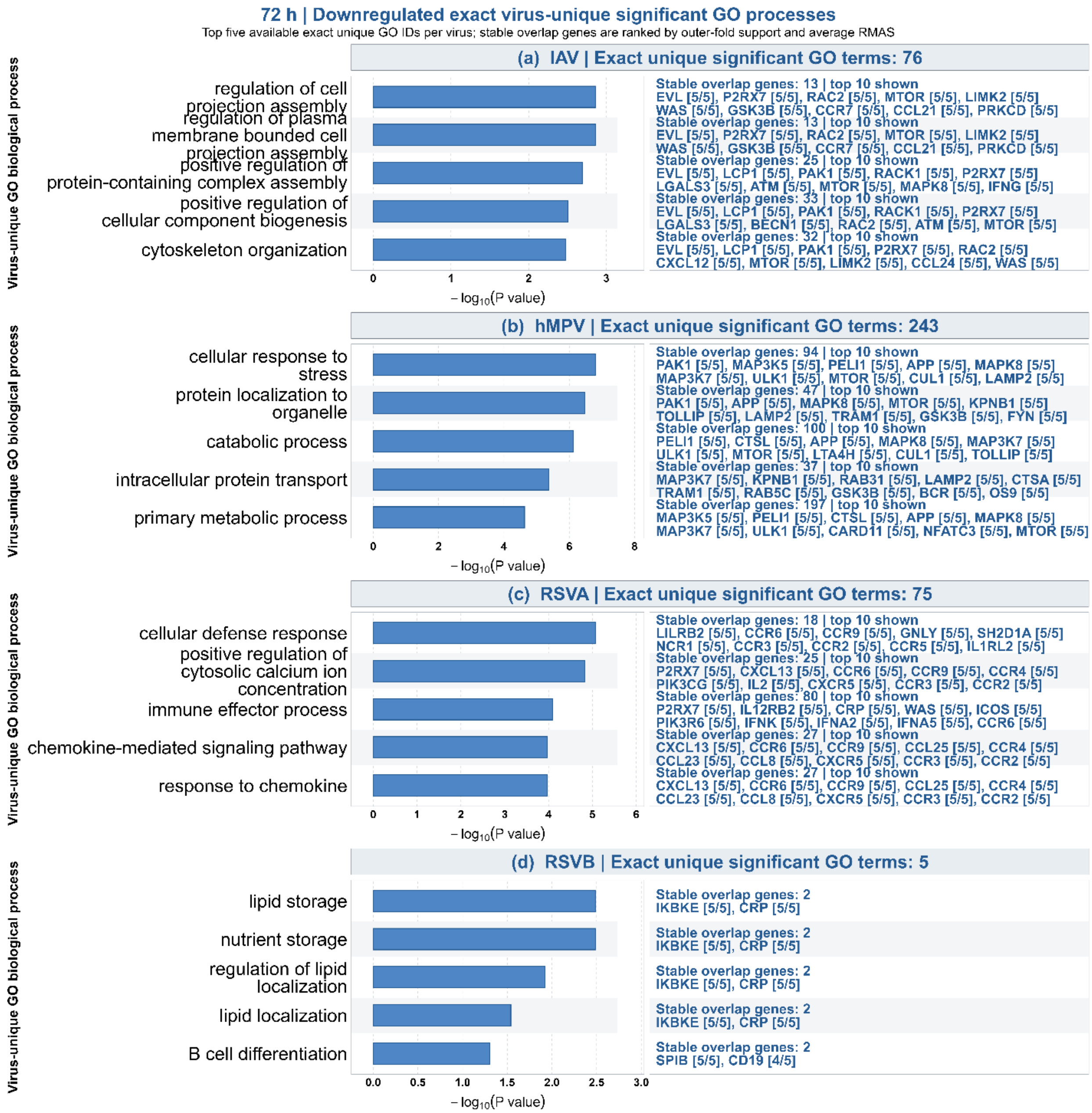

Figure S16. Exact virus-unique GO Biological Process terms among stably downregulated genes at 72 hours. *Panels show (a) IAV, (b) hMPV, (c) RSVA, and (d) RSVB. Exact virus-unique terms were identified within the 72-hour downregulated comparison. The ribbon reports the total number of exact unique terms, bars show the five highest-ranked terms, and the right panel lists up to 10 stable overlap genes and their outer-fold support.*
